# New Genetically Engineered Derivatives of Antibacterial Darobactins Underpin Their Potential for Antibiotic Development

**DOI:** 10.1021/acs.jmedchem.3c01660

**Published:** 2023-11-21

**Authors:** Carsten E. Seyfert, Alison V. Müller, Danica J. Walsh, Joy Birkelbach, Andreas M. Kany, Christoph Porten, Biao Yuan, Daniel Krug, Jennifer Herrmann, Thomas C. Marlovits, Anna K. H. Hirsch, Rolf Müller

**Affiliations:** † Helmholtz Institute for Pharmaceutical Research Saarland (HIPS), Helmholtz Centre for Infection Research (HZI) and Saarland University Department of Pharmacy, Saarbrücken 66123, Germany; ‡ German Centre for Infection Research (DZIF), partner site, Hannover, Braunschweig 38124, Germany; § Institute of Structural and Systems Biology and Centre for Structural Systems Biology (CSSB), University Medical Center Hamburg-Eppendorf (UKE), Hamburg 22607, Germany; ∥ Deutsches Elektronen-Synchrotron Zentrum (DESY), Hamburg 22607, Germany; ⊥ Helmholtz International Lab for Anti-Infectives, Saarbrücken 66123, Germany; # Centre for Structural Systems Biology (CSSB), Hamburg 22607, Germany

## Abstract

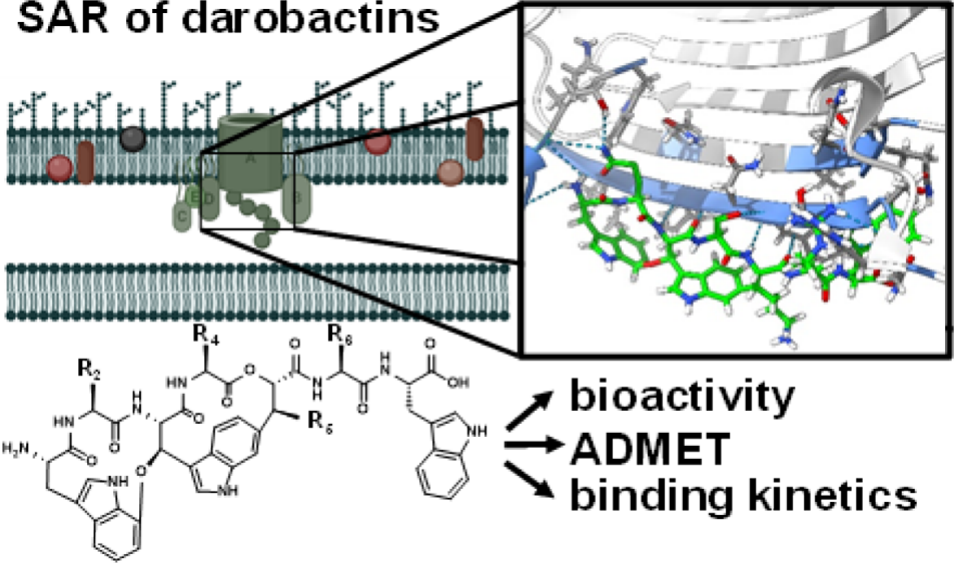

Biosynthetic engineering of bicyclic darobactins, selectively sealing the lateral gate of the outer membrane protein BamA, leads to active analogues, which are up to 128-fold more potent against Gram-negative pathogens compared to native counterparts. Because of their excellent antibacterial activity, darobactins represent one of the most promising new antibiotic classes of the past decades. Here, we present a series of structure-driven biosynthetic modifications of our current frontrunner, darobactin 22 (**D22**), to investigate modifications at the understudied positions 2, 4, and 5 for their impact on bioactivity. Novel darobactins were found to be highly active against critical pathogens from the WHO priority list. Antibacterial activity data were corroborated by dissociation constants with BamA. The most active derivatives **D22** and **D69** were subjected to ADMET profiling, showing promising features. We further evaluated **D22** and **D69** for bioactivity against multidrug-resistant clinical isolates and found them to have strong activity.

## Introduction

**Figure 1:**
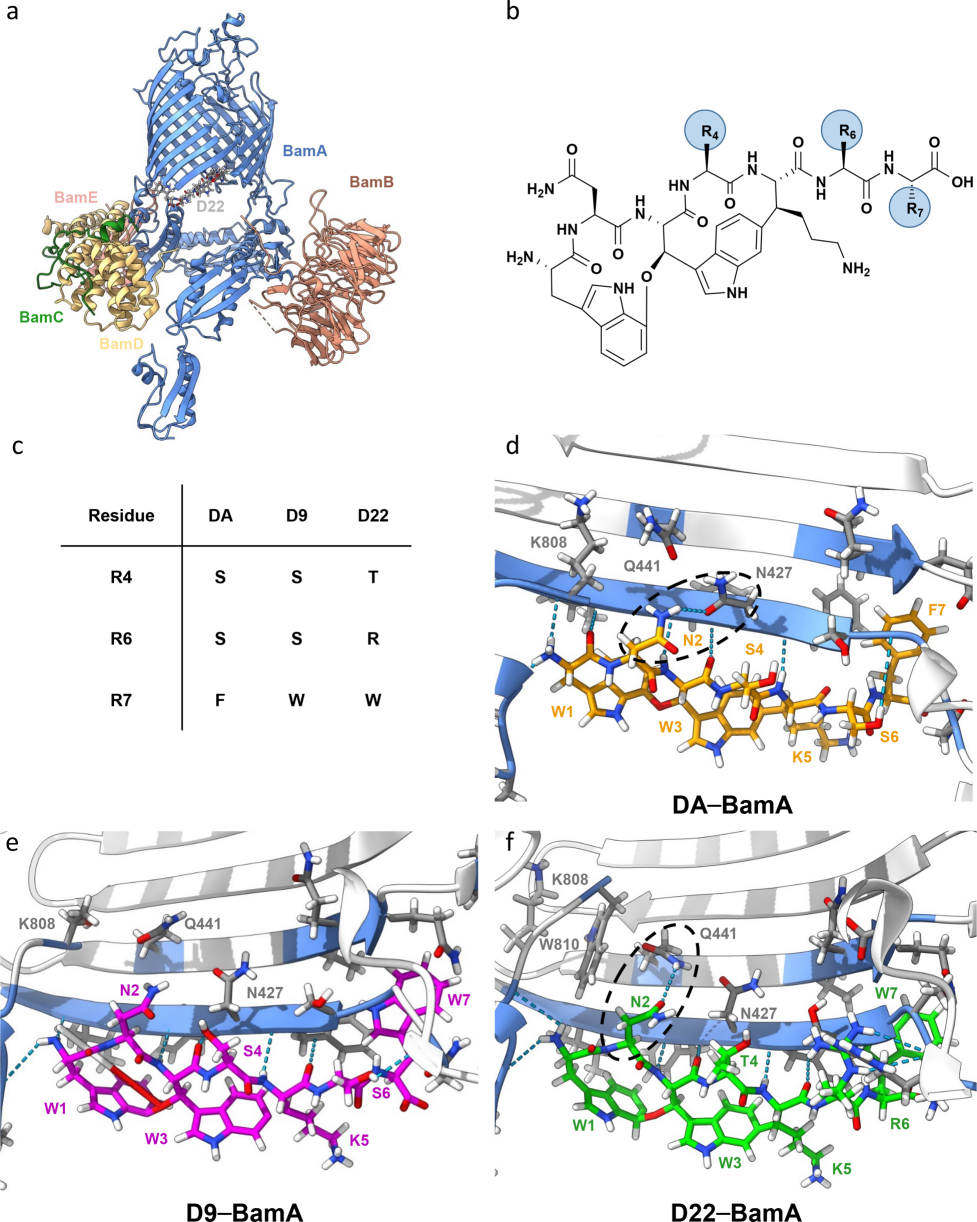
Analysis of darobactin–BamA interaction. (a) Costructure of **D22–**BamABCDE (BAM). (b) Darobactin core structure with residues 4, 6, and 7 highlighted (R_4_, R_6_, and R_7_). (c) Overview of variations between native **DA** and derivatives **D9** and **D22** on residues 4, 6, and 7, respectively. (d) BamA**–DA** (orange) interaction site. (e) BamA**–D9** (pink) interaction site. (f) BamA**–D22** (green) interaction site. Calculated hydrogen bonding interactions between BamA and darobactins are shown in blue. The raw data were taken from the Protein Data Bank (PDB) and originally published by Kaur et al.^14^ and Seyfert et al.^15^ PDB accession codes: 7NRI (**DA**), 8ADI (**D9**), and 8ADG (**D22**).

In the coming years, increasing antimicrobial resistance (AMR) is expected to induce a rise in the number of deaths of the growing global population from roughly 1.3 million to up to 10 million annually.^1−5^ In particular, resistant pathogens appear in clinics more often, among other factors in combination with nosocomial infections, leading to increased mortality rates.^5−7^ Most new antibiotics reaching the development stage are derivatives of known chemical classes with a known target and mode of binding (MoB), resulting in potentially rapid development of cross-resistance.^8−10^ Thus, the search for antibacterial compounds acting on innovative target sites is urgently required. The recently discovered darobactins are a novel class of antibiotics showing promise for meeting that demand.^11,12^ Native darobactins found in *Photorhabdus khanii* are ribosomally produced and post-translationally modified peptides (RiPPs).^11,13^ They selectively target the outer membrane protein (OMP) BamA, the major component of the BamABCDE (BAM) complex, and inhibit the insertion and folding of OMPs, ultimately resulting in cell lysis.^14−18^ The target site of darobactins is not addressed by commercially available antibiotics. Consequently, the risk of cross-resistance is lower, and they are proven to exhibit an auspicious broad-spectrum Gram-negative activity against clinically relevant and multiresistant pathogens such as *Pseudomonas aeruginosa* (*P. aeruginosa*), *Escherichia coli* (*E. coli*), *Acinetobacter baumannii* (*A. baumannii*), or *Klebsiella pneumoniae* (*K. pneumoniae*), against which many other antibiotics are not effective anymore.^11,12,15,19,20^ Two derivatization series of darobactins published by Groß et al. and Seyfert et al. have confirmed the efficacy of scaffold modification by biotechnological engineering of the fully synthetic darobactin biosynthetic gene cluster (BGC) to enhance the bioactivity of analogues,^12,15^ which was partly confirmed by the study of Marner et al.^21^ The feasibility of modifications at specific positions of the core peptide was a prerequisite for analyzing their influence on antibacterial activity and for understanding how analogues of this novel compound class interact with its unique target BamA. These new insights together with the first described cocrystal structure of darobactin A (**DA**) and BamA helped establishing the structure–activity relationships of the different derivatives.^14,15^ The earlier derivatization series increased the antibacterial activity of darobactins up to 128-fold for darobactin 22 (**D22**) compared to native **DA**, even against clinical isolates of carbapenem-resistant *A. baumannii* (CRAB) and multidrug-resistant *P. aeruginosa*.^12,15,21^ However, some positions, especially position 2 (Figure 1), have not yet been subject to detailed investigation. Additional efforts to engineer antibacterial darobactin derivatives as potential future drugs are therefore a promising approach to fight the antibiotic crisis.^22^

Consequently, we used our current frontrunner molecule **D22** as a starting point and considered the effects of changes at position 2 alone as well as designed and produced analogues with additional modifications of positions 4, 5, and 6. We devised 17 novel darobactins based on the results achieved by evaluating the structure- and activity-guided outcome of our previous study^15^ in order to explore the influence of amino acid changes in so far underinvestigated positions. Notably, we found that changes at positions 2 and 5 still result in highly active darobactins, inconsistent with data from previous publications regarding native darobactin D (**DD**) and darobactin E (**DE**).^23^ Changes at position 4 support the hypothesis that the exchange of l-serine and l-threonine may affect activity due to a shift in molecular orientation. One new derivative showed activity comparable to **D22** with better binding to the target BamA. The first *in vitro* ADMET analyses reveal a favorable profile of the tested frontrunners, enabling the selection of a suitable derivative for further *in vivo* profiling.

## Results

### Structural Analysis of Darobactin–BamA Interaction Sites and Design of Novel Analogues Potentially Interacting with BamA

The structural elucidation of darobactin MoB using cryo-EM and crystallization techniques shed light on the complex structure of BAM–**DA** and BAM–**D9**.^14,15^ Combined with an activity-guided approach, this led to highly active analogues like **D22** (Figure 1a, c, and f).^15^ Subsequently, we further analyzed the BAM–darobactin costructures to devise a blueprint for developing novel analogues, modified on previously underinvestigated positions of the darobactin heptapeptide. We compared the different interactions of **DA**, **D9**, and **D22** with BAM, utilizing the respective recently solved costructures (Figure 1d–f).^14,15^ In particular, variations at positions 4, 6, and 7 of the heptapeptide (Figure 1b,c) seem to affect the hydrogen bonding interactions of l-asparagine at position 2 for **DA** (N427), **D9** (no interaction), and **D22** (Q441) (Figure 1d–f). Therefore, the observed variability of interactions with position 2 makes this position particularly suitable for introducing amino acid modifications to alter interactions with Q441 on β-sheet 2 or N427 on β-sheet 1 (Figure 1).

**Figure 2:**
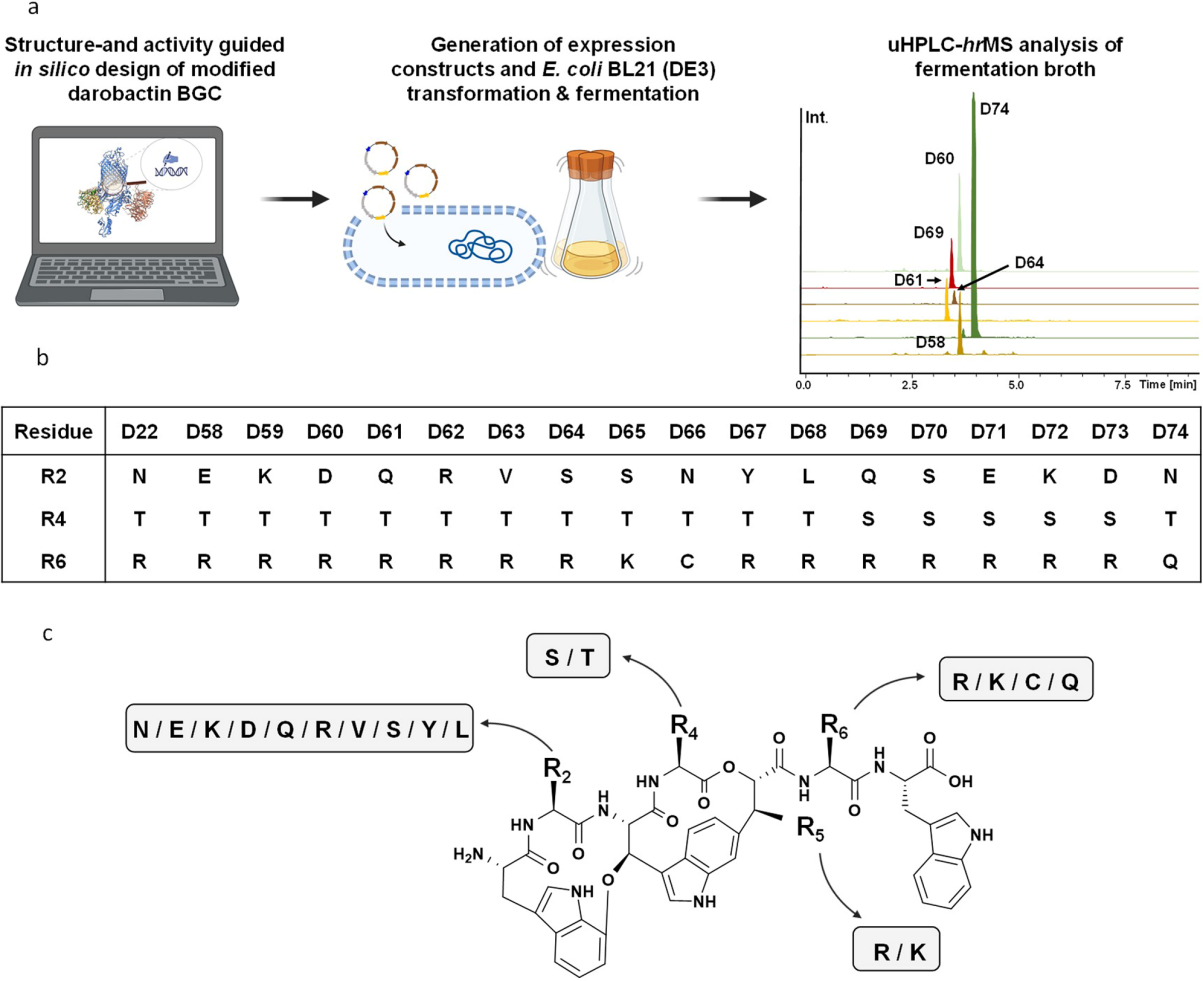
Structure- and activity-guided approach to produce novel darobactins**.** (a) Workflow to design, produce, and analyze novel derivatives. (b) Novel derivatives **D58** to **D74** with modifications at positions 2, 4, and 6. (c) Predicted chemical structure of the darobactins, investigated in this study using MS^2^ analysis (Figures S20–S38 and Table S5): **D58** to **D74** and the previously published derivative **D39.**(15)

Consequently, we initiated a structure-guided mutagenesis of **D22** mainly at position 2 but also including positions 4, 5, and 6 to evaluate the potential of various amino acids to build hydrogen bonding interactions with BamA. We used the Chimera^24^ and PyMOL^25^ rotamer and mutagenesis tools to model exchange of amino acids of **D22** within the costructure with BamA, which prompted us to explore interactions between BamA and hitherto untested amino acid residues, varying in length and polarity (Figure 2a). Examples of modeled BamA–darobactin interactions are presented in Figures S1a–f and S2a–c. Briefly, changing l-asparagine to l-glutamic acid, l-aspartic acid, l-glutamine, or l-tyrosine in derivatives **D58**, **D60**, **D61**, **D67**, and **D69**, respectively, might result in hydrogen bonding formation between amino acids on the first or second BamA β-sheet. Depending on the automated calculation, even multiple interactions are possible as computed for **D69** (Figure S2a,b). Furthermore, after a switch from l-asparagine to l-serine at position 2 similar to native darobactin **DE**,^11,12^ the l-serine of the resulting **D64** does not appear to be involved in a hydrogen bonding interaction with BamA. However, we could not identify a steric hindrance that would allow conclusions about the rather inactive native analogue **DE** (WSWSKSF)^23^ (Figure S1e). The previously detected and discussed orientation shift,^15^ potentially due to change from l-serine to l-threonine at position 4, was also intended to be evaluated for its influence on antibacterial activity by directly comparing several derivatives that differed only in position 4 (**D69–D73**). Further, the hydrogen bonding interactions between BamA and **D39**, a previously described analogue that was not further investigated, are not directly influenced by changing l-lysine in position 5 to l-arginine according to our results (Figure S1a). Since the change at position 6 has previously led to greater differences in antibacterial activity that seem to be partly correlated with variations in the production,^12,15^ we also sought to generate derivatives that were characterized by changes at position 6. A less active derivative could possibly have higher production rates due to the reduced intrinsic sensitivity of the Gram-negative bacterial producer. Thus, derivatives **D65** with l-lysine and **D74** with l-glutamine in this position were designed (Figure 2b and Figure S2c).

In total, 17 novel derivatives were modeled and designed *in silico* (Figure 2a,b). Since the novel analogues were engineered mainly based on modeled structures combined with the activity data as found in the literature,^11,12,15,23^ we wanted to corroborate our hypotheses. Considering the still rather time-consuming production and purification of darobactins, we characterized the novel darobactins following the rational cascade of (1) investigating the influence of altered amino acids on the bioactivity as observed from extracts of producing strains, (2) confirming the bioactivity of selected derivatives as pure compounds, and (3) profiling the most active derivative in comparison to **D22**.

We generated the new darobactin expression constructs by introducing point mutations in the plasmid pNOSO–darABCDE–22, as described previously.^15^ We achieved the production in *E. coli* BL21 (DE3) and extraction and analysis of darobactins as described in the methods (Figure 2a,b). We assessed the compound production using mass spectrometry (MS) (Table S4 and Figures S3–S19) and detailed MS^2^ fragmentation pattern analysis (Table S5 and Figures S20–S37) to validate the respective compounds. Varying molecular ions were detected in electrospray ionization (ESI) MS measurements (Tables S4 and S5), consistent with previous results.^15^ All derivatives could indeed be identified, and the MS^2^-predicted chemical structures of all novel darobactins are displayed in Figure S38.

### Evaluation of Antibacterial Activity, Binding Kinetics, and Cytotoxicity of Novel Darobactins

The extracts of production clones for all derivatives were prepared and tested for their antibacterial activity against *E. coli* ATCC 25922, *P. aeruginosa* PA01, *K. pneumoniae* DSM-30104, and *A. baumannii* DSM-30008. The production of new darobactins was estimated based on integration of combined extracted ion chromatograms (EICs) of singly, doubly, and triply charged darobactin ions in the crude extracts and was normalized to **D22** due to variable ionization states of most prominent mass peaks^15^ (Table S4) and is presented in Table 1.

**Table 1:**
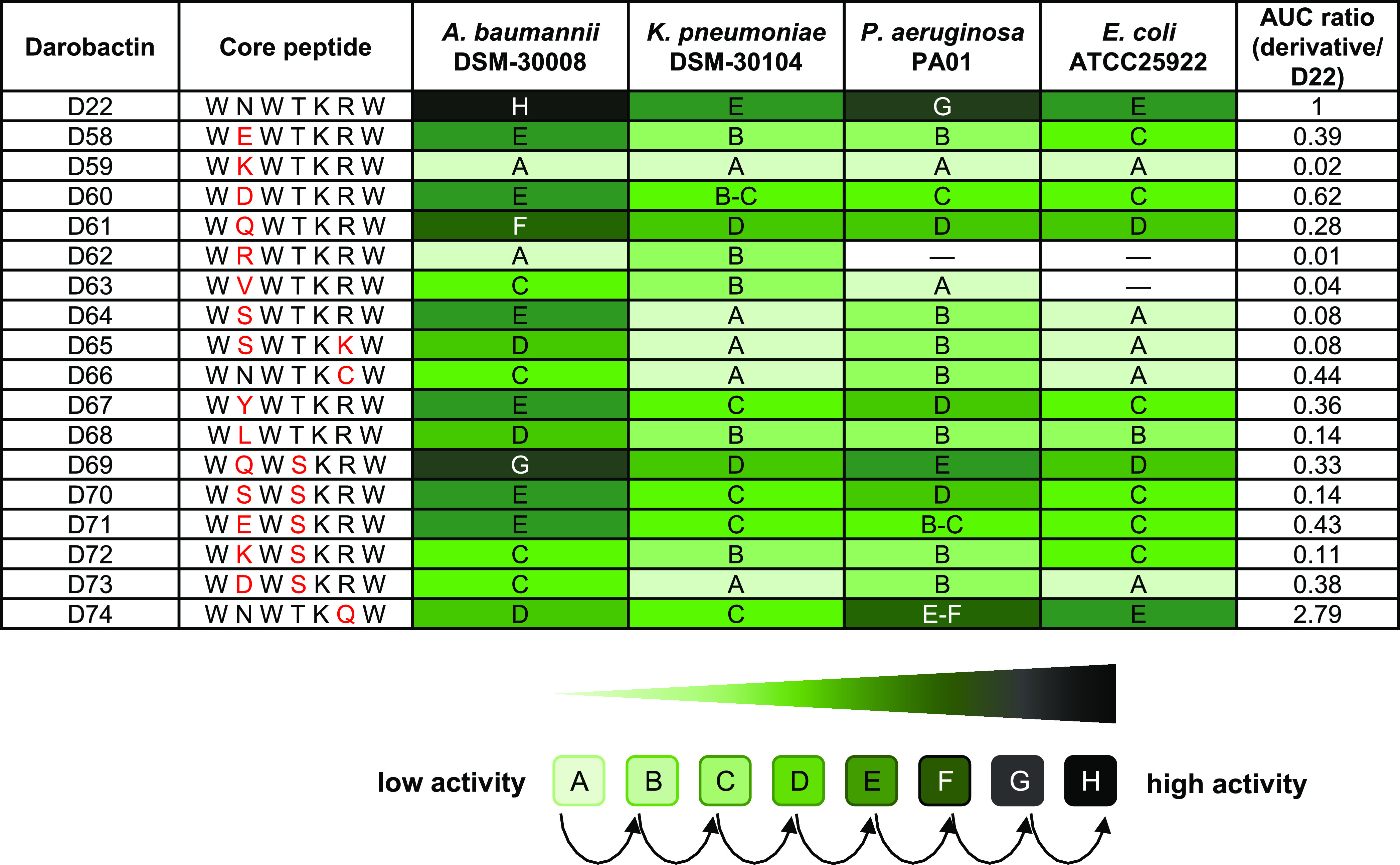
Antibacterial Activity Screening of Darobactin-Containing Extracts with Modified Core Peptide Sequences against *Acinetobacter baumannii* DSM-30008, *Klebsiella pneumoniae* DSM-30104, *Pseudomonas aeruginosa* PAO1, and *Escherichia coli* ATCC 25922^a^

a Changes in the core peptide sequence compared to the **D22** sequence are shown in red. Relative production titers of **D22** and its derivatives in crude extracts were calculated by comparing the area under the curve (AUC) ratios of new derivatives relative to **D22**. The antimicrobial activity of each derivative-containing crude extract against tested pathogens is highlighted as a color code (bright green to dark green), depending on the highest dilution factor of standardized extract with respect to the assay volume [two-fold serial dilution from 1:15 (A) (concentration factor: 6.67×) to 1:3,840 (H) (concentration factor: 0.05×)] in which full growth inhibition was detected (compare to Seyfert et al.^15^). UHPLC-HRMS chromatograms of produced new darobactins are shown in Figures S3–S19.

All extracts containing new analogues were active against the panel of Gram-negative pathogens tested, and species specificity resembles the earlier findings for derivatives **D9** and **D22**.^12,15^ Antibacterial activity differs from extract to extract but agrees with significantly varying production, as determined via the area under the curve (AUC) ratio calculated from LC–MS analysis and normalized to **D22** production (Table 1). Interestingly, the changes at position 2 result in highly active analogues (*e.g.,***D58**–**D65** and **D67**–**D73**). The derivatives with changes at position 2 and an l-serine instead of l-threonine at position 4 exhibit increased antibacterial activity in extracts, but this might also be due to the different production levels (see the AUC ratio in Table 1). Therefore, we selected eight representative derivatives for purification in order to conduct bioactivity profiling with pure compounds and to avoid misinterpretation of the MIC data from extracts that could arise due to significantly varying production titers. The **D22** analogues were produced on a larger scale and purified. Next, the pure darobactins **D58**, **D60**, **D61**, **D64**, **D69**, and **D74** were tested against the same panel of pathogens. In addition, **D39** was produced, purified, and tested in parallel to study the contradicting MIC data regarding the inactivity of native **DD**(23) compared to **D39**(15) but also to compare it with published data of active **DA**.^11,12^ The activity data of all pure compounds align well with the MIC data obtained from derivative-containing extracts (Table 1). All novel darobactins exhibit promising antibacterial activity against a range of clinically relevant pathogens (Table 2). Variations at position 2 unequivocally lead to active derivatives in contrast to the activity profile of native darobactin **DE**. The analogues **D58**, **D60**, **D61**, **D64**, and **D69** retain high activity against *A. baumannii*, but the changes at position 2 result in slightly different activity profiles against *K. pneumoniae*, *P. aeruginosa*, and *E. coli* (Table 2). However, the antibacterial activity of all of the derivatives remains potent against the tested pathogens. Derivative **D39**, which was not tested as a pure compound in a previously published assay,^15^ with l-arginine instead of l-lysine at position 5 as in native analogue **DD**, displayed lower activity against *P. aeruginosa* and *K. pneumoniae* compared to **D22**. However, its activity is still of low micromolar and not completely abolished, as described for native **DD** (WNWSRSF) by Böhringer et al.^23^ Derivative **D39** even exhibits activity against the tested *A. baumannii* strain comparable to the current frontrunner **D22**.

**Table 2:**
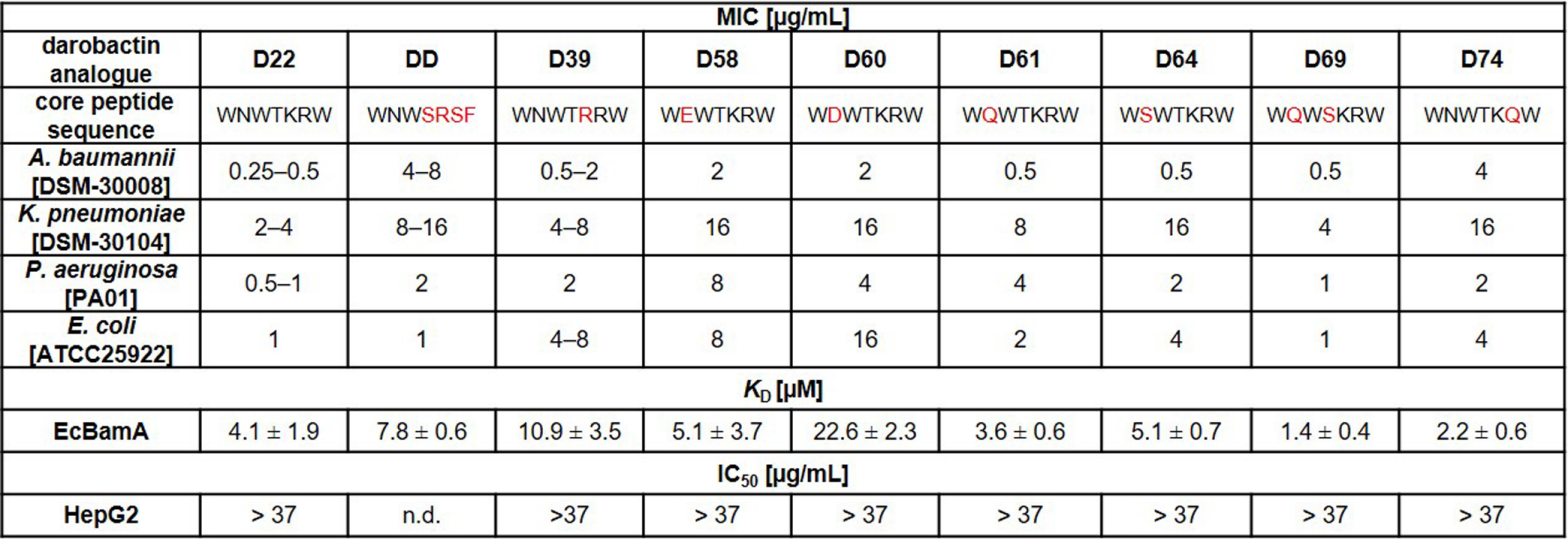
Minimal Inhibitory Concentration (MIC), Binding Kinetics (*K*_D_), and Cytotoxicity of **D22** (Control), Novel Analogues **D39**, **D58**, **D60**, **D61**, **D64**, **D69**, and **D74**, and the Native Derivative **DD**^a^

a Antibacterial activity [in μg mL^**–**1^; *n* = 2] is given for representative strains of the most critical and clinically relevant Gram-negative pathogens *Acinetobacter baumannii*, *Klebsiella pneumoniae*, *Pseudomonas aeruginosa*, and *Escherichia coli*. Binding kinetics represented by determination of *K*_D_ values (in μM) were determined via microscale thermophoresis for selected darobactins against BamA of *E. coli*; *n* = 3. No toxicity was observed against the tested human cell line HepG2; *n* = 2; n.d. = not determined.

The inconsistency between the published antibacterial activities of **DD** and those of **D39** prompted us to produce **DD** in our laboratory. We obtained 13 mg of pure **DD** via isolation out of 9 L of formulated medium (FM), in contrast to the yield of ∼1 mg out of 100 L as described by Böhringer et al.^23^ This compound supply enabled evaluation of MICs (Table 2), which revealed high antibacterial activity for **DD** (Table 2), significantly different from previously published data.^23^ Surprisingly, direct comparison between **D61** and **D69**, only differentiated by the l-serine/l-threonine change at position 4, shows partly enhanced activity of **D69** comparable to the current frontrunner **D22**. Further, the substitution of l-arginine to l-glutamine in **D74** affects the antibacterial activity negatively, as expected by analyzing the modeled costructure of BamA–**D74** compared to the cryo-EM data of BamA–**D22**: **D74** is less anchored at BamA than **D22** at position 6 (Figure S2c). Of note, the available amount of **D74** was significantly higher in the extract produced for initial activity assessment in comparison to the other new derivatives (Table 1), and we could quantify in follow-up experiments that the production indeed reached 33 ± 1.7 mg per L medium.

The production of **D22**(15) achieved 10.5 ± 0.9 mg per L, of **D58** 6.9 ± 2.0 mg per L, of **D61** 7.4 ± 1.1 mg per L, of **D69** 4.6 ± 0.6 mg per L, and of **DD** 4.4 ± 1.1 mg per L (Figure S39).

**Figure 3:**
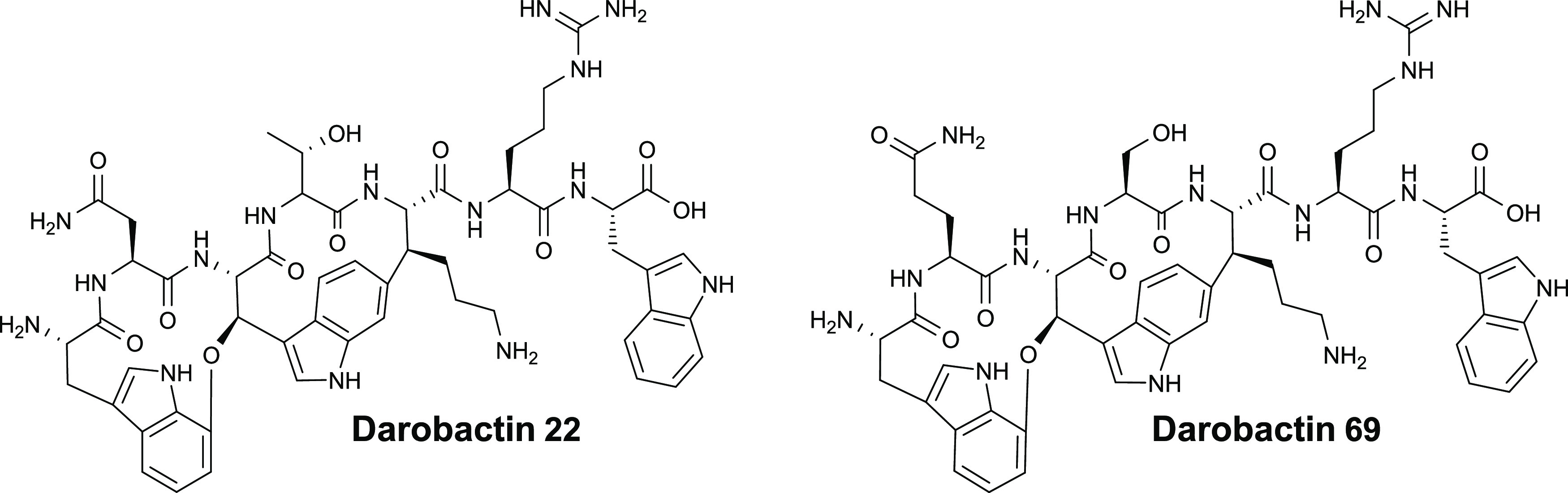
Chemical structures of darobactins **22** and **69**.

Due to the unexpectedly differing activity data, we evaluated binding constants (*K*_D_) of each purified analogue to *E. coli* BamA (EcBamA). As an alternative to the previously published methods using isothermal titration calorimetry (ITC), we established a *K*_D_ determination assay using microscale thermophoresis (MST) to enable higher throughput. The *K*_D_ data underpin the results of the activity screen (Table 2): *K*_D_ of all tested derivatives in fact correlates with MIC data. We observed a slightly better *K*_D_ for **D69**, bearing an l-glutamine instead of l-arginine, compared to **D22** (Figure 3). Thus, we decided to use the best derivative of our new series, **D69**, to compare its ADMET profile with **D22**, which has not been analyzed before. Moreover, we verified the chemical structure of **D69** via NMR (Figure 3, Table S7, and Figures S39–S44).

### *In Vitro* ADMET Profiling of **D22** and **D69**

The two most promising darobactin analogues, **D22** and **D69**, were evaluated regarding metabolic and plasma stability as well as plasma protein binding (PPB) in order to obtain first *in vitro* information on their pharmacokinetic properties (Table 3).

**Table 3:** Determination of Metabolic Stability, Plasma Stability, and Plasma Protein Binding^f^

**darobactin analogue**	**species**	**D22**	**D69**
liver microsomes *t*_1/2_^a^ [min]/Cl_int_^b^ [μL/mg/min]	mouse^d^	>120/<11.6	>120/<11.6
	human	>120/<11.6	>120/<11.6
	rat	>120/<11.6	>120/<11.6
plasma *t*_1/2_ [min]	mouse^e^	>240	>240
	human	>240	>240
	rat	>240	>240
PPB^c^ [%]	mouse^e^	57.5 ± 7.2	53.6 ± 10.3
	human	46.7 ± 2.7	53.1 ± 3.7
	rat	62.8 ± 6.3	58.4 ± 7.4

a Half-life.

b Intrinsic clearance.

c Plasma protein binding.

d C57BL/6.

e CD-1.

f Darobactin derivatives **D22** and **D69** were compared in mouse, human, and rat (Wistar) liver microsomes/plasma.

Both, **D22** and **D69**, were not metabolized in murine liver microsomes over 2 h. Likewise, both compounds showed good plasma stability with no degradation over 4 h. This was also confirmed in human and rat fractions and plasma for both compounds. PPB was found to be relatively low between 46 and 63%, which is correlated to the high polarity and good solubility of these RiPPs. Furthermore, all new darobactins investigated in this study did not display cytotoxicity against the human cell line HepG2, which is consistent with previous results^11,12,15^ where no toxicity *in vitro* and even *in vivo* has been detected at concentrations up to 37 and 500 μg mL^–1^, respectively.

### Activity Screening against Multidrug-Resistant *A. baumannii* and *P. aeruginosa* Strains to Select the Most Attractive Candidate for Further *In Vivo* Profiling

Considering the superior binding affinity of **D69**, along with comparable ADMET (Table 4) and MIC data for **D22** and **D69** (Table 2), we aimed to assess the potency of **D22** and **D69** against further *A. baumannii* and *P. aeruginosa* strains, including multidrug-resistant clinical isolates in addition to laboratory strains. Therefore, we selected human pathogens isolated from the lungs and urine of patients with indwelling catheters (*P. aeruginosa* 83979 and *P. aeruginosa* 84389), which are challenging to treat and exhibit resistance to four classes of antibiotics: acylureidopenicillins, cephalosporins, fourth-generation carbapenems, and fluoroquinolones (Table S6). **D22** tends to show better antibacterial activity in the sub- to low-micromolar range against the tested hard-to-treat pathogens (Table 4). However, the antibacterial activity of both **D22** and **D69** against *P. aeruginosa* 83979 and 84389 is similar to those of meropenem (16 μg mL^–1^) and ciprofloxacin (4 μg mL^–1^) (Table S6), which are clinically used.

**Table 4:** Minimal Inhibitory Concentration (MIC) in μg mL^–1^of **D22** and **D69** in an Extended Panel of Clinically Relevant Gram-Negative Pathogens

	**MIC[μg mL**^**–1**^**]**	
**bacterial strains**	**D22**	**D69**
*Acinetobacter baumannii* DSM-30007	2–4	2
*A. baumannii* DSM-30008	0.25–0.5	0.5
*A. baumannii* NCTC 13301	4–8	8–16
*A. baumannii* ATCC 17978	4–8	16
*Pseudomonas aeruginosa* PA01 DSM 22644	0.25	1
*P. aeruginosa* PA14 DSM 19882	2–4	16
*P. aeruginosa*83979^a^	4–8	8–16
*P. aeruginosa* 84389^a^	16	16–32

a MDR clinical isolates (see the SI); *n* = 2.

## Discussion and Conclusions

Changes in the darobactin core peptide significantly influence antibacterial activity of the analogues positively or negatively.^12,15^ Thus, we used existing structural data of antibiotic darobactins **DA**, **D9**, and **D22** as a guide to study the influence of scaffold modifications at underinvestigated amino acid positions. We started by simulating targeted mutagenesis of the cryo-EM costructure of BAM–**D22**^14,15^ to predict potential influences of amino acid exchanges in the darobactin heptapeptide for the formation of hydrogen bonding interactions. Indeed, the modeling predicted strong interactions at position 2 for **D69**, no interaction for **D64**, and even no direct overall changes for **D39** after modifying the respective positions compared to **D22**. To create the corresponding bioactivity readouts, we used our genetic modification and extraction platform to produce the new analogues. Through the fermentation process, we could obtain all engineered novel darobactins in titers clearly higher than those of previously published native derivatives **DD** and **DE** that share modifications at positions 2 and 5.^23^ The production of darobactins, however, still differs significantly between derivatives. This might be attributed to the intrinsic sensitivity of the heterologous host *E. coli*, but further factors could also reduce production. For example, the modifications, especially in positions 2 and 5, could prevent the proper bicyclization of darobactins by steric hindrance of the radical SAM DarE or impair the still unknown peptidase responsible for the release of the mature heptapeptide on the C- and N-terminal ends of the core peptide due to the high target specificity in RiPPs.^26,27^ Nevertheless, the heterologous production of darobactins in *E. coli* is robust, and certain derivatives like **D74** (which has only one change at position 6 compared to **D22**) exhibit higher production rates than all known artificial analogues. This could be due to its lower activity against *E. coli*; yet, this observation was not made for **D60**. Thus, there might be other reasons for varying the production titers. In addition to the potentially better interference with DarE, this derivative might be transferred out of the bacterial cell in a more efficient process, which remains to be characterized. Nonetheless, the approximately 3-fold increase in production compared to **D22** makes **D74** an interesting candidate for potential semisynthetic approaches. To achieve further and less restricted modifications of biosynthetically generated darobactins, semisynthesis can become an alternative to total synthesis, which is currently costly and time-consuming.^28,29^ Combinations of fermentation and semisynthetic approaches might help in the future to find a path forward for large-scale production of darobactins in amounts high enough for preclinical and clinical development of this new class of antibiotics at costs acceptable for pharmaceutical development.^30,31^

Importantly, our biosynthetic approach enables access to a structurally diverse set of darobactins for further investigation. We were able to study modified analogues substituted with amino acids at positions that have not been tested before. Consequently, we could enhance the repertoire of artificial darobactins and our knowledge about the influence of respective modifications especially at positions 2 and 5 but also in combination with position 4. In contrast to previous reports, we showed that the change at position 2, as in **DE**, does not necessarily lead to strongly reduced antibacterial activity compared to **DA**.^23^ The strain-specific antibacterial activity is in line with the findings that already single amino acid substitutions in the compound-binding site are able to completely abolish activity, which has been shown, *e.g.*, for the non-native derivative **D25** but also for BamA mutants.^11,23^ Although causative point mutations in the respective pathogens could easily lead to resistance, it has already been shown that such modifications are connected to severe fitness loss of the mutants.^32^ Interestingly, similar differences between native and artificial analogues, as seen for **DE** and **D64** at position 2, were observed for modification at position 5. The artificial darobactin **D39** (WNWTRRW), bearing an l-arginine instead of l-lysine at position 5 as in native **DD** (WNWSRSF), was active as a pure compound, whereas pure **DD** was published to be inactive against Gram-negative bacteria.^23^ The positive charge of the arginine side chain should also interact with the phosphate moieties of cardiolipin and 1-palmitoyl-2-oleoyl-*sn*-glycero-3-phosphoglycerol, which was shown for l-lysine by Kaur et al.^14^ Thus, we hypothesized that **DD** should have a comparable antibacterial activity to **DA**. Differences between **DD** and **D39,** however, can also be due to other positional changes (Table 2). Thus, we reproduced **DD** purification and antibacterial characterization to gain certainty about the bioactivity of **DD**. The production and purification process of **DD** in the previously published paper significantly differs from our methods,^15,23^ which led to considerably higher yields. Notably, the pure **DD** was highly active in our MIC assays, validated by *K*_D_ determination via MST. In general, an MIC shift between derivatives might be explained by the slight orientation shift of darobactins binding to BamA due to the change at position 4 from l-serine to l-threonine^15^ and due to the terminal switch from l-phenylalanine to l-tryptophan. Especially, the change of the terminal amino acid was proven to enhance the antibacterial activity of **D9** up to 8-fold compared to **DA**.^12^ The binding constants now determined via MST validate MIC data of pure analogues for *Ec*BamA. By using MST for the first time to determine binding to BamA, we significantly reduced the required amount of protein and ligand per assay compared to ITC, which turned out advantageous due to the reduced production yields for these derivatives. The profiling of new analogues regarding cytotoxicity against HepG2 cells did not show any toxic effects that could be caused by an interaction with eukaryotic membrane β-barrels. Further ADMET profiling data reveal high metabolic and plasma stability and low PPB. This is in line with the high polarity of the compound class and hints at a dominance of renal excretion over hepatic metabolism, which must be confirmed *in vivo*.

In conclusion, the two frontrunners **D22** and **D69** display properties attractive enough to be further evaluated *in vivo* (Table 3). Potential future steps include determination of pharmacokinetic properties followed by studies in *in vivo* infection models focusing on *E. coli* but also *P. aeruginosa* or *A. baumannii*. The new derivatives extend the repertoire of darobactins with validated antibacterial activity data for the best characterized molecules, even against clinical isolates of multidrug-resistant *A. baumannii* and *P. aeruginosa* strains. Further derivatization of darobactins has thus expanded our current knowledge of the darobactin–BAM interaction and its impact on biological activity against difficult-to-treat Gram-negative pathogens.

## Experimental Section

### General Procedures

The *E. coli* HS996 strain was used as a bacterial cloning strain for the transformation of ligation mixtures with digested, modified *darA* gene fragments and the pNOSO-darABCDE-22^15^ backbone to construct novel BGC. The modified darA gene fragments were generated with the overlap extension PCR method, as described previously.^15^ The cultivation of the bacterial cloning strain was performed as described previously.^12,15^ In brief, the cultivation of *E. coli* HS996 was performed in LB medium (10 g/L tryptone, 5 g/L NaCl, and 5 g/L yeast extract, pH 7.0) at 30 °C. The production host *E. coli* BL21 (DE3) was grown in FM medium (12.54 g/L K_2_HPO_4_, 2.31 g/L KH_2_PO_4_, 5 g/L NaCl, 12 g/L yeast extract, 4 g/L d(+)-glucose, 1 g/L NH_4_Cl, and 0.24 g/L MgSO_4_·7H_2_O, pH 7.1) including 1 mg/L sterile filtered vitamin B12. The producer strains, previously transformed with expression vectors (*e.g.*, pNOSO-darABCDE-58 to 74), were separately incubated for 16 h at 30 °C in LB medium. Seeded overnight broth (0.5 mL) was used to inoculate 50 mL of FM medium. The production cultures were shaken at 30 °C for 3 days with an appropriate selection marker.^12,15^

### Analysis of the Production of Novel Darobactins

The detection and verification of new artificial darobactins produced by overexpression of the mutated *darA* variants were verified using the analytical tools described by Groß et al.^12^ In brief, the new analogues in the fermentation broth were measured by analytic ultrahigh-performance liquid chromatography-high-resolution mass spectrometry (UHPLC-HRMS) using *m*/*z* values of [M + 2H]^2+^, [M + 3H]^3+^, and [M + 3H-NH_3_]^3+^ and MS^2^ fragmentation analysis of [M + 2H]^2+^ (Figures S21–S37) as calculated (Table S5). To obtain comparable data, multiple ionization states were recorded and combined in an extracted ion chromatogram (EIC) because the amino acid exchange, *e.g.*, of basic amino acids, shifted the most abundant ion species from doubly charged [M + 2H]^2+^ to triply charged states ([M + 3H]^3+^ and [M + 3H-NH_3_]^3+^. The integrated combined EIC value of each derivative, detectable in the extracted samples, was computed by automated peak integration using Compass Data Analysis version 5.3 (Bruker Daltonics) and divided through the area under the curve (AUC) of **D22** (Table 1).

### UHPLC-HRMS Analysis

All analyses, except for the MS^2^ spectra and quantification of production for **DD**, **D58**, and **D61**, were carried out as described by Groß et al.^12^ The fermentation broth of 50 mL screening cultures was centrifuged at 7750*g* for 15 min at 4 °C. The supernatant was directly analyzed by UHPLC-HRMS using an UltiMate 3000 LC system (Dionex, Sunnyvale, California (US)), which was coupled to either maXis 4G ToF, timsTOF fleX, or amaZon speed mass spectrometers (Bruker Daltonics). LC conditions and couplings were as follows: An Acquity UPLC BEH C18 column (1.7 μm, 100 mm × 2 mm; Waters Corporation, Milford, Massachusetts (US)), equipped with a VanGuard BEH C18 (1.7 μm; Waters Corp.) guard column, was coupled to an Apollo II ESI source (Bruker Daltonics; Billerica, Massachusetts (US)) and hyphenated to respective mass spectrometers. Separation was performed at a flow rate of 0.6 mL/min (eluent A: deionized H_2_O + 0.1% formic acid (FA), eluent B: acetonitrile + 0.1% FA) at 45 °C using the following gradient: 5% B for 30 s followed by a linear gradient up to 95% B in 18 min and a constant percentage of 95% B for a further 2 min. Original conditions were adjusted with 5% B within 30 s and kept constant for 1.5 min. The LC flow was split to 75 μL/min before the mass spectrometer. In the case of the maXis 4G and timsTOF flex, each run started with a calibrant peak of basic sodium formate solution, which was provided by a filled 20 μL loop switched into the LC flow at the beginning of each run.

Parameters for the maXis 4G were as follows: Mass spectra were acquired in the centroid mode ranging from 150 to 2500 *m*/*z* at a 2 Hz full scan rate. Mass spectrometry source parameters were set to a 500 V end plate offset, a 4000 V capillary voltage, a 1 bar nebulizer gas pressure, a 5 L/min dry gas flow, and a 200 °C dry temperature. For MS^2^ experiments, CID (collision-induced dissociation) energy was ramped from 35 eV for 500 *m*/*z* to 45 eV for 1,000 *m*/*z*. The MS full scan acquisition rate was set to 2 Hz, and MS^2^ spectra acquisition rates were ramped from 1 to 4 Hz for precursor ion intensities of 10 to 1,000 kcts. If analyses on the maXis 4G did not result in clear spectra, a timsTOF fleX was used instead: For MS^2^ experiments, the same LC system, column, and eluents and gradient were used but coupled to a timsTOF fleX mass spectrometer (Bruker Daltonics) with the same ESI source and source conditions. MS^2^ spectra were acquired using the parallel acquisition and serial fragmentation (PASEF) mode under the following conditions: TIMS delta values were set to −20 (delta 1), −120 (delta 2), 80 (delta 3), 100 (delta 4), 0 (delta 5), and 100 V (delta 6). The 1/*K*_0_ (inverse reduced ion mobility) range was set from 0.55 to 1.9 V s/cm^2^, and the mass range was *m*/*z* 100–2000. MS^2^ spectra were acquired using the PASEF DDA mode with a collision energy of 30 eV. Ion charge control (ICC) was enabled and set to 7.5 Mio. counts. The analysis accumulation and ramp time were set at 100 ms with a spectra rate of 9.43 Hz, and a total cycle of 0.32 s was also selected resulting in one full TIMS-MS scan and two PASEF MS/MS scans. Precursor ions were actively excluded for 0.1 min and were reconsidered if the intensity was 2.0-fold higher than the previous selection with a target intensity of 4000 and an intensity threshold of 100. The TIMS dimension was calibrated linearly using 4 selected ions from an ESI low-concentration tuning mix (Agilent Technologies, USA) [*m*/*z*, 1/*k*_0_: (301.998139, 0.6678 V s cm^–2^), (601.979077, 0.8782 V s cm^–2^)] in the negative mode and [*m*/*z*, 1/*k*_0_: (322.048121, 0.7363 V s cm^–2^), (622.028960, 0.9915 V s cm^–2^)] in the positive mode. The mobility for mobility calibration was taken from the CCS compendium.^33^

Some quality controls were run on the amaZon speed in the positive ionization mode. MS settings were as follows: a capillary voltage of 4500 V, an end plate offset of 500 V, a nebulizer of 30.00 psi, a dry gas flow of 10 L/min, and a dry gas temperature of 300 °C. The scan range for standard measurements was 200–2000 *m*/*z* with a target mass at 600 *m*/*z*.

**DD**, **D58**, and **D61** production was quantified using a Vanquish Flex UHPLC (Thermo Fisher, Dreieich, Germany), coupled to a TSQ Altis Plus mass spectrometer (Thermo Fisher, Dreieich, Germany). Supernatants were diluted to 1:10 in PBS pH 7.4 followed by addition of 2 volumes of 10% MeOH/ACN containing 15 nM diphenhydramine as an internal standard. Samples were centrifuged (15 min, 4 °C, 4000 rpm) before analysis, and the darobactin content was quantified in the SRM mode using a calibration curve. LC conditions were as follows: column, Hypersil GOLD C18 (1.9 μm, 100 × 2.1 mm; Thermo Fisher, Dreieich, Germany); temperature, 40 °C; flow rate, 0.700 mL/min; solvent A, deionized H_2_O + 0.1% FA; solvent B, acetonitrile + 0.1% FA; gradient, 0–0.2 min 10% B, 0.2–1.2 min 10–90% B, 1.2–1.6 min 90% B, 1.6–2.0 min 10% B. MS conditions were as follows: vaporizer temperature, 350 °C; ion transfer tube temperature, 380 °C; sheath gas, 30; aux gas, 10; sweep gas, 2; spray voltage, 3700 (**DD**) and 3300 V (**D61** and **D58**); mass transitions, 497.750–489.083 (**DD**, [M + 2H]^2+^), 552.167–543.667 (**D61**, [M + 2H]^2+^), and 552.300–543.883 (**D58**, [M + 2H]^2+^); collision energy, 10.9 (**DD**), 13.1 (**D61**), and 9.3 V (**D58**); tube lens offset, 59 (**DD**), 89 (**D61**), and 96 V (**D58**).

### Fermentation and Purification of Darobactins

All pure darobactins were characterized by a purity of over 95%.

Fermentation of novel darobactin derivatives was achieved in 5 L shaking flasks each containing 1.5 L of FM medium supplemented with 30 μg/mL kanamycin as a selection marker.

A total of 6 × 1.5 L of main cultures were inoculated with 15 mL of a well-grown overnight culture of an *E. coli* BL21 (DE3) producer strain harboring the modified darobactin BGC, which was cultivated for 16 h at 30 °C and 180 rpm in LB medium with an appropriate selection marker (30 μg/mL kanamycin). The main production cultures in 5 L shaking flasks were incubated for 3 days at 30 °C and 160 rpm on an orbital shaker.

After incubation, the 1.5 L production broth was centrifuged at 6000*g* for 15 min at 4 °C to remove the bacterial cells from the supernatant. The collected supernatant was pH adjusted to 7.0–7.3 with NaOH or HCl and mixed with 2% (w/V) cation exchange resin Dowex MAC-3 for 5–6 h at 4 °C and 400 rpm on an orbital shaker. The extraction of the novel darobactins was performed as described in more detail previously.^15^ In brief, the resin was washed twice with H_2_O_dest_ for about 15 min and incubated overnight at 4 °C in H_2_O_dest_. The novel darobactins were then eluted from the resin 5–7 times with 300 mL each of a 2 M ammonia solution for 30 min, where the supernatant was decanted after each elution step. The eluate containing the darobactins was then neutralized on ice with 99% (V/V) acetic acid until a pH between 4 and 7 was reached. Afterward, the eluate was filtered using folded filter paper.

Purification of the darobactin-containing supernatant was performed by two to three chromatographic steps, depending on purity. First, fractionation was performed by a combination of solid-phase extraction and flash chromatography using a 130 g C18 flash column (CHROMABOND flash RS 120 C18 ec, 40–63 μm). The eluate-loaded C18 column was first washed with 2 column volumes (CV) of H_2_O_dest_ to remove salts and highly polar compounds. Fractionation was then performed with a gradient using a mobile phase composed of deionized H_2_O + 0.1% FA (eluent A) and acetonitrile (ACN) + 0.1% FA (eluent B) on a Biotage flash chromatographic system (Isolera One) at a flow rate of 50 mL/min under the following conditions.

One CV of H_2_O_dest_ without FA was performed followed by elution with 20 CV of 5% B increased to 25% B followed by a ramp of 2 CV to 95% B and 2 CV of 95% eluent B as a cleaning step. Detection was performed with 220 and 280 nm UV absorption. The darobactin-containing fractions were collected and concentrated by using a rotary evaporator.

In a second chromatographic step, the darobactin-containing fractions were purified by preparative reversed-phase chromatography on a preparative Autopurifier HPLC-MS system by Waters Corp. using an XBridge C18 column (5 μm, 19 mm × 150 mm; Waters Corp.). Separation was performed using the same solvents (eluent A and eluent B) as previously described for the first purification step at a flow rate of 25 mL/min with the following gradients.

HPLC conditions for purification of **D61** and **D64** were initially an equilibration with 5% eluent B and 95% eluent A for 2 min followed by a gradient from 5 to 20% B for 22 min, a ramp to 95% B for another 2 min, and constant holding at 95% B for 1 min. The initial conditions were set within 2 min ramping back to 5% B. The elution of **D61** occurred after 13.5 min and for **D64** after 14.5 min.

**D58** and **D74** and native **DD** were separated with the following gradient: an equilibration step with 2% B and 98% A for 2 min followed by a linear gradient to 25% B over 22 min, an increase to 95% B within 2 min, keeping at 95% B for 1 min, and ramping back to the initial 2% B within 2 min. The elution of **D58** occurred at minute 11.4, for **D74** at minute 12.5, and for **DD** at minute 13.5.

Purification of **D60** was initially achieved with equilibration at 10% B and 90% A for 2 min, a linear separation gradient from 10% B up to 17% B for 22 min, and an increase to 95% B within 2 min and holding at 95% B for 1 min. The initial conditions were set by ramping back to 10% B within 2 min. The elution of **D60** occurred after 12 min.

**D69** and **D39** were separated using the following HPLC conditions: an equilibration step at 2% B and 98% A for the initial 2 min followed by a linear gradient from 2% B up to 15% B for 22 min, an increase up to 95% B within 2 min, and keeping 95% B for 1 min. Afterward, the initial conditions of 2% B and 98% A were set within 2 min. The elution of **D69** occurred after 17 min and of **D39** after 15 min. The fractions containing darobactin were collected according to their elution times and concentrated using a rotary evaporator.

For some derivatives, a further purification step was performed on an UltiMate 3000 semipreparative HPLC system (Thermo Scientific) using an Acquity CSH phenyl-hexyl column (250 mm × 10 mm, 5 μm; Waters Corp.) to obtain entirely pure compounds. Separation was performed using the same solvents (eluent A and eluent B) as previously described for the first and second purification step at a flow rate of 5 mL/min and a column temperature of 45 °C. Detection of darobactin was performed by UV absorption at 280 nm, and the corresponding fractions were collected in a time-dependent manner. The following gradients were used.

Purification of **D58** and **D60** was achieved using an initial equilibration step of 2% eluent B and 98% eluent A for 2 min followed by a linear gradient from 2 to 25% B for 22 min, an increase up to 95% B within 2 min, and holding at 95% B for 1 min followed by setting the initial conditions of 2% B within 2 min. The elution of **D57** occurred at minute 12.1, of **D58** at minute 9.5, and of **D60** at minute 10.

Separation of **D64** was also started with an equilibration step of 2% B for 2 min. The HPLC conditions were then a gradient from 2 to 15% B for 22 min and an increase to 95% B within 2 min, holding 95% B for 1 min, and adjusting the initial conditions by ramping back to 2% B within 2 min. Elution of **D64** occurred after 10.8 min. The corresponding fractions containing darobactin were collected, concentrated, and dried using a rotary evaporator, and purity was confirmed by UHPLC-HRMS analysis.

### Determination of Antibacterial Activity

The antibacterial activity of novel darobactins was determined by the evaluation of the minimum inhibitory concentration (MIC) as previously described for crude extracts and for pure darobactin analogues.^12^

### Microscale Thermophoresis Assay

Microscale thermophoresis (MST) (serial no. 201709-BR-N024, Monolith NT.115 Micro Scale Thermophoresis, NanoTemper Technologies GmbH) was performed according to the standard protocol from the manufacturer NanoTemper Technologies GmbH using a Monolith His-Tag Labeling Kit RED-tris-NTA second-generation kit. The buffer used was HEPES (50 mM), pH 7.6, MgCl_2_ (5 mM), and Tween (0.05%). The protein concentration of 50 nM was used, and the ligand was tested at the highest soluble concentration, which was 2 mM for most of the compounds under the assay conditions. A 1:1 dilution of the ligand over 16 samples was performed using a stock of ligand (in water) diluted in HEPES buffer. Nonhydrophobic capillary tubes were used. A pretest to check for the labeling and compound fluorescence was performed for every sample followed by a binding affinity (*K*_D_) determination. Each sample was measured after 15 min of incubation at RT and analyzed in MO Control version 1.6.

### ADMET Profiling of **D22** and **D69**

#### Metabolic Stability in Liver Microsomes

For the evaluation of phase I metabolic stability, the compound (1 μM) was incubated with 0.5 mg/mL pooled C57BL/6 mouse, Wistar rat liver microsomes (Xenotech, Kansas City, USA), or human liver microsomes (Corning, New York, USA), 2 mM NADPH, and 10 mM MgCl_2_ at 37 °C for 120 min on a microplate shaker (Eppendorf, Hamburg, Germany). The metabolic stability of testosterone, verapamil, and ketoconazole was determined in parallel to confirm the enzymatic activity of mouse/rat liver microsomes; for human liver microsomes, testosterone, diclofenac, and propranolol were used. Incubation was stopped after defined time points by precipitation of aliquots of enzymes with 2 volumes of cold acetonitrile containing an internal standard (15 nM diphenhydramine). Samples were stored on ice until the end of the incubation, and precipitated protein was removed by centrifugation (15 min, 4 °C, and 4000*g*). The concentration of the remaining test compound at the different time points was analyzed by HPLC-MS/MS (TSQ Altis Plus, Thermo Fisher, Dreieich, Germany) and used to determine the half-life (*t*_1/2_).

#### Stability in Plasma

To determine stability in plasma, the compound (1 μM) was incubated with pooled CD-1 mouse, Wistar rat, or human plasma (Neo Biotech, Nanterre, France). Samples were taken at defined time points by mixing aliquots with 4 volumes of acetonitrile containing an internal standard (12.5 nM diphenhydramine). Samples were stored on ice until the end of the incubation, and precipitated protein was removed by centrifugation (15 min, 4 °C, 4000*g*, and 2 centrifugation steps). The concentration of the remaining test compound at the different time points was analyzed by HPLC-MS/MS (TSQ Quantum Access MAX, Thermo Fisher, Dreieich, Germany). The plasma stability of procaine, propantheline, and diltiazem was determined in parallel to confirm the enzymatic activity.

#### Plasma Protein Binding

Plasma protein binding was determined using a Rapid Equilibrium Dialysis (RED) system (Thermo Fisher Scientific, Waltham MA, USA). Compounds were diluted in murine (CD-1), rat (Wistar), or human plasma (Neo Biotech, Nanterre, France) to 10 μM and added to the respective chamber according to the manufacturer’s protocol followed by addition of PBS pH 7.4 to the opposite chamber. Samples were taken immediately after addition to the plate as well as after 2, 4, and 5 h by mixing 10 μL with 80 μL of ice-cold acetonitrile containing 12.5 nM diphenhydramine as an internal standard followed by addition of 10 μL of plasma to samples taken from PBS and vice versa. Samples were stored on ice until the end of the incubation, and precipitated protein was removed by centrifugation (15 min, 4 °C, 4000*g*, and 2 centrifugation steps). The concentration of the remaining test compound at the different time points was analyzed by HPLC-MS/MS (TSQ Altis Plus, Thermo Fisher, Dreieich, Germany). The amount of the compound bound to protein was calculated using the equation PPB [%] = 100 – 100 × (amount in the buffer chamber/amount in the plasma chamber).

#### Protein Expression and Purification

The *E. coli* BamA barrel domain (BamA-β) (residues 421–810, C690S, C700S), with an N-terminal 6× His-tag was overexpressed and purified as described previously.^15^

#### NMR Spectroscopy of **D69**

NMR data were recorded on an UltraShield 500 MHz (^1^H at 500 MHz, ^13^C at 125 MHz) equipped with a 5 mm inverse TCI cryoprobe (Bruker, Billerica, MA, USA). Shift values (δ) were calculated in ppm, and coupling constants (*J*) were calculated in Hz. For the two-dimensional experiments, HMBC, HSQC, and gCOSY standard pulse programs were used. HMBC experiments were optimized for ^2,3^*J*_C–H_ = 6 Hz, and HSQC ones were optimized for ^1^*J*_C__–__H_ = 145 Hz. NMR data of darobactin **69** showed high similarity to those of **D22** when comparing 1D and 2D NMR spectra. However, instead of the l-asparagine and l-threonine, a glutamine moiety and a l-serine moiety could be identified, respectively, as evidenced by typical proton and carbon shifts as well as COSY and HMBC correlations (Table S7). The amino acid sequence of **D69** was predetermined by the core peptide and confirmed by HMBC correlations from α-protons to carbonyl carbons. A couple of amino acid connections that could not be established by HMBC NMR data due to missing correlations were confirmed based on ESI-HR-MS^2^ analysis (Figure S39).

## Supporting Information Available

Additional figures and tables including modeled BamA–darobactin interactions, MS and MS^2^ fragmentation patterns of all derivatives, chromatograms of extracts, antibiogram of clinical *P. aeruginosa* isolates, quantification of pure darobactins, NMR spectroscopic data of **D69**, and HPLC traces of pure compounds (PDF)
Molecular formula strings (CSV)
Model of BamA–**D39** (PDB)
Model of BamA–**D58** (PDB)
Model of BamA–**D60** (PDB)
Model of BamA–**D61** (PDB)
Model of BamA–**D64** (PDB)
Model of BamA–**D67** (PDB)
Model of BamA–**D69**_v1 (PDB)
Model of BamA–**D69**_v2 (PDB)
Model of BamA–**D74** (PDB)

## Supplementary Material

jm3c01660_si_002.csv

jm3c01660_si_003.pdb

jm3c01660_si_004.pdb

jm3c01660_si_005.pdb

jm3c01660_si_006.pdb

jm3c01660_si_007.pdb

jm3c01660_si_008.pdb

jm3c01660_si_009.pdb

jm3c01660_si_010.pdb

jm3c01660_si_011.pdb

## ABBREVIATIONS

BAM: BamABCDE complex
BGC: biosynthetic gene cluster
Cl_int_: intrinsic clearance
CRAB: carbapenem-resistant *A. baumannii*
cryo-EM: cryogenic electron microscopy
EcBamA: *E. coli* BamA
FM: formulated medium
ITC: isothermal titration calorimetry
*K*_D_: dissociation constant
MIC: minimal inhibitory concentration
MoB: mode of binding
MST: microscale thermophoresis
OMP: outer membrane protein
PPB: plasma protein binding
RiPPs: ribosomally synthesized and post-translationally modified peptides
rSAM: radical *S*-adenosyl-l-methionine
*t*_1/2_: half-life time
