# Supplementary material for: New Genetically Engineered Derivatives of Antibacterial Darobactins Underpin Their Potential for Antibiotic Development
Source: J Med Chem. 2023 Nov 21;66(23):16330–41. doi: 10.1021/acs.jmedchem.3c01660 (PMC10726357; doi:10.1021/acs.jmedchem.3c01660)
Supplement: Supplementary file 1 — jm3c01660_si_001.pdf [file jm3c01660_si_001.pdf]

# Supporting Information

# New Genetically Engineered Derivatives of Antibacterial Darobactins Underpin their Potential for Antibiotic Development

Carsten E. Seyfert[a,b], Alison V. Müller[a,b], Danica J. Walsh[a,b], Joy Birkelbach[a,b], Andreas M. Kany[a,b], Christoph Porten[a,b], Biao Yuan[c,d,f], Daniel Krug[a,b], Jennifer Herrmann[a,b], Thomas C. Marlovits[c,d,f], Anna K. H. Hirsch[a,b,e] and Rolf Müller*[a,b,e]

[a]Helmholtz Institute for Pharmaceutical Research Saarland (HIPS), Helmholtz Centre for Infection Research (HZI) and Saarland University Department of Pharmacy, 66123 Saarbrücken, Germany

[b]German Centre for Infection Research (DZIF), partner site 38124 Hannover-Braunschweig, Germany

[c]University Medical Center Hamburg-Eppendorf (UKE), Institute of Structural and Systems Biology and Centre for Structural Systems Biology (CSSB), 22607 Hamburg, Germany

[d]Deutsches Elektronen-Synchrotron Zentrum (DESY), 22607 Hamburg, Germany

[e]Helmholtz International Lab for Anti-Infectives, 66123 Saarbrücken, Germany

[f]Centre for Structural Systems Biology (CSSB), 22607 Hamburg, Germany

**Corresponding Author**

*E.mail: rolf.mueller@helmholtz-hips.de. Phone: (+49)681 98806-3002

Table of Contents

Table of figures .......... S2

List of tables .......... S3

Structure-driven *in silico* design of new darobactin analogues .......... S4

Supplementation figures and tables .......... S9

1. Mutagenesis of the darobactin heptapeptide .......... S9

2. Chromatograms of darobactin extracts .......... S11

3. $MS^2$ spectra analysis of darobactin analogues .......... S18

4. Antibiogram of clinical *P. aeruginosa* isolates .......... S27

5. Quantification of production .......... S27

6. NMR spectroscopic data .......... S28

7. HPLC traces of pure compounds .......... S35

References .......... S38

Table of figures

Figure S 1: Mutagenesis of **D22** bound to BamABCDE to predict the possible interaction of **D39**, **D58**, **D60**, **D61**, **D64** and **D67** with BamA. .......... S9
Figure S 2: Mutagenesis of **D22** bound to BamABCDE to predict the possible interaction of D69 and D74 with BamA. .......... S10
Figure S 3: Chromatogram of the *E. coli* BL21 (DE3) pNOSO-darABCDE-58 extract. .......... S12
Figure S 4: Chromatogram of the *E. coli* BL21 (DE3) pNOSO-darABCDE-59 extract. .......... S12
Figure S 5: Chromatogram of the *E. coli* BL21 (DE3) pNOSO-darABCDE-60 extract. .......... S12
Figure S 6: Chromatogram of the *E. coli* BL21 (DE3) pNOSO-darABCDE-61 extract. .......... S13
Figure S 7: Chromatogram of the *E. coli* BL21 (DE3) pNOSO-darABCDE-62 extract. .......... S13
Figure S 8: Chromatogram of the *E. coli* BL21 (DE3) pNOSO-darABCDE-63 extract. .......... S13
Figure S 9: Chromatogram of the *E. coli* BL21 (DE3) pNOSO-darABCDE-64 extract. .......... S14
Figure S 10: Chromatogram of the *E. coli* BL21 (DE3) pNOSO-darABCDE-65 extract. .......... S14
Figure S 11: Chromatogram of the *E. coli* BL21 (DE3) pNOSO-darABCDE-66 extract. .......... S14
Figure S 12: Chromatogram of the *E. coli* BL21 (DE3) pNOSO-darABCDE-67 extract. .......... S15
Figure S 13: Chromatogram of the *E. coli* BL21 (DE3) pNOSO-darABCDE-68 extract. .......... S15
Figure S 14: Chromatogram of the *E. coli* BL21 (DE3) pNOSO-darABCDE-69 extract. .......... S15
Figure S 15: Chromatogram of the *E. coli* BL21 (DE3) pNOSO-darABCDE-70 extract. .......... S16
Figure S 16: Chromatogram of the *E. coli* BL21 (DE3) pNOSO-darABCDE-71 extract. .......... S16
Figure S 17: Chromatogram of the *E. coli* BL21 (DE3) pNOSO-darABCDE-72 extract. .......... S16
Figure S 18: Chromatogram of the *E. coli* BL21 (DE3) pNOSO-darABCDE-73 extract. .......... S17
Figure S 19: Chromatogram of the *E. coli* BL21 (DE3) pNOSO-darABCDE-74 extract. .......... S17
Figure S 20: $MS^2$ fragmentation pattern of darobactin analogues. .......... S18
Figure S 21: $MS^2$ spectrum for **D58** .......... S20
Figure S 22: $MS^2$ spectrum for **D59** .......... S20
Figure S 23: $MS^2$ spectrum for **D60** .......... S20
Figure S 24: $MS^2$ spectrum for **D61** .......... S21
Figure S 25: $MS^2$ spectrum for **D62** .......... S21
Figure S 26: $MS^2$ spectrum for **D63** .......... S21
Figure S 27: $MS^2$ spectrum for **D64** .......... S22
Figure S 28: $MS^2$ spectrum for **D65** .......... S22
Figure S 29: $MS^2$ spectrum for **D66** .......... S22
Figure S 30: $MS^2$ spectrum for **D67** .......... S23
Figure S 31: $MS^2$ spectrum for **D68** .......... S23
Figure S 32: $MS^2$ spectrum for **D69** .......... S23
Figure S 33: $MS^2$ spectrum for **D70** .......... S24
Figure S 34: $MS^2$ spectrum for **D71** .......... S24
Figure S 35: $MS^2$ spectrum for **D72** .......... S24
Figure S 36: $MS^2$ spectrum for **D73** .......... S25

Figure S 37: $MS^2$ spectrum for **D74**. S25
Figure S 38: Overview of all generated novel darobactin analogues. S26
Figure S 39: Production yield of darobactin **D22**, **D69** and **D74**. S27
Figure S 40: $MS^2$ spectrum of **D69**. S29
Figure S 41: $^1$H NMR of **D69** recorded in $D_2O$/acetonitrile-$d_3$ (2:1) + 1% formic acid-$d_4$ at 318 K. S30
Figure S 42: $^{13}$C NMR of **D69** recorded in $D_2O$/acetonitrile-$d_3$ (2:1) + 1% formic acid-$d_4$ at 318 K. S31
Figure S 43: HSQC NMR of **D69** recorded in $D_2O$/acetonitrile-$d_3$ (2:1) + 1% formic acid-$d_4$ at 318 K. S32
Figure S 44: COSY NMR of **D69** recorded in $D_2O$/acetonitrile-$d_3$ (2:1) + 1% formic acid-$d_4$ at 318 K. S33
Figure S 45: HMBC NMR of **D69** recorded in $D_2O$/acetonitrile-$d_3$ (2:1) + 1% formic acid-$d_4$ at 318 K. S34
Figure S 46: Chromatogram of purified **DD**. S35
Figure S 47: Chromatogram of purified **D22**. S35
Figure S 48: Chromatogram of purified **D39**. S35
Figure S 49: Chromatogram of purified **D58**. S36
Figure S 50: Chromatogram of purified **D60**. S36
Figure S 51: Chromatogram of purified **D61**. S36
Figure S 52: Chromatogram of purified **D64**. S37
Figure S 53: Chromatogram of purified **D69**. S37
Figure S 54: Chromatogram of purified **D74**. S37

List of tables

Table S 1: Oligonucleotides used to mutate the *darA* core region for the generation of novel expression constructs with modified darobactin BGC. S4
Table S 2: Expression plasmids generated to express and produce novel darobactins in *E. coli* BL21 (DE3). S5
Table S 3: List of bacterial strains generated or used in this study. S6
Table S 4: The calculated (calc.) and observed (obs.) masses of the darobactin analogues. S11
Table S 5: Calculated (calc.) and observed (obs.) masses of the typical b2 and y5 fragment ions. S19
Table S 6: Antibiogram of clinical *P. aeruginosa* isolates. Antibacterial activity of **D22** and **D69** S27
Table S 7: NMR Spectroscopic Data for **D69**[a] S28

Structure-driven *in silico* design of new darobactin analogues

BamABCDE (BAM) – darobactin cryo-EM co-structures with the accession code 7NRI (**DA**), 8ADI (**D9**) and 8ADG (**D22**) were analyzed on their appropriate interaction site using Pymol 2.5.2 and ChimeraX1.3 software 1; 2. The Pymol "Mutagenesis" and the Chimera "rotamers" tool were used to exchange the asparagine on position 2 of the darobactin heptapeptide with proteinogenic amino acids of variable length and polarity.[1–3] The potential hydrogen bonding interactions were automatically computed in the respective structural analysis software.[2,4] The orientation of the changed amino acids was calculated by the automatic rotamer tool based on the probabilities of the rotamer library. Based on the findings, novel derivatives, potentially interacting with BamA, were designed and generated using the overlap-extension PCR method previously developed to generated darobactin **D22** to **D39**.[5] In brief, oligonucleotides harboring mismatches compared to the nucleotide core sequence of pNOSOdarABCDE-D22 were externally synthesized (Sigma-Aldrich) (Table S1). In two independent PCR reactions (PCR I, PCR II) fragments, harboring point mutations in the nucleotide core region, were amplified and used as templates for an overlapping PCR (PCR III) to combine the fragments of PCR I and PCR II. The combined fragments were separated via agarose gel electrophoresis, and purified using the NucleoSpin Gel and PCR Cleanup kit (Macherey-Nagel). The purified DNA fragments were digested using restriction-ligation based techniques to generate novel expression constructs summarized in Table S2.

**Table S 1: Oligonucleotides used to mutate the *darA* core region for the generation of novel expression constructs with modified darobactin BGC.**

| Oligonucleotide name | Sequence (5'-3') | Function | Reference |
|---|---|---|---|
| pr1_dar9_fw | GGTGATGTCGGCGATA TAGG | Primer for PCR I and III | Seyfert *et al.* 2022 |
| pr1_dar9_v2_fw | GGTGATGTCGGCGATA TAGGCG | Primer for PCR I and III | Seyfert *et al.* 2022 |
| pr2_dar22_58b_rv | GCCACCGTTTAGTCCA TTCCCAGGCCGT | Primer for PCR I to insert point mutation into *darA* | This study |
| pr2_dar22_59b_rv | GCCACCGTTTAGTCCA TTTCCAGGCCGT | Primer for PCR I to insert point mutation into *darA* | This study |
| pr2_dar22_60_rv | GCCACCGTTTAGTCCA ATCCCAGGCCGT | Primer for PCR I to insert point mutation into *darA* | This study |
| pr2_dar22_61_rv | GCCACCGTTTAGTCCA TTGCCAGGCCGT | Primer for PCR I to insert point mutation into *darA* | This study |
| pr2_dar22_62_rv | GCCACCGTTTAGTCCA TCTCCAGGCCGT | Primer for PCR I to insert point mutation into *darA* | This study |
| pr2_dar22_63_rv | GCCACCGTTTAGTCCA AACCCAGGCCGT | Primer for PCR I to insert point mutation into *darA* | This study |
| pr2_dar22_64_rv | GCCACCGTTTAGTCCA TGACCAGGCCGT | Primer for PCR I to insert point mutation into *darA* | This study |
| pr2_dar22_65_rv | GCCATTTTTTAGTCCAT GACCAGGCCGT | Primer for PCR I to insert point mutation into *darA* | This study |
| pr2_dar22_65_rv2 | GCCATTTTTTAGTCCAT GACCAGG | Primer for PCR I to insert point mutation into *darA* | This study |
| pr2_dar22_66_rv | AAATTTCCTGCCAACA TTTAGTCCAGTTCCA | Primer for PCR I to insert point mutation into *darA* | This study |
| pr2_dar22_67_rv | GCCACCGTTTAGTCCA ATACCAGGCCGT | Primer for PCR I to insert point mutation into *darA* | This study |
| pr2_dar22_68_rv | GCCACCGTTTAGTCCA TAACCAGGCCGT | Primer for PCR I to insert point mutation into *darA* | This study |
| pr2_dar22_69_rv | GCCACCGTTTTGACCA TTGCCAGGCCGT | Primer for PCR I to insert point mutation into *darA* | This study |

| | | | |
|---|---|---|---|
| pr2_dar22_70_rv | GCCACCGTTTTGACCA TGACCAGGCCGT | Primer for PCR I to insert point mutation into *darA* | This study |
| pr2_dar22_71_rv | GCCACCGTTTTGACCA TTCCCAGGCCGT | Primer for PCR I to insert point mutation into *darA* | This study |
| pr2_dar22_72_rv | GCCACCGTTTTGACCA TTTCCAGGCCGT | Primer for PCR I to insert point mutation into *darA* | This study |
| pr2_dar22_72_rv2 | GCCACCGTTTTGACCA TTTCCAGGC | Primer for PCR I to insert point mutation into *darA* | This study |
| pr2_dar22_73_rv | GCCACCGTTTTGACCA ATCCCAGGCCGT | Primer for PCR I to insert point mutation into *darA* | This study |
| pr2_dar22_74_rv | AAATTTCCTGCCATTG TTTAGTCCAGTTCCA | Primer for PCR I to insert point mutation into *darA* | This study |
| pr3_dar9_Trp_fw | TGGCAGGAAATTTAAA GCTTATCCCAT | Primer for PCR II | Seyfert *et al.* 2022 |
| pr3_dar22_v2_fw | CTAAACGGTGGCAGGA AATTTAAAGCTTATC | Primer for PCR II | Seyfert *et al.* 2022 |
| pr3_dar65b_6K_fw | CTAAAAAATGGCAGGA AATTTAAAGCTTATCC CA | Primer for PCR II | This study |
| pr3_dar22_6C_fw | CTAAATGTTGGCAGGA AATTTAAAGCTTATC | Primer for PCR II | This study |
| pr3_dar22_4S_fw | CAAAACGGTGGCAGG AAATTTAAAGCTTATC | Primer for PCR II | This study |
| pr3_dar22_6Q_fw | CTAAACAATGGCAGGA AATTTAAAGCTTATC | Primer for PCR II | This study |
| pr4_dar9_rv | TGGGGATCCTCAGGAC TGCAG | Primer for PCR II and III | Seyfert *et al.* 2022 |

**Table S 2: Expression plasmids generated to express and produce novel darobactins in *E. coli* BL21 (DE3).**

| **Plasmid** | **size [kb]** | **Relevant characteristics/ construction** | **Reference** |
|---|---|---|---|
| pNOSO-darABCDE-22 | 9.9 | *E. coli* cloning and expression vector for production of **D22** | Seyfert *et al.* |
| pNOSO-darABCDE-58 | 9.9 | *E. coli* cloning and expression vector for production of **D58** | This study |
| pNOSO-darABCDE-59 | 9.9 | *E. coli* cloning and expression vector for production of **D59** | This study |
| pNOSO-darABCDE-60 | 9.9 | *E. coli* cloning and expression vector for production of **D60** | This study |
| pNOSO-darABCDE-61 | 9.9 | *E. coli* cloning and expression vector for production of **D61** | This study |
| pNOSO-darABCDE-62 | 9.9 | *E. coli* cloning and expression vector for production of **D62** | This study |
| pNOSO-darABCDE-63 | 9.9 | *E. coli* cloning and expression vector for production of **D63** | This study |
| pNOSO-darABCDE-64 | 9.9 | *E. coli* cloning and expression vector for production of **D64** | This study |
| pNOSO-darABCDE-65 | 9.9 | *E. coli* cloning and expression vector for production of **D65** | This study |
| pNOSO-darABCDE-66 | 9.9 | *E. coli* cloning and expression vector for production of **D66** | This study |
| pNOSO-darABCDE-67 | 9.9 | *E. coli* cloning and expression vector for production of **D67** | This study |
| pNOSO-darABCDE-68 | 9.9 | *E. coli* cloning and expression vector for production of **D68** | This study |

| | | | |
|---|---|---|---|
| pNOSO-darABCDE-69 | 9.9 | *E. coli* cloning and expression vector for production of **D69** | This study |
| pNOSO-darABCDE-70 | 9.9 | *E. coli* cloning and expression vector for production of **D70** | This study |
| pNOSO-darABCDE-71 | 9.9 | *E. coli* cloning and expression vector for production of **D71** | This study |
| pNOSO-darABCDE-72 | 9.9 | *E. coli* cloning and expression vector for production of **D72** | This study |
| pNOSO-darABCDE-73 | 9.9 | *E. coli* cloning and expression vector for production of **D73** | This study |
| pNOSO-darABCDE-74 | 9.9 | *E. coli* cloning and expression vector for production of **D74** | This study |

**Table S 3: List of bacterial strains generated or used in this study.**

| Bacterial strain | Genotype | Reference |
|---|---|---|
| Cloning strains | | |
| *E. coli* HS996 | F$^-$, *mcr*A, Δ(*mrr-hsd*RMS-*mcr*BC), Φ80*lac*ZΔM15, Δ*lac*X74, *rec*A1, *ara*D139, Δ(*ara-leu*)7697, *gal*U, *gal*K, *rps*L (Str$^R$), *end*A1, *nup*G, *fhu*A::IS2 | Invitrogen |
| *E. coli* NEB10β | *mcr*A, spoT1Δ(*mrr-hsd*RMS-*mcr*BC), Φ80d(*lac*ZΔM15)*rec*A1, *rel*A1, Δ*lac*X74, *rec*A1, *ara*D139, Δ(*ara-leu*)7697, *gal*K16, *gal*E15, *rps*L (Str$^R$), *end*A1, *nup*G, *fhu*A | New England Biolabs |
| *E. coli* BL21 (DE3) | F$^–$, *ompT, gal, dcm, lon,* Δ$hsdS_B$($r_B^–m_B^–$), λ(DE3 [*lacI lacUV5-T7p07 ind1 sam7 nin5*]), [*malB*$^+$]$_{K-12}$(λ$^S$) | Invitrogen |
| *E. coli* BL21-Gold (DE3) | *E. coli* B F– ompT hsdS(rB– mB–) dcm+ Tetr gal λ(DE3) endA | Agilent Technologies |
| *E. coli* NEB10β pNOSO-darABCDE-22 | *E. coli* NEB10β with pNOSO-darABCDE-22, kan$^R$ | Seyfert *et al.* 2022 |
| *E. coli* HS996 pNOSO-darABCDE-58 | *E. coli* HS996 with pNOSO-darABCDE-58, kan$^R$ | This study |
| *E. coli* HS996 pNOSO-darABCDE-59 | *E. coli* HS996 with pNOSO-darABCDE-59, kan$^R$ | This study |
| *E. coli* HS996 pNOSO-darABCDE-60 | *E. coli* HS996 with pNOSO-darABCDE-60, kan$^R$ | This study |
| *E. coli* HS996 pNOSO-darABCDE-61 | *E. coli* HS996 with pNOSO-darABCDE-61, kan$^R$ | This study |
| *E. coli* HS996 pNOSO-darABCDE-62 | *E. coli* HS996 with pNOSO-darABCDE-62, kan$^R$ | This study |
| *E. coli* HS996 pNOSO-darABCDE-63 | *E. coli* HS996 with pNOSO-darABCDE-63, kan$^R$ | This study |
| *E. coli* HS996 pNOSO-darABCDE-64 | *E. coli* HS996 with pNOSO-darABCDE-64, kan$^R$ | This study |
| *E. coli* HS996 pNOSO-darABCDE-65 | *E. coli* HS996 with pNOSO-darABCDE-65, kan$^R$ | This study |
| *E. coli* HS996 pNOSO-darABCDE-66 | *E. coli* HS996 with pNOSO-darABCDE-66, kan$^R$ | This study |
| *E. coli* HS996 pNOSO-darABCDE-67 | *E. coli* HS996 with pNOSO-darABCDE-67, kan$^R$ | This study |

| | | |
|---|---|---|
| *E. coli* HS996 pNOSO-darABCDE-68 | *E. coli* HS996 with pNOSO-darABCDE-68, $kan^R$ | This study |
| *E. coli* HS996 pNOSO-darABCDE-69 | *E. coli* HS996 with pNOSO-darABCDE-69, $kan^R$ | This study |
| *E. coli* HS996 pNOSO-darABCDE-70 | *E. coli* HS996 with pNOSO-darABCDE-70, $kan^R$ | This study |
| *E. coli* HS996 pNOSO-darABCDE-71 | *E. coli* HS996 with pNOSO-darABCDE-71, $kan^R$ | This study |
| *E. coli* HS996 pNOSO-darABCDE-72 | *E. coli* HS996 with pNOSO-darABCDE-72, $kan^R$ | This study |
| *E. coli* HS996 pNOSO-darABCDE-73 | *E. coli* HS996 with pNOSO-darABCDE-73, $kan^R$ | This study |
| *E. coli* HS996 pNOSO-darABCDE-74 | *E. coli* HS996 with pNOSO-darABCDE-74, $kan^R$ | This study |
| Heterologous producer strains | | |
| *E. coli* BL21 (DE3) pNOSO-darABCDE-22 | *E. coli* BL21 (DE3) with pNOSO-darABCDE-22, $kan^R$ | This study |
| *E. coli* BL21-Gold (DE3) pNOSO-darABCDE-22 | *E. coli* BL21-Gold (DE3) with pNOSO-darABCDE-22, $kan^R$ | This study |
| *E. coli* BL21 (DE3) pNOSO-darABCDE-58 | *E. coli* BL21 (DE3) with pNOSO-darABCDE-58, $kan^R$ | This study |
| *E. coli* BL21 (DE3) pNOSO-darABCDE-59 | *E. coli* BL21 (DE3) with pNOSO-darABCDE-59, $kan^R$ | This study |
| *E. coli* BL21 (DE3) pNOSO-darABCDE-60 | *E. coli* BL21 (DE3) with pNOSO-darABCDE-60, $kan^R$ | This study |
| *E. coli* BL21 (DE3) pNOSO-darABCDE-61 | *E. coli* BL21 (DE3) with pNOSO-darABCDE-61, $kan^R$ | This study |
| *E. coli* BL21 (DE3) pNOSO-darABCDE-62 | *E. coli* BL21 (DE3) with pNOSO-darABCDE-62, $kan^R$ | This study |
| *E. coli* BL21 (DE3) pNOSO-darABCDE-63 | *E. coli* BL21 (DE3) with pNOSO-darABCDE-63, $kan^R$ | This study |
| *E. coli* BL21 (DE3) pNOSO-darABCDE-64 | *E. coli* BL21 (DE3) with pNOSO-darABCDE-64, $kan^R$ | This study |
| *E. coli* BL21 (DE3) pNOSO-darABCDE-65 | *E. coli* BL21 (DE3) with pNOSO-darABCDE-65, $kan^R$ | This study |
| *E. coli* BL21 (DE3) pNOSO-darABCDE-66 | *E. coli* BL21 (DE3) with pNOSO-darABCDE-66, $kan^R$ | This study |
| *E. coli* BL21 (DE3) pNOSO-darABCDE-67 | *E. coli* BL21 (DE3) with pNOSO-darABCDE-67, $kan^R$ | This study |
| *E. coli* BL21 (DE3) pNOSO-darABCDE-68 | *E. coli* BL21 (DE3) with pNOSO-darABCDE-68, $kan^R$ | This study |
| *E. coli* BL21 (DE3) pNOSO-darABCDE-69 | *E. coli* BL21 (DE3) with pNOSO-darABCDE-69, $kan^R$ | This study |
| *E. coli* BL21 (DE3) pNOSO-darABCDE-70 | *E. coli* BL21 (DE3) with pNOSO-darABCDE-70, $kan^R$ | This study |
| *E. coli* BL21 (DE3) pNOSO-darABCDE-71 | *E. coli* BL21 (DE3) with pNOSO-darABCDE-71, $kan^R$ | This study |
| *E. coli* BL21 (DE3) pNOSO-darABCDE-72 | *E. coli* BL21 (DE3) with pNOSO-darABCDE-72, $kan^R$ | This study |
| *E. coli* BL21 (DE3) pNOSO-darABCDE-73 | *E. coli* BL21 (DE3) with pNOSO-darABCDE-73, $kan^R$ | This study |
| *E. coli* BL21 (DE3) pNOSO-darABCDE-74 | *E. coli* BL21 (DE3) with pNOSO-darABCDE-74, $kan^R$ | This study |

| | | |
|---|---|---|
| *E. coli* BL21 (λ DE3) Lemo cells pET15b-BamAβ421-810 | *E. coli* BL21 (λ DE3) Lemo cells with pET15b-BamAβ421-810, amp$^R$ | Seyfert *et al.* 2022 |

Supplementation figures and tables

1. Mutagenesis of the darobactin heptapeptide

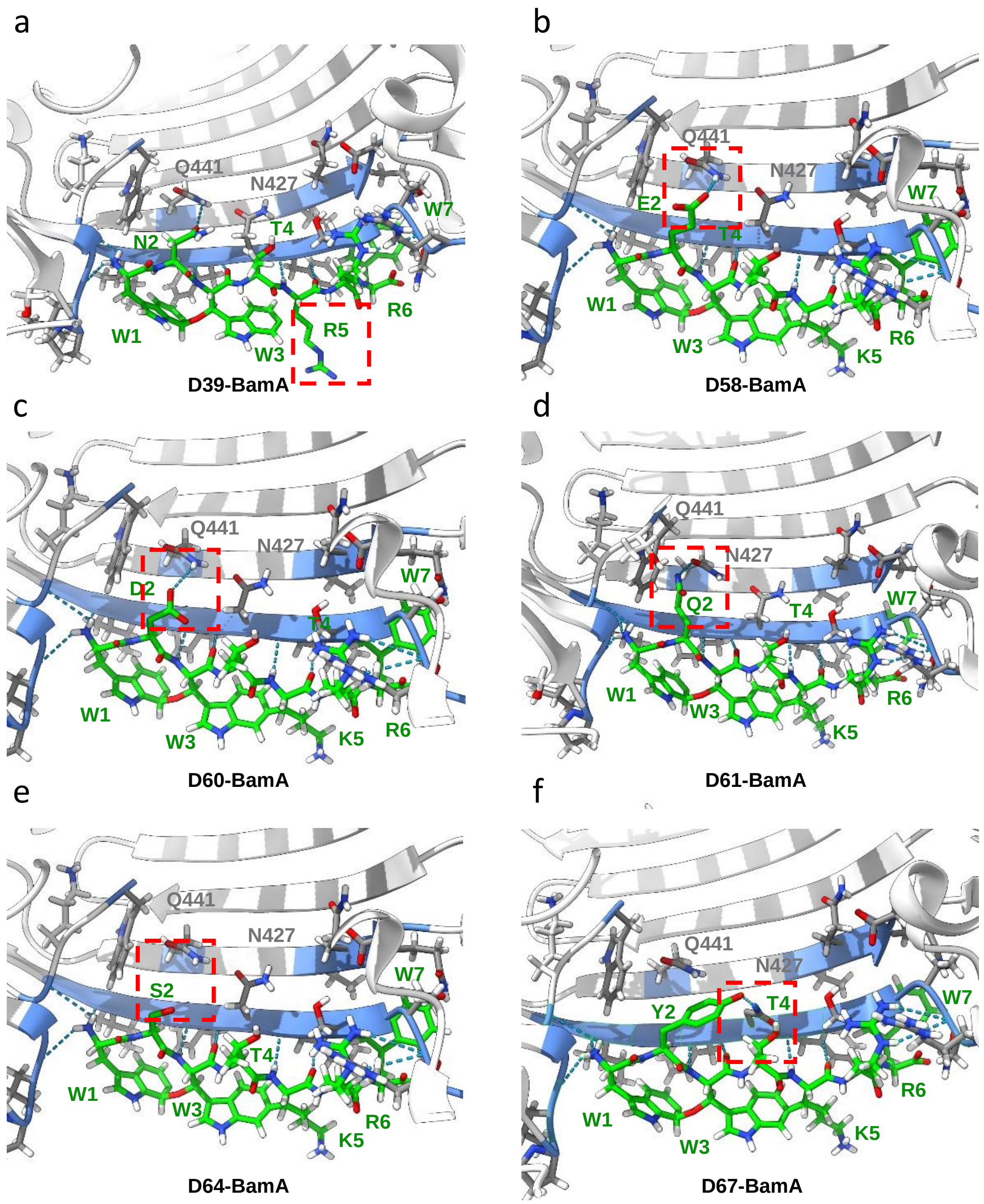


**Figure S 1: Mutagenesis of D22 bound to BamABCDE to predict the possible interaction of D39, D58, D60, D61, D64 and D67 with BamA**. Chimera rotamer tool was used to model potential hydrogen bonding interactions by changing a) L-arginine to L- lysine to project **D38**-BamA interaction b) L-asparagine to L-glutamic acid to project **D58**-BamA interaction c) L- asparagine to L-aspartic acid to project **D60**-BamA interaction d) L-asparagine to L-glutamine to project **D61**-BamA interaction e) L-asparagine to L-serine to project **D64**-BamA interaction f) L-asparagine to L-tyrosine to project **D67**-BamA interaction. Changes and modeled interactions were highlighted in red. The raw data of **D22** were taken from protein data bank (PDB) for the mutagenesis and were originally published in Seyfert *et al*.[5] with the PDB accession code: 8ADG (D22).

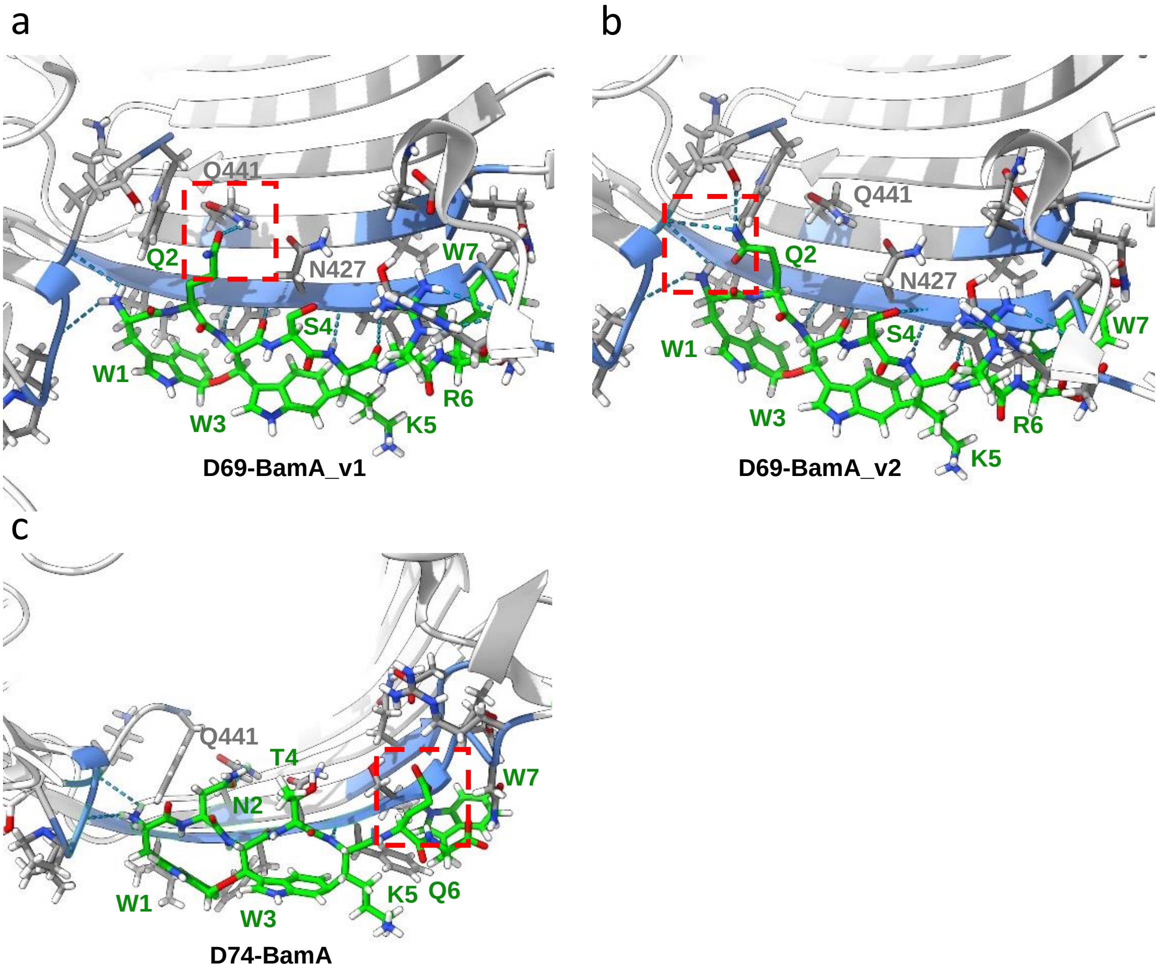


**Figure S 2: Mutagenesis of D22 bound to BamABCDE to predict the possible interaction of D69 and D74 with BamA**. Chimera rotamer tool was used to model the interactions by changing of a) and b) L-asparagine to L-glutamine to project **D69**-BamA interaction c) L-arginine to L-glutamine to project **D74**-BamA interaction. Changes and modeled interactions were highlighted in red. The raw data of **D22** were taken from protein data bank (PDB) as starting point for molecular modeling. The accession code is 8ADG (**D22**).

2. Chromatograms of darobactin extracts

**Table S 4: The calculated (calc.) and observed (obs.) masses of the darobactin analogues.** The core peptide sequence of each new derivative with highlighted (red) differences in the core peptide compared to **D22** is indicated. The calc. and obs. masses in the extracts of the different charge states ($[M+H]^{1+}$, $[M+2H]^{2+}$ and $[M+3H]^{3+}$) for each derivative are listed.

| Darobactin analogue | Core peptide | Calc. mass $[M+H]^{1+}$ | Obs. mass $[M+H]^{1+}$ | Calc. mass $[M+2H]^{2+}$ | Obs. mass $[M+2H]^{2+}$ | Calc. mass $[M+3H]^{3+}$ | Obs. mass $[M+3H]^{3+}$ | Calc. mass $[M+3H-NH_3]^{3+}$ | Obs. mass $[M+3H-NH_3]^{3+}$ |
|---|---|---|---|---|---|---|---|---|---|
| D58 | W E W T K R W | 1103.5058 | 1103.5171 | 552.2565 | 552.2632 | 368.5068 | 368.5109 | 362.8318 | 362.8351 |
| D59 | W K W T K R W | 1102.5582 | 1102.4853 | 551.7827 | 551.7889 | 368.1909 | 368.1951 | 362.5159 | 362.5204 |
| D60 | W D W T K R W | 1089.4901 | 1089.5022 | 545.2487 | 545.2555 | 363.8349 | 363.8392 | 358.1599 | 358.1636 |
| D61 | W Q W T K R W | 1102.5218 | 1102.5318 | 551.7645 | 551.7711 | 368.1788 | 368.1831 | 362.5038 | 362.5073 |
| D62 | W R W T K R W | 1130.5643 | 1130.5152 | 565.7858 | 565.7906 | 377.5263 | 377.5306 | 371.8513 | 371.8691 |
| D63 | W V W T K R W | 1073.5316 | 1073.5133 | 537.2694 | 537.2752 | 358.5154 | 358.1436 | 352.8404 | 352.8433 |
| D64 | W S W T K R W | 1061.4952 | 1061.5028 | 531.2512 | 531.2575 | 354.5033 | 354.5071 | 348.8283 | 348.8315 |
| D65 | W S W T K K W | 1033.4891 | 1033.4981 | 517.2482 | 517.2535 | 345.1679 | 345.1713 | 339.4929 | 339.4956 |
| D66 | W N W T K C W | 1226.4394 | 1226.4533 | 613.7207 | 613.7307 | 409.4896 | 409.4887 | 403.8146 | 403.8122 |
| D67 | W Y W T K R W | 1137.5265 | 1137.5359 | 569.2669 | 569.2734 | 379.8470 | 379.8510 | 374.1720 | 374.1754 |
| D68 | W L W T K R W | 1087.5473 | 1087.5103 | 544.2773 | 544.2830 | 363.1873 | 363.1909 | 357.5123 | 357.5151 |
| D69 | W Q W S K R W | 1088.5061 | 1088.5114 | 544.7567 | 544.7603 | 363.5069 | 363.5092 | 357.8319 | 357.8334 |
| D70 | W S W S K R W | 1047.4796 | 1047.4822 | 524.2434 | 524.2461 | 349.8314 | 349.8328 | 344.1564 | 344.1574 |
| D71 | W E W S K R W | 1089.4901 | 1089.4893 | 545.2487 | 545.2491 | 363.8349 | 363.8350 | 358.1599 | 358.1593 |
| D72 | W K W S K R W | 1088.5425 | 1088.4476 | 544.7749 | 544.7741 | 363.5190 | 363.5184 | 357.8440 | 357.8430 |
| D73 | W D W S K R W | 1075.4745 | 1075.4735 | 538.2409 | 538.2413 | 359.1630 | 359.1631 | 353.4880 | 353.4876 |
| D74 | W N W T K Q W | 1060.4636 | 1060.4622 | 530.7354 | 530.7372 | 354.1594 | 354.1588 | 348.4844 | 348.4836 |

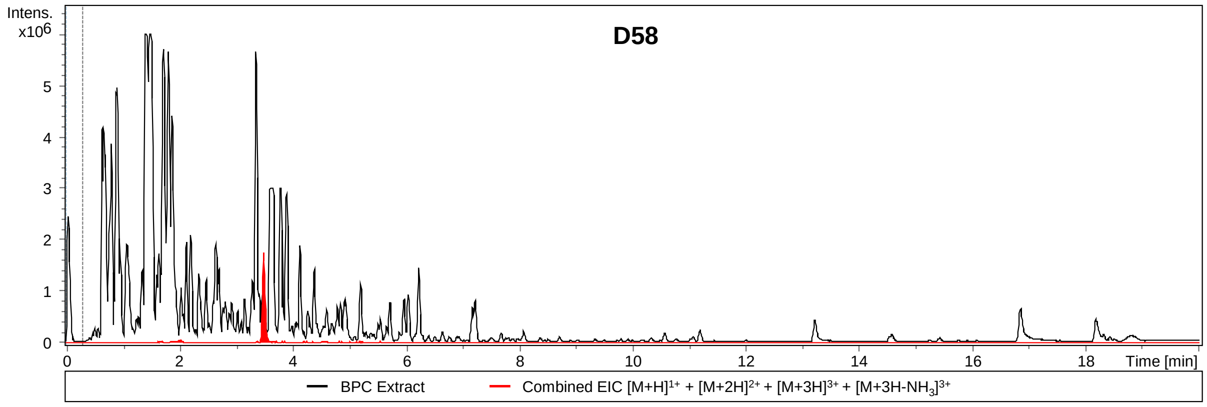


**Figure S 3: Chromatogram of the *E. coli* BL21 (DE3) pNOSO-darABCDE-58 extract**. The red trace shows the combined EIC for the $[M+H]^{1+}$, $[M+2H]^{2+}$, $[M+3H]^{3+}$ and $[M+3H-NH_3]^{3+}$ species of **D58** at their calculated masses, respectively (**Table S 4**) ±0.02 Da. The BPC of the whole extract from fermentation broth supernatant is presented in black.

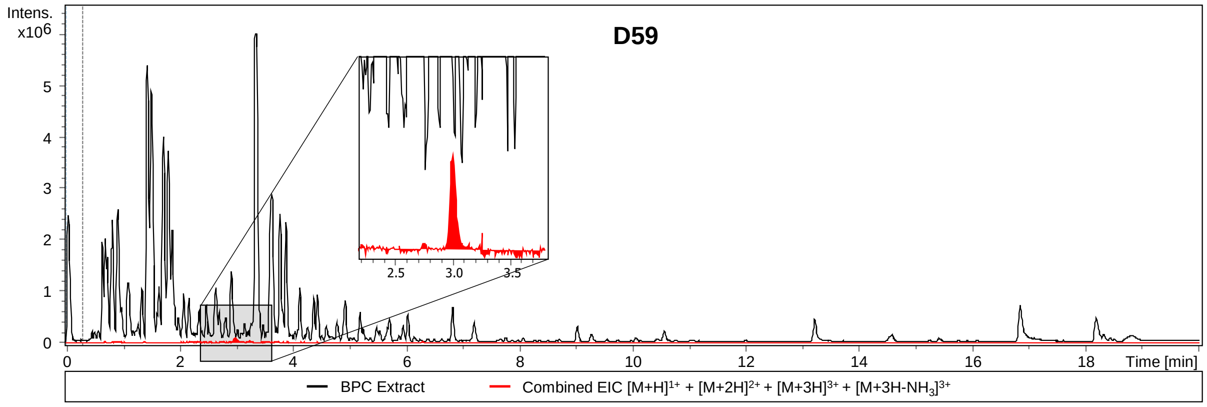


**Figure S 4: Chromatogram of the *E. coli* BL21 (DE3) pNOSO-darABCDE-59 extract**. The red trace shows the combined EIC for the $[M+H]^{1+}$, $[M+2H]^{2+}$, $[M+3H]^{3+}$ and $[M+3H-NH_3]^{3+}$ species of **D59** at their calculated masses, respectively (**Table S 4**) ±0.02 Da. The BPC of the whole extract from fermentation broth supernatant is presented in black.

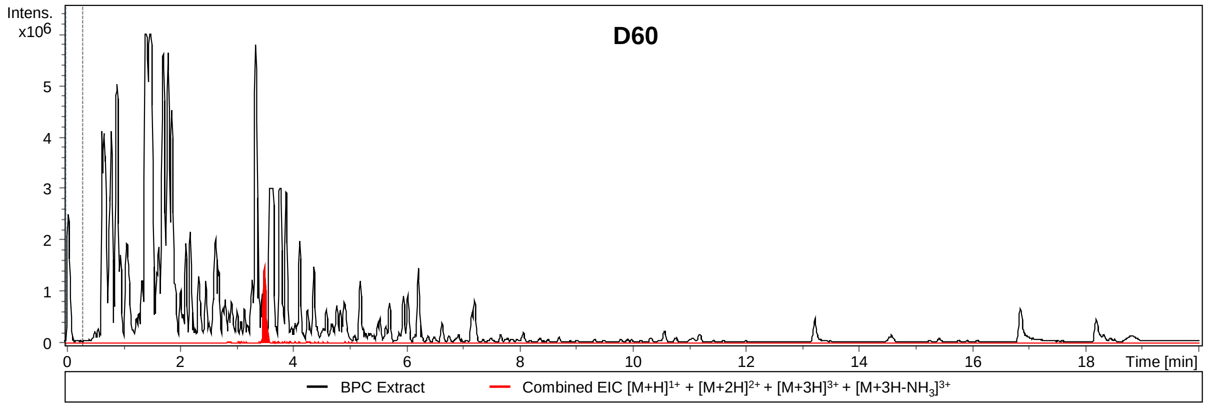


**Figure S 5: Chromatogram of the *E. coli* BL21 (DE3) pNOSO-darABCDE-60 extract**. The red trace shows the combined EIC for the $[M+H]^{1+}$, $[M+2H]^{2+}$, $[M+3H]^{3+}$ and $[M+3H-NH_3]^{3+}$ species of **D60** at their calculated masses, respectively (**Table S 4**) ±0.02 Da. The BPC of the whole extract from fermentation broth supernatant is presented in black.

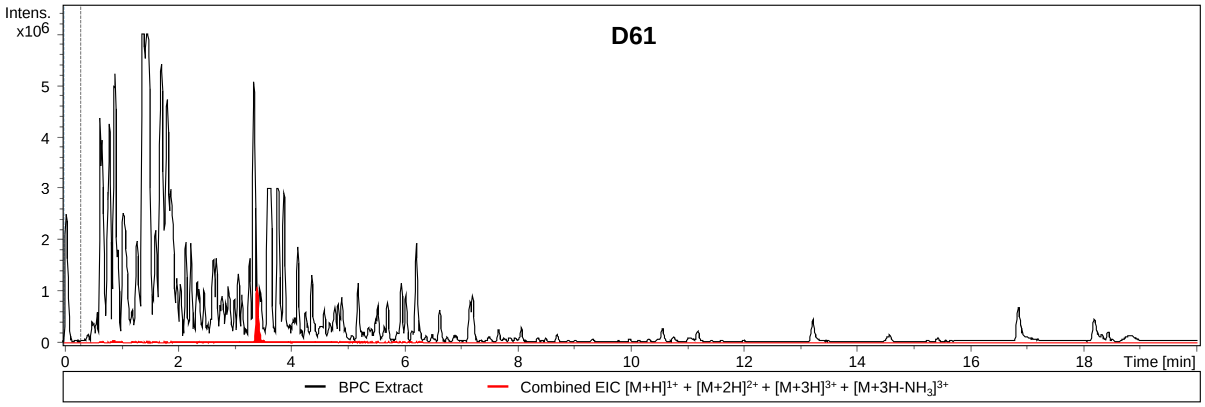


**Figure S 6: Chromatogram of the *E. coli* BL21 (DE3) pNOSO-darABCDE-61 extract**. The red trace shows the combined EIC for the $[M+H]^{1+}$, $[M+2H]^{2+}$, $[M+3H]^{3+}$ and $[M+3H-NH_3]^{3+}$ species of **D61** at their calculated masses, respectively (**Table S 4**) ±0.02 Da. The BPC of the whole extract from fermentation broth supernatant is presented in black.

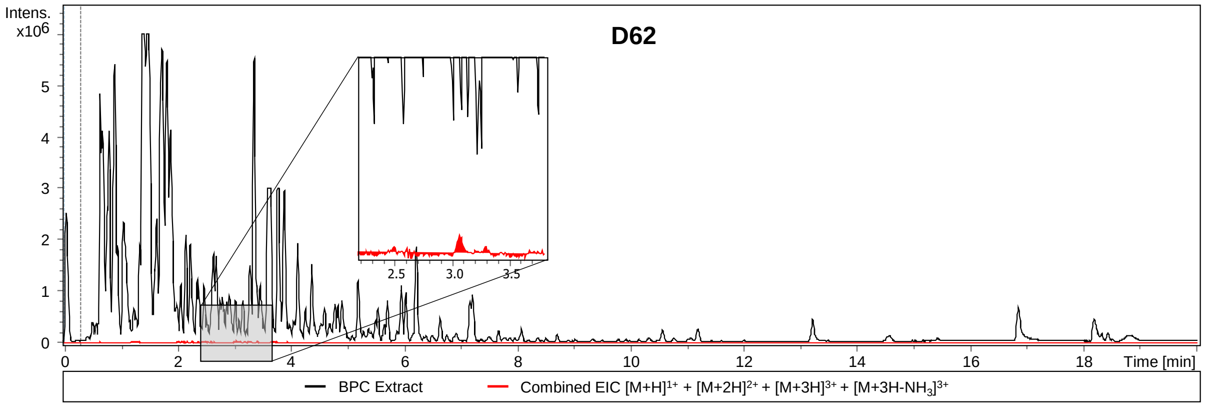


**Figure S 7: Chromatogram of the *E. coli* BL21 (DE3) pNOSO-darABCDE-62 extract**. The red trace shows the combined EIC for the $[M+H]^{1+}$, $[M+2H]^{2+}$, $[M+3H]^{3+}$ and $[M+3H-NH_3]^{3+}$ species of **D62** at their calculated masses, respectively (**Table S 4**) ±0.02 Da. The BPC of the whole extract from fermentation broth supernatant is presented in black.

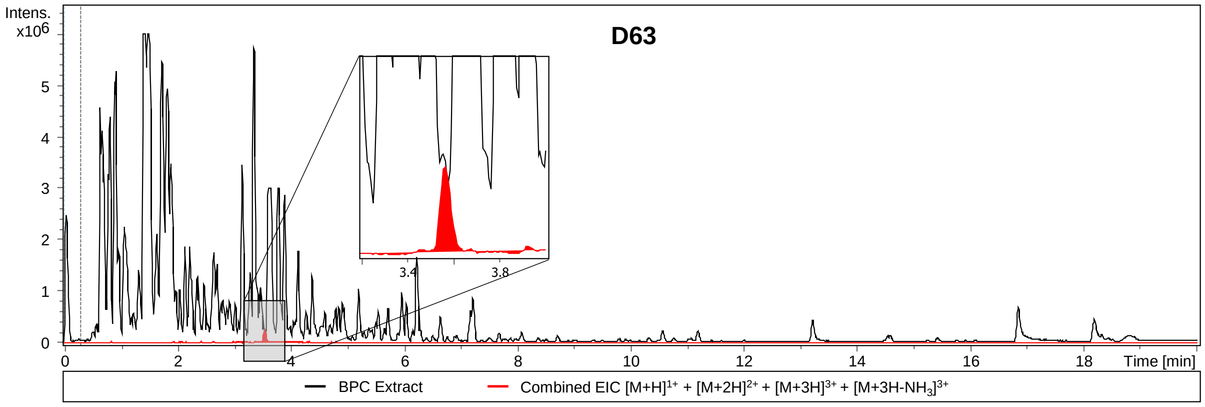


**Figure S 8: Chromatogram of the *E. coli* BL21 (DE3) pNOSO-darABCDE-63 extract**. The red trace shows the combined EIC for the $[M+H]^{1+}$, $[M+2H]^{2+}$, $[M+3H]^{3+}$ and $[M+3H-NH_3]^{3+}$ species of **D63** at their calculated masses, respectively (**Table S 4**) ±0.02 Da. The BPC of the whole extract from fermentation broth supernatant is presented in black.

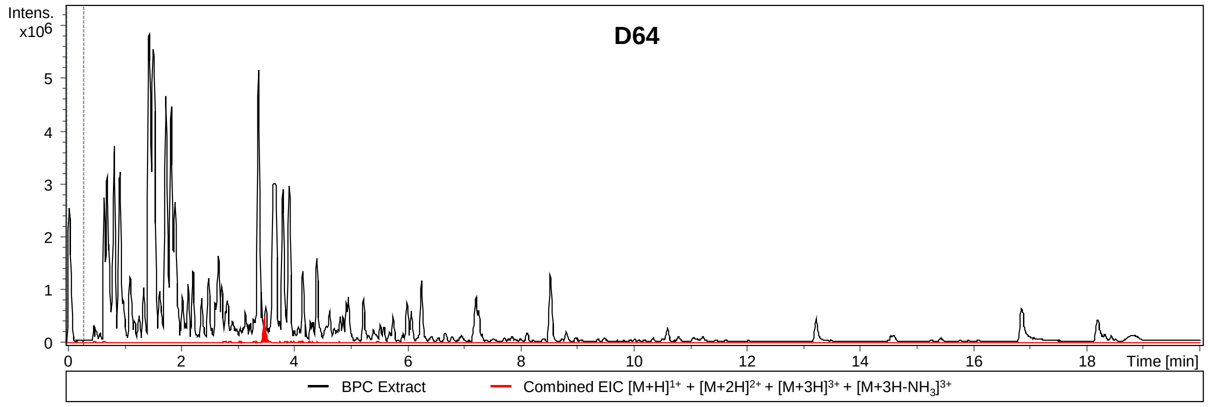


**Figure S 9: Chromatogram of the *E. coli* BL21 (DE3) pNOSO-darABCDE-64 extract**. The red trace shows the combined EIC for the $[M+H]^{1+}$, $[M+2H]^{2+}$, $[M+3H]^{3+}$ and $[M+3H\text{-}NH_3]^{3+}$ species of **D64** at their calculated masses, respectively (**Table S 4**) ±0.02 Da. The BPC of the whole extract from fermentation broth supernatant is presented in black.

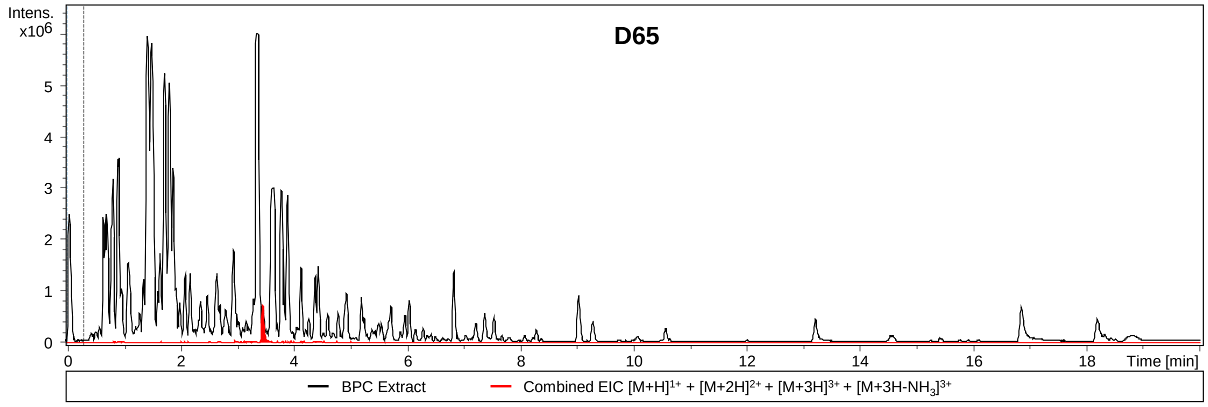


**Figure S 10: Chromatogram of the *E. coli* BL21 (DE3) pNOSO-darABCDE-65 extract**. The red trace shows the combined EIC for the $[M+H]^{1+}$, $[M+2H]^{2+}$, $[M+3H]^{3+}$ and $[M+3H\text{-}NH_3]^{3+}$ species of **D65** at their calculated masses, respectively (**Table S 4**) ±0.02 Da. The BPC of the whole extract from fermentation broth supernatant is presented in black.

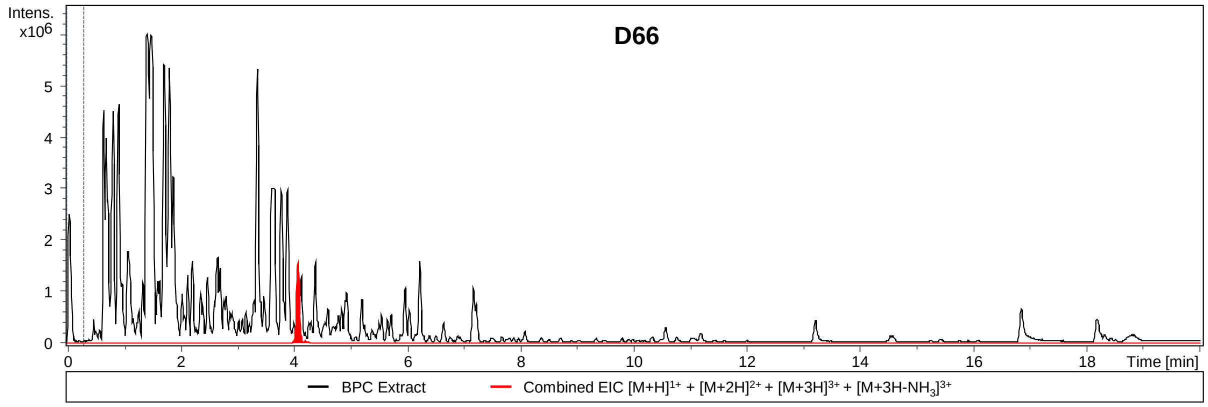


**Figure S 11: Chromatogram of the *E. coli* BL21 (DE3) pNOSO-darABCDE-66 extract**. The red trace shows the combined EIC for the $[M+H]^{1+}$, $[M+2H]^{2+}$, $[M+3H]^{3+}$ and $[M+3H\text{-}NH_3]^{3+}$ species of **D66** at their calculated masses, respectively (Table S 4) ±0.02 Da. The BPC of the whole extract from fermentation broth supernatant is presented in

black. , The L-cysteine of **D66** is linked to another L-cysteine and lactic acid via a disulfide bond (Table S 5, Figure S 11 and S 29), as was shown for **D6** and **D32** in previous studies.[5,6]

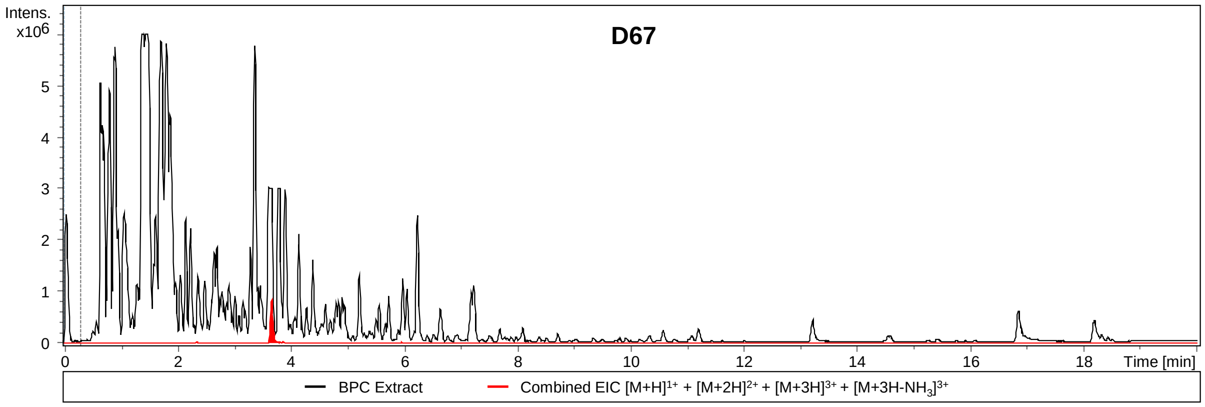


**Figure S 12: Chromatogram of the *E. coli* BL21 (DE3) pNOSO-darABCDE-67 extract**. The red trace shows the combined EIC for the $[M+H]^{1+}$, $[M+2H]^{2+}$, $[M+3H]^{3+}$ and $[M+3H-NH_3]^{3+}$ species of **D67** at their calculated masses, respectively (**Table S 4**) ±0.02 Da. The BPC of the whole extract from fermentation broth supernatant is presented in black.

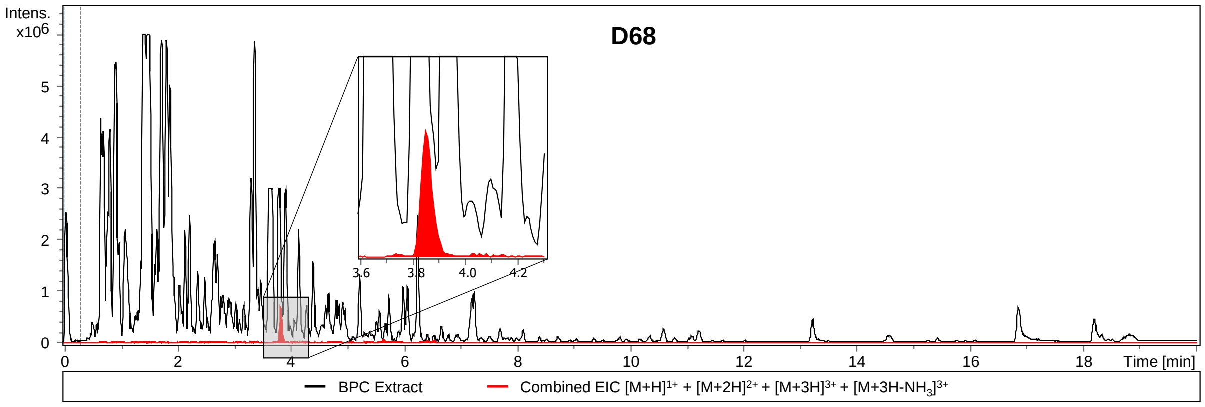


**Figure S 13: Chromatogram of the *E. coli* BL21 (DE3) pNOSO-darABCDE-68 extract**. The red trace shows the combined EIC for the $[M+H]^{1+}$, $[M+2H]^{2+}$, $[M+3H]^{3+}$ and $[M+3H-NH_3]^{3+}$ species of **D68** at their calculated masses, respectively (**Table S 4**) ±0.02 Da. The BPC of the whole extract from fermentation broth supernatant is presented in black.

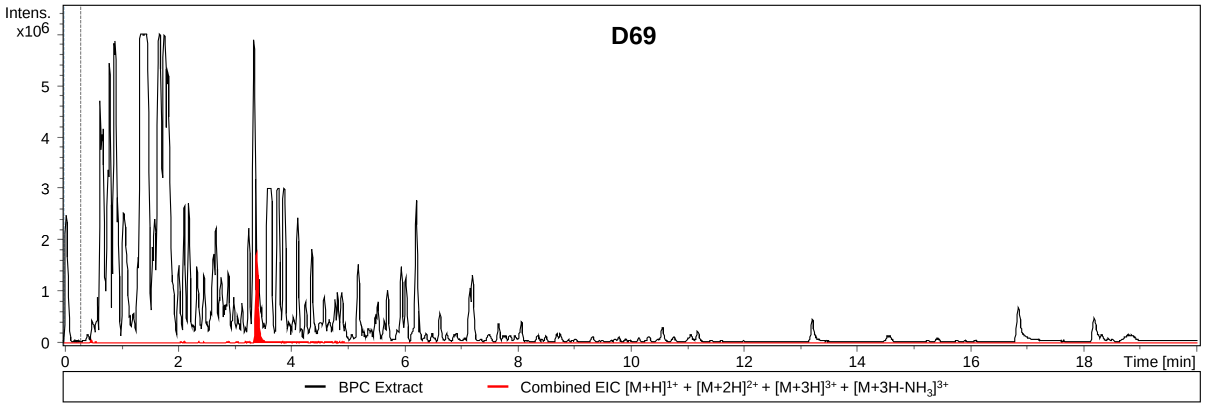


**Figure S 14: Chromatogram of the *E. coli* BL21 (DE3) pNOSO-darABCDE-69 extract**. The red trace shows the combined EIC for the $[M+H]^{1+}$, $[M+2H]^{2+}$, $[M+3H]^{3+}$ and $[M+3H-NH_3]^{3+}$ species of **D69** at their calculated masses, respectively (**Table S 4**) ±0.02 Da. The BPC of the whole extract from fermentation broth supernatant is presented in black.

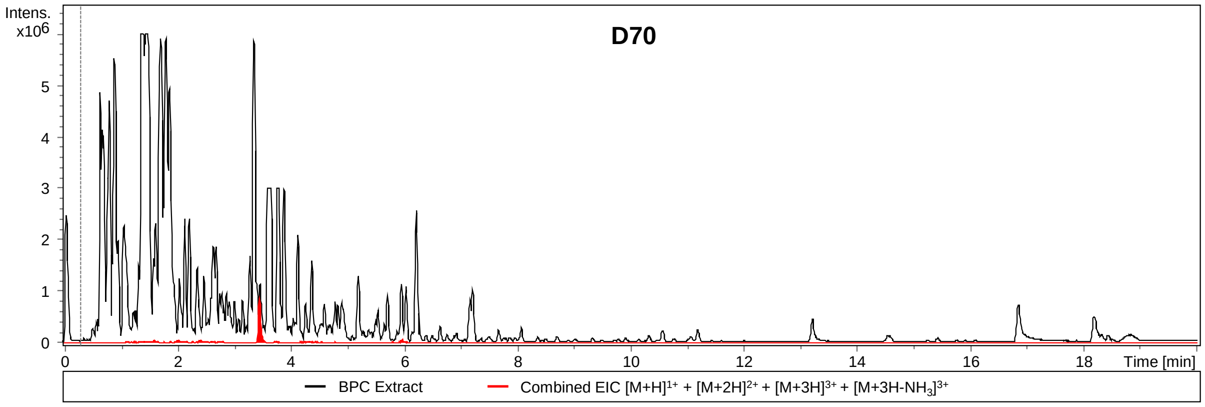


**Figure S 15: Chromatogram of the *E. coli* BL21 (DE3) pNOSO-darABCDE-70 extract**. The red trace shows the combined EIC for the $[M+H]^{1+}$, $[M+2H]^{2+}$, $[M+3H]^{3+}$ and $[M+3H-NH_3]^{3+}$ species of **D70** at their calculated masses, respectively (**Table S 4**) ±0.02 Da. The BPC of the whole extract from fermentation broth supernatant is presented in black.

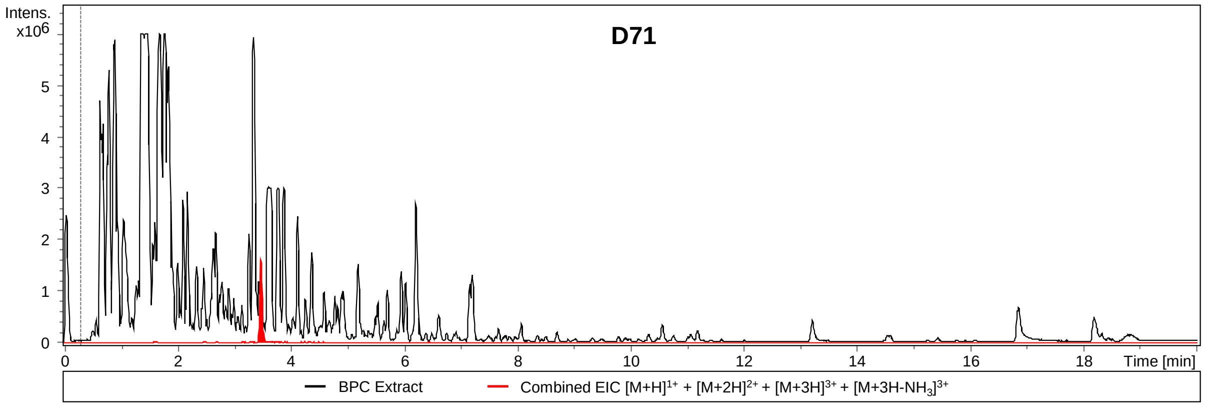


**Figure S 16: Chromatogram of the *E. coli* BL21 (DE3) pNOSO-darABCDE-71 extract**. The red trace shows the combined EIC for the $[M+H]^{1+}$, $[M+2H]^{2+}$, $[M+3H]^{3+}$ and $[M+3H-NH_3]^{3+}$ species of **D71** at their calculated masses, respectively (**Table S 4**) ±0.02 Da. The BPC of the whole extract from fermentation broth supernatant is presented in black.

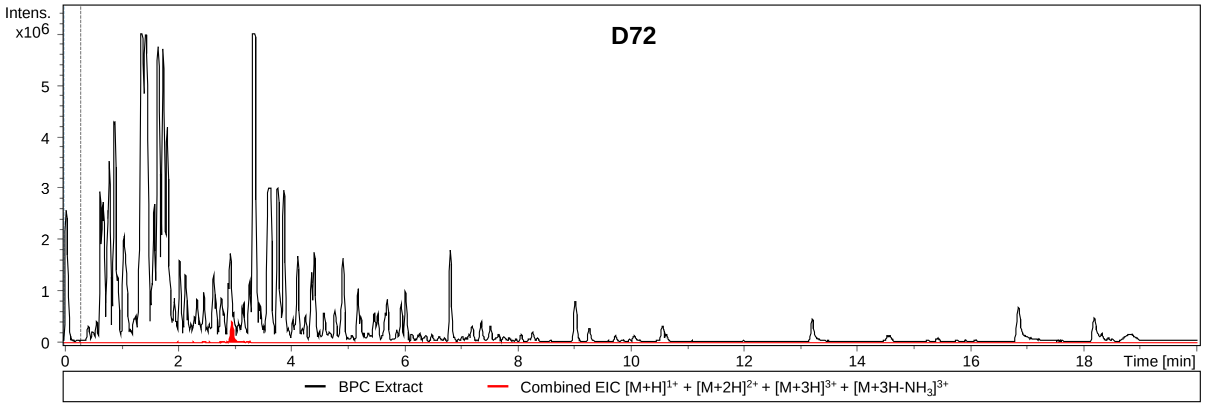


**Figure S 17: Chromatogram of the *E. coli* BL21 (DE3) pNOSO-darABCDE-72 extract**. The red trace shows the combined EIC for the $[M+H]^{1+}$, $[M+2H]^{2+}$, $[M+3H]^{3+}$ and $[M+3H-NH_3]^{3+}$ species of **D72** at their calculated masses, respectively (**Table S 4**) ±0.02 Da. The BPC of the whole extract from fermentation broth supernatant is presented in black.

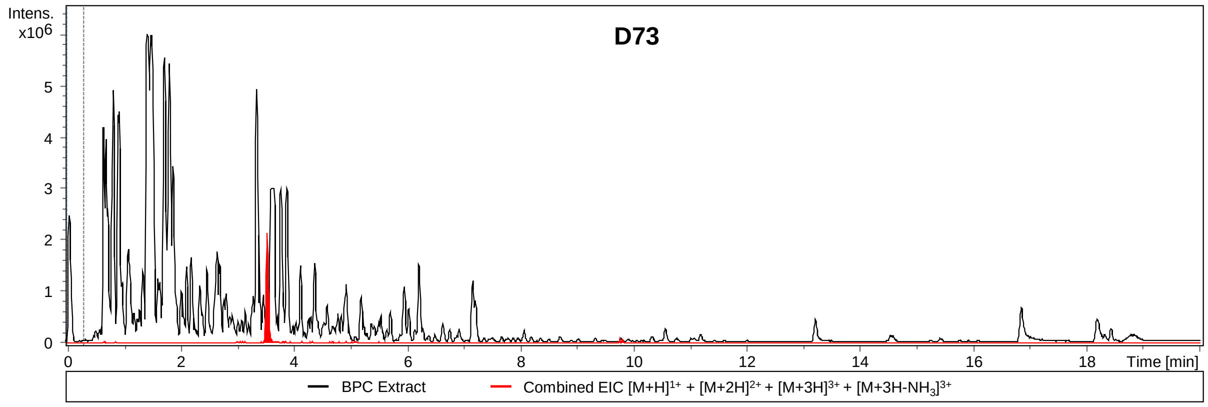


**Figure S 18: Chromatogram of the *E. coli* BL21 (DE3) pNOSO-darABCDE-73 extract**. The red trace shows the combined EIC for the $[M+H]^{1+}$, $[M+2H]^{2+}$, $[M+3H]^{3+}$ and $[M+3H-NH_3]^{3+}$ species of **D73** at their calculated masses, respectively (**Table S 4**) ±0.02 Da. The BPC of the whole extract from fermentation broth supernatant is presented in black.

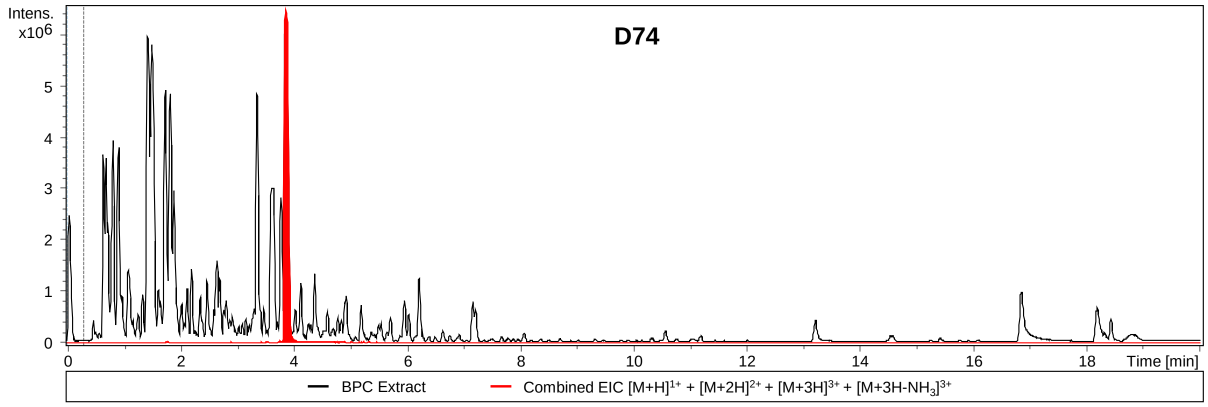


**Figure S 19: Chromatogram of the *E. coli* BL21 (DE3) pNOSO-darABCDE-74 extract**. The red trace shows the combined EIC for the $[M+H]^{1+}$, $[M+2H]^{2+}$, $[M+3H]^{3+}$ and $[M+3H-NH_3]^{3+}$ species of **D74** at their calculated masses, respectively (**Table S 4**) ±0.02 Da. The BPC of the whole extract from fermentation broth supernatant is presented in black.

3. $MS^2$ spectra analysis of darobactin analogues

The $MS^2$ spectra of all novel darobactin analogues are shown in Figure S 21-37. The blue quadratic symbols display the precursor ion, which was picked for fragmentation. The $[M-NH_3+2H]^{2+}$ fragment and the derivative related typical b2-ion with ammonia loss can be observed and allow the further verification of modifications (Figure S 20). The theoretical mass of all derivatives and the ions for the most characteristic fragmentation pattern are displayed in table S 5.

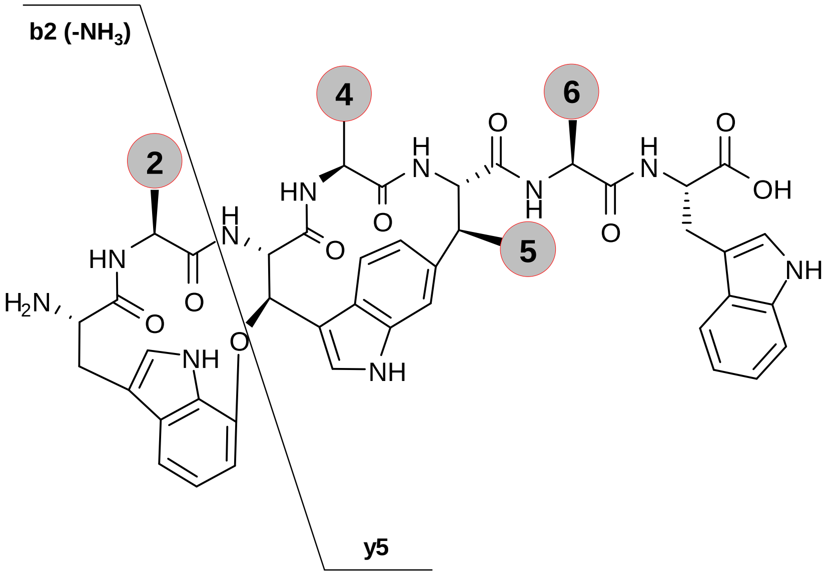


**Figure S 20: $MS^2$ fragmentation pattern of darobactin analogues**. The most characteristic ions in the $MS^2$ fragmentation pattern (b2 with or without ammonia loss and y5) are calculated and observed as described in the methods and displayed in Table S 5.

**Table S 5: Calculated (calc.) and observed (obs.) masses of the typical b2 and y5 fragment ions**. The novel darobactins with highlighted modifications in the core peptide in comparison to **D22** are summarized (differences in red). The calc. and obs. masses in the extracts of the $[M-NH_3+2H]^{2+}$ and the calc. and obs. typical b2 and y5 ion masses with ammonia loss are displayed.

| Darobactin | Core peptide | calc. $[M-NH_3+2H]^{2+}$ | obs. $[M-NH_3+2H]^{2+}$ | calc. b2 – $NH_3$ | obs. b2 – $NH_3$ | calc. y5 | obs. y5 |
|---|---|---|---|---|---|---|---|
| 58 | W E W T K R W | 543.7432 | 543.7384 | 315.0975 | 315.0959 | 772.3889 | 772.3822 |
| 59 | W K W T K R W | 543.2694 | 543.2674 | 314.1499 | 314.1434 | 772.3889 | 772.3831 |
| 60 | W D W T K R W | 536.7354 | 536.7287 | 301.0819 | 301.0796 | 772.3889 | 772.3799 |
| 61 | W Q W T K R W | 543.2512 | 543.2455 | 314.1135 | 314.1119 | 772.3889 | 772.3828 |
| 62 | W R W T K R W | 557.2673 | 557.2682 | 342.1561 | 342.1489 | 772.3889 | 772.3765 |
| 63 | W V W T K R W | 528.7561 | 528.7522 | 285.1234 | 285.1204 | 772.3889 | 772.3851 |
| 64 | W S W T K R W | 522.7380 | 522.7348 | 273.0870 | 273.0871 | 772.3889 | 772.3789 |
| 65 | W S W T K K W | 508.7349 | 508.7324 | 273.0870 | 273.1228 | 744.3828 | 744.3780 |
| 66 | W N W T K C W | 605.2012 | 605.2035 | 300.0979 | 300.0957 | 910.3222 | 910.3113 |
| 67 | W Y W T K R W | 560.7536 | 560.7478 | 349.1183 | 349.1139 | 772.3889 | 772.3809 |
| 68 | W L W T K R W | 535.7640 | 535.7581 | 299.1390 | 299.1383 | 772.3889 | 772.3808 |
| 69 | W Q W S K R W | 536.2434 | 536.2369 | 314.1135 | 314.1114 | 758.3733 | 758.3649 |
| 70 | W S W S K R W | 515.7301 | 515.7247 | 273.0870 | 273.0859 | 758.3733 | 758.3635 |
| 71 | W E W S K R W | 536.7354 | 536.7316 | 315.0975 | 315.0965 | 758.3733 | 758.3684 |
| 72 | W K W S K R W | 536.2616 | 536.2589 | 314.1499 | 314.1817 | 758.3733 | 758.3697 |
| 73 | W D W S K R W | 529.7276 | 529.7237 | 301.0819 | 301.0807 | 758.3733 | 758.3672 |
| 74 | W N W T K Q W | 522.2221 | 522.2198 | 300.0979 | 300.0967 | 744.3464 | 744.3422 |

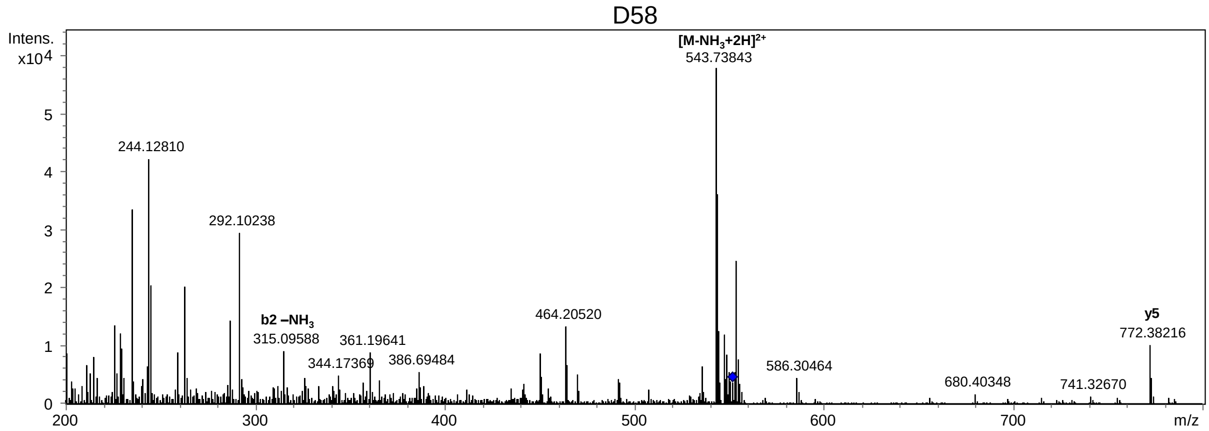


**Figure S 21: MS$^2$ spectrum for D58.**

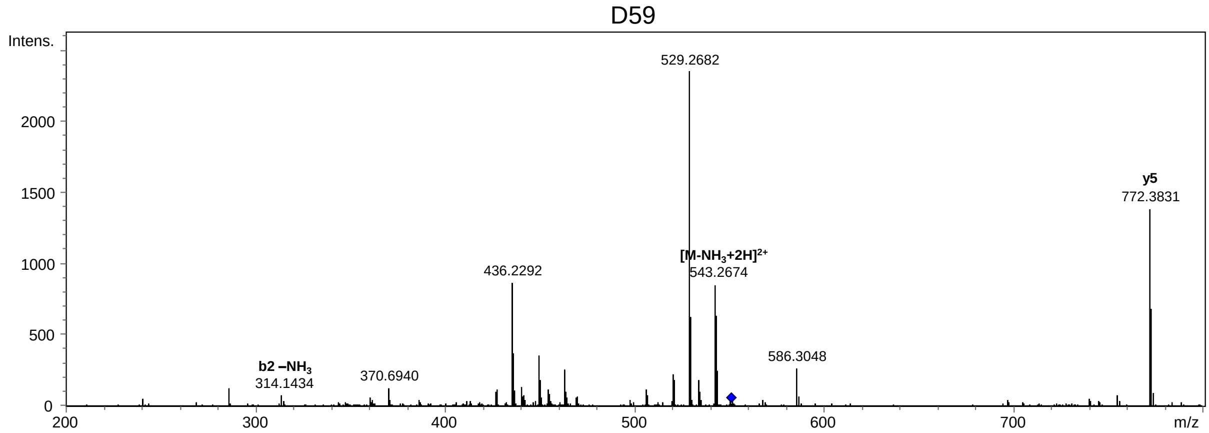


**Figure S 22: MS$^2$ spectrum for D59.**

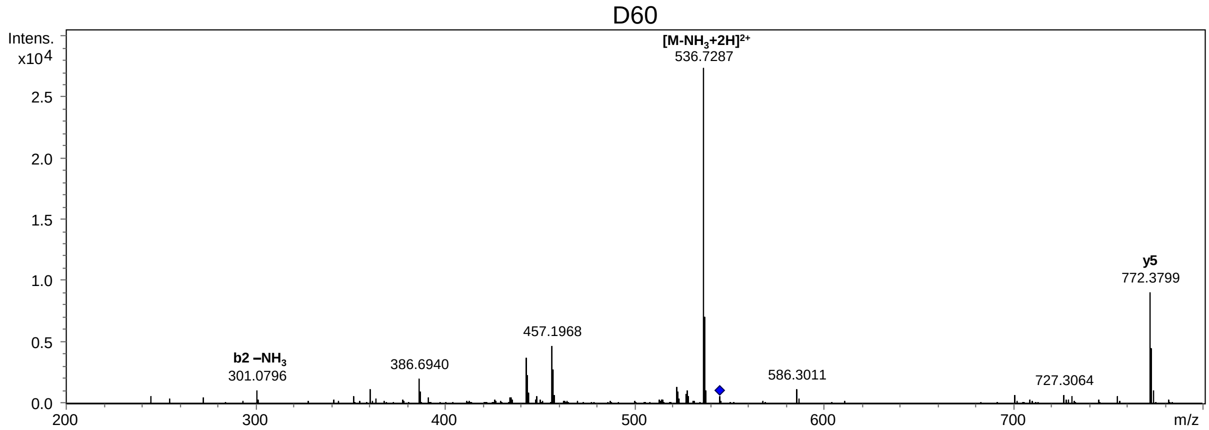


**Figure S 23: MS$^2$ spectrum for D60.**

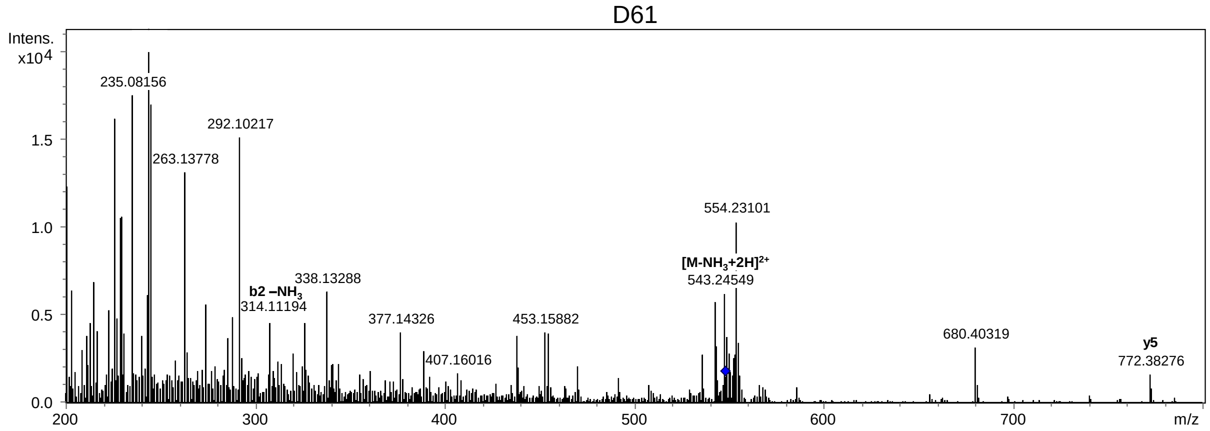


**Figure S 24: MS$^2$ spectrum for D61.**

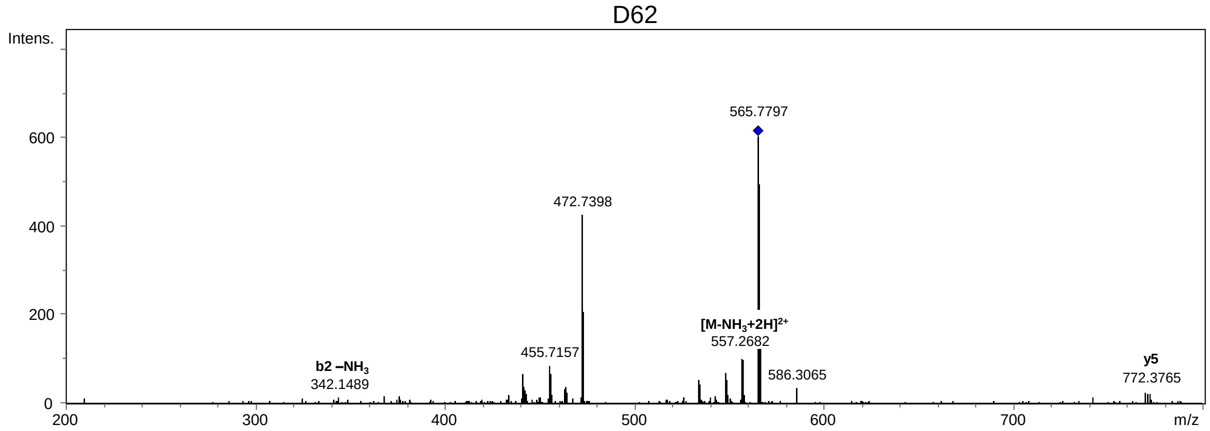


**Figure S 25: MS$^2$ spectrum for D62.**

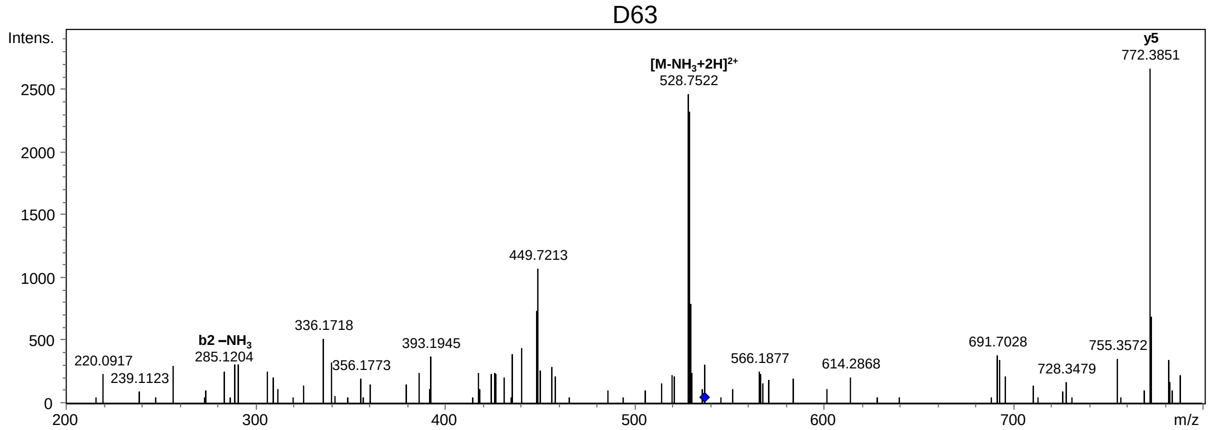


**Figure S 26: MS$^2$ spectrum for D63.**

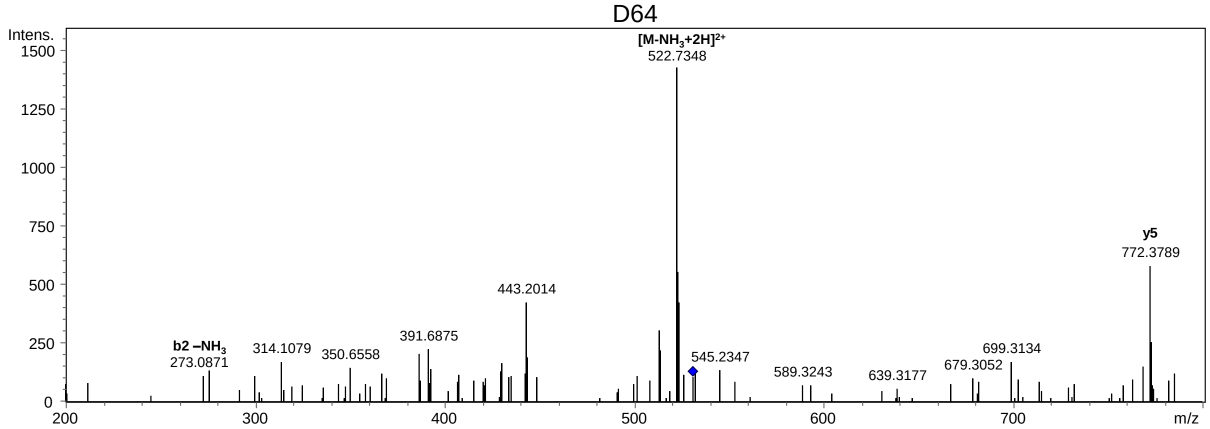


**Figure S 27: MS$^2$ spectrum for D64.**

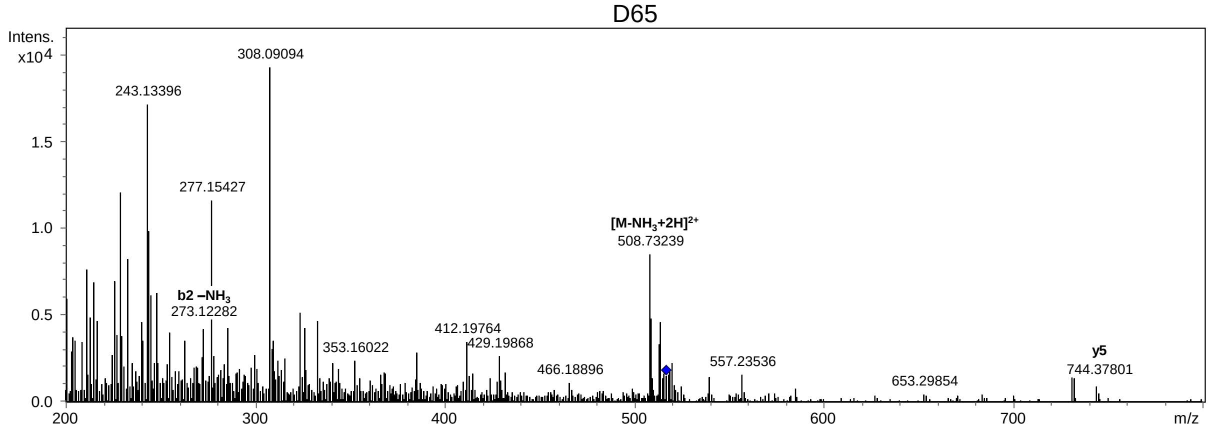


**Figure S 28: MS$^2$ spectrum for D65.**

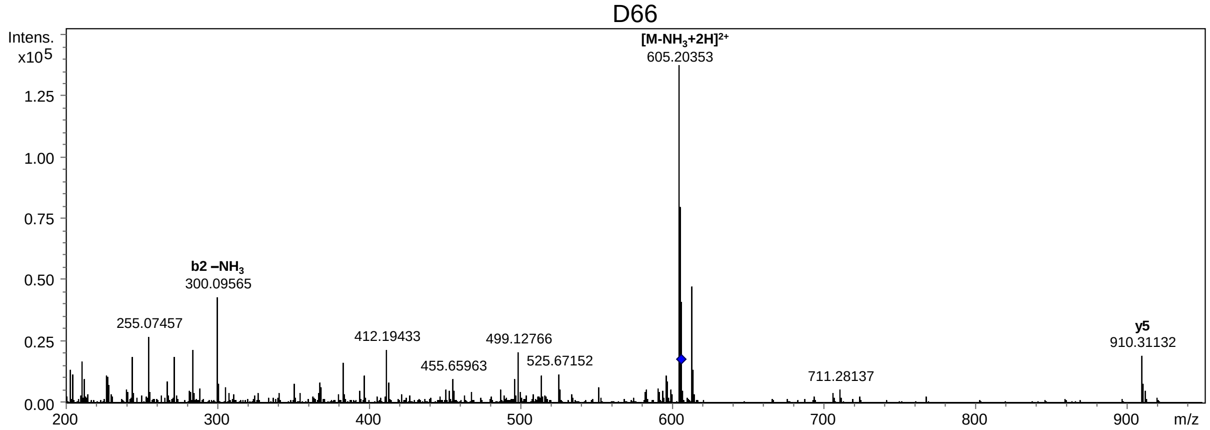


**Figure S 29: MS$^2$ spectrum for D66.**

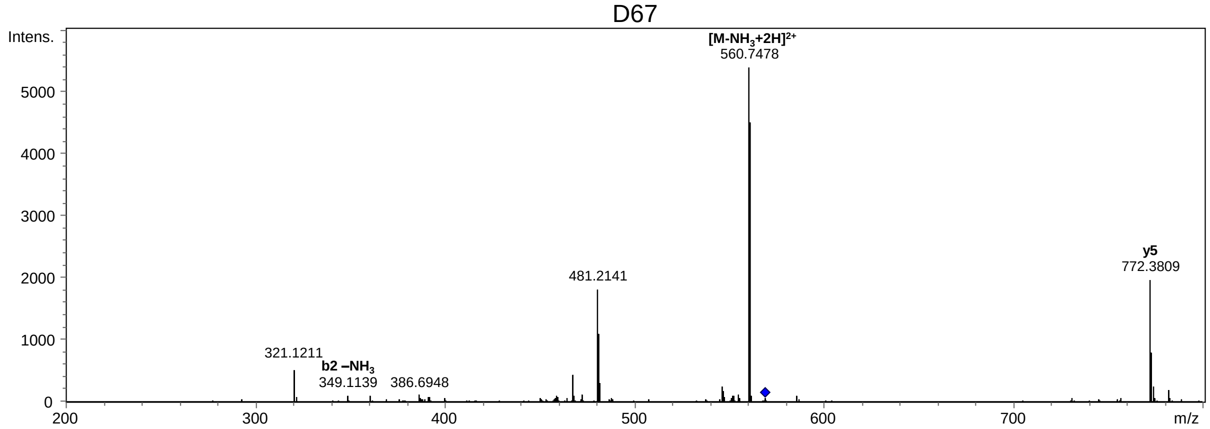


**Figure S 30: MS$^2$ spectrum for D67.**

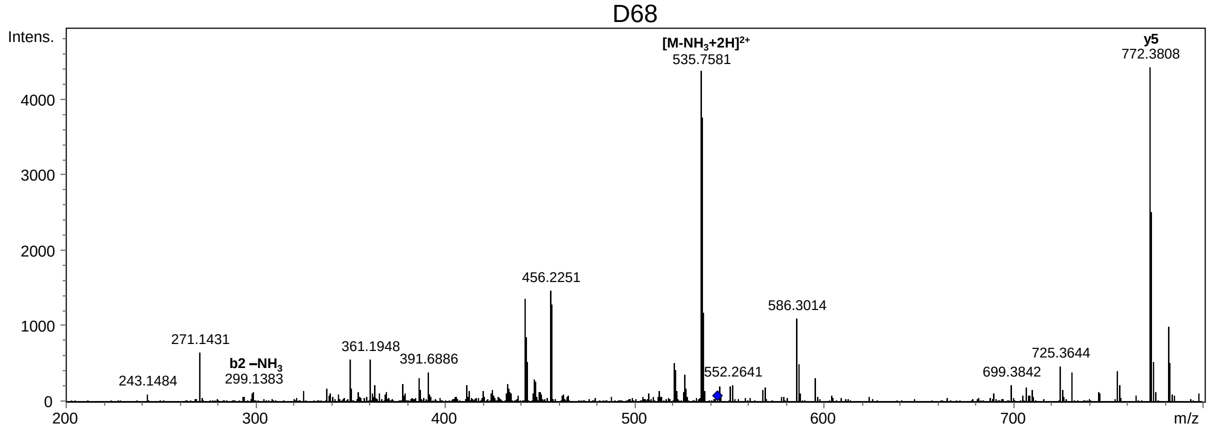


**Figure S 31: MS$^2$ spectrum for D68.**

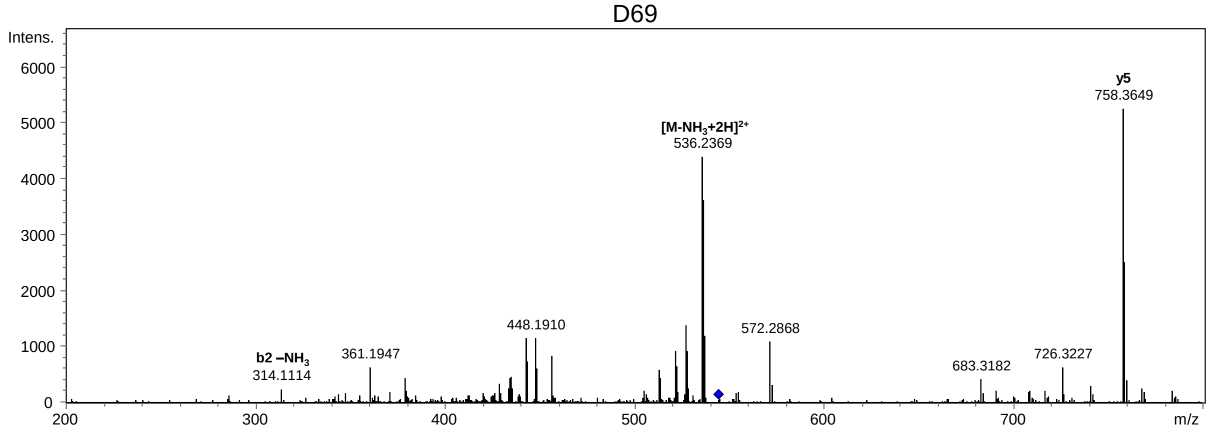


**Figure S 32: MS$^2$ spectrum for D69.**

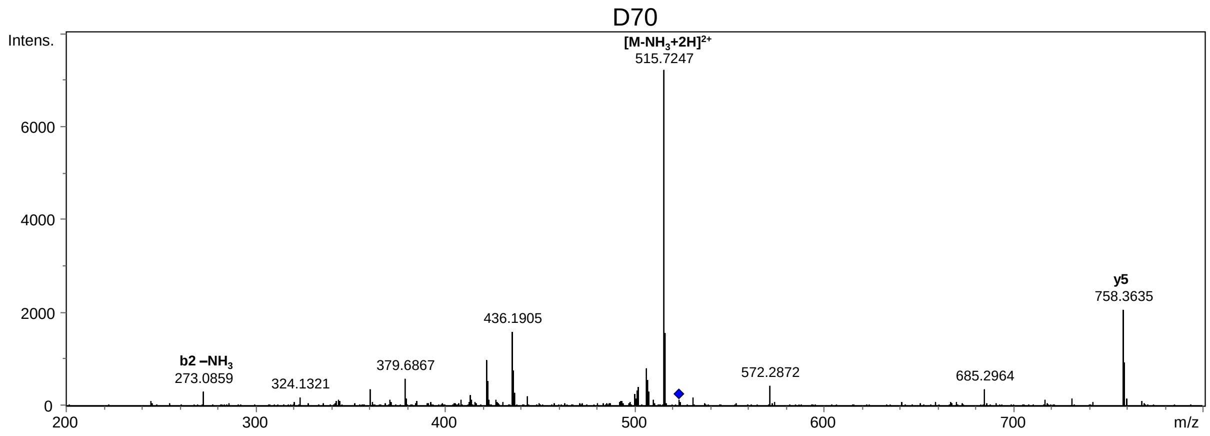


**Figure S 33: MS$^2$ spectrum for D70.**

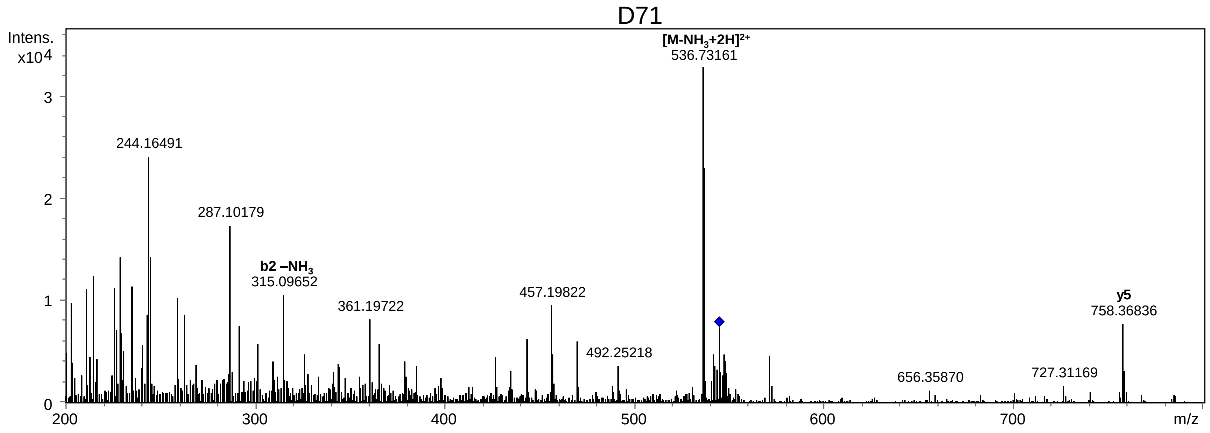


**Figure S 34: MS$^2$ spectrum for D71.**

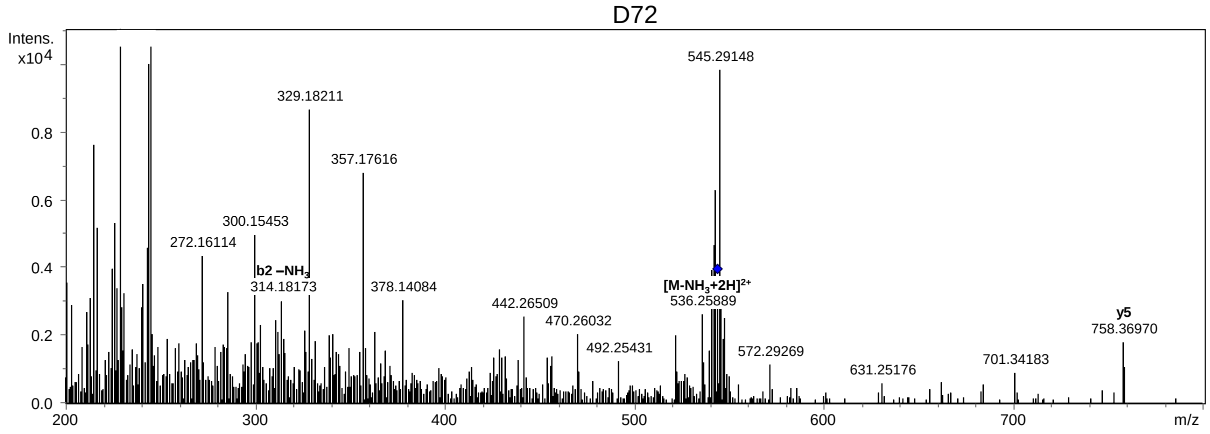


**Figure S 35: MS$^2$ spectrum for D72.**

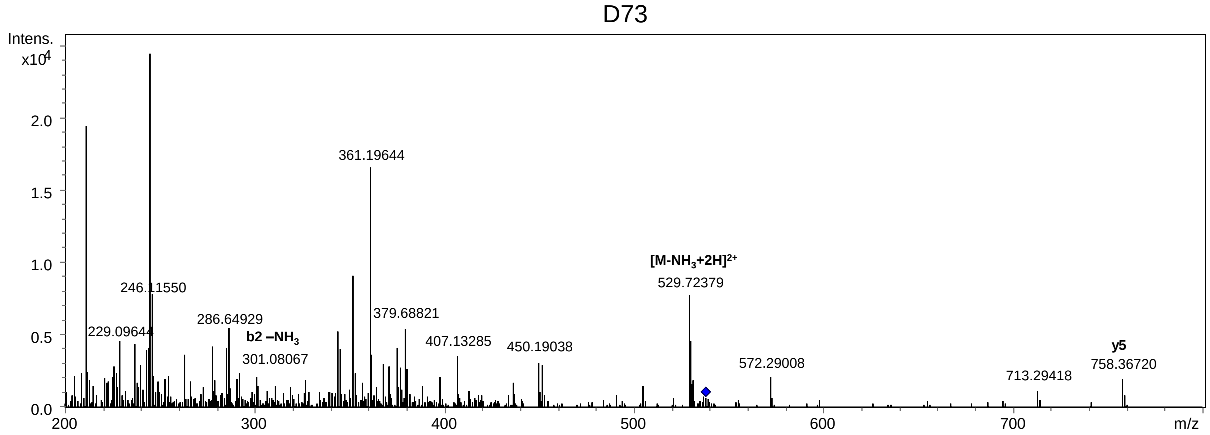


**Figure S 36: MS$^2$ spectrum for D73.**

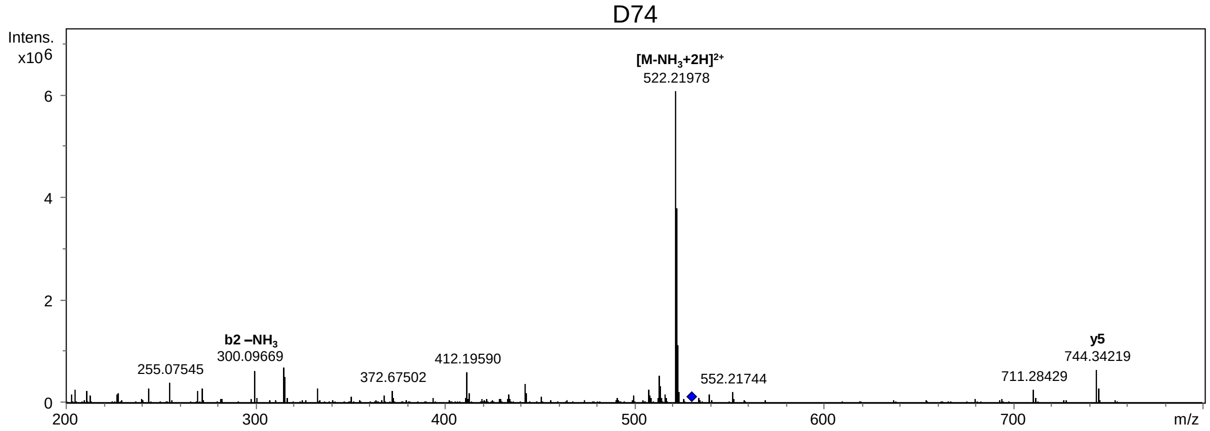


**Figure S 37: MS$^2$ spectrum for D74.**

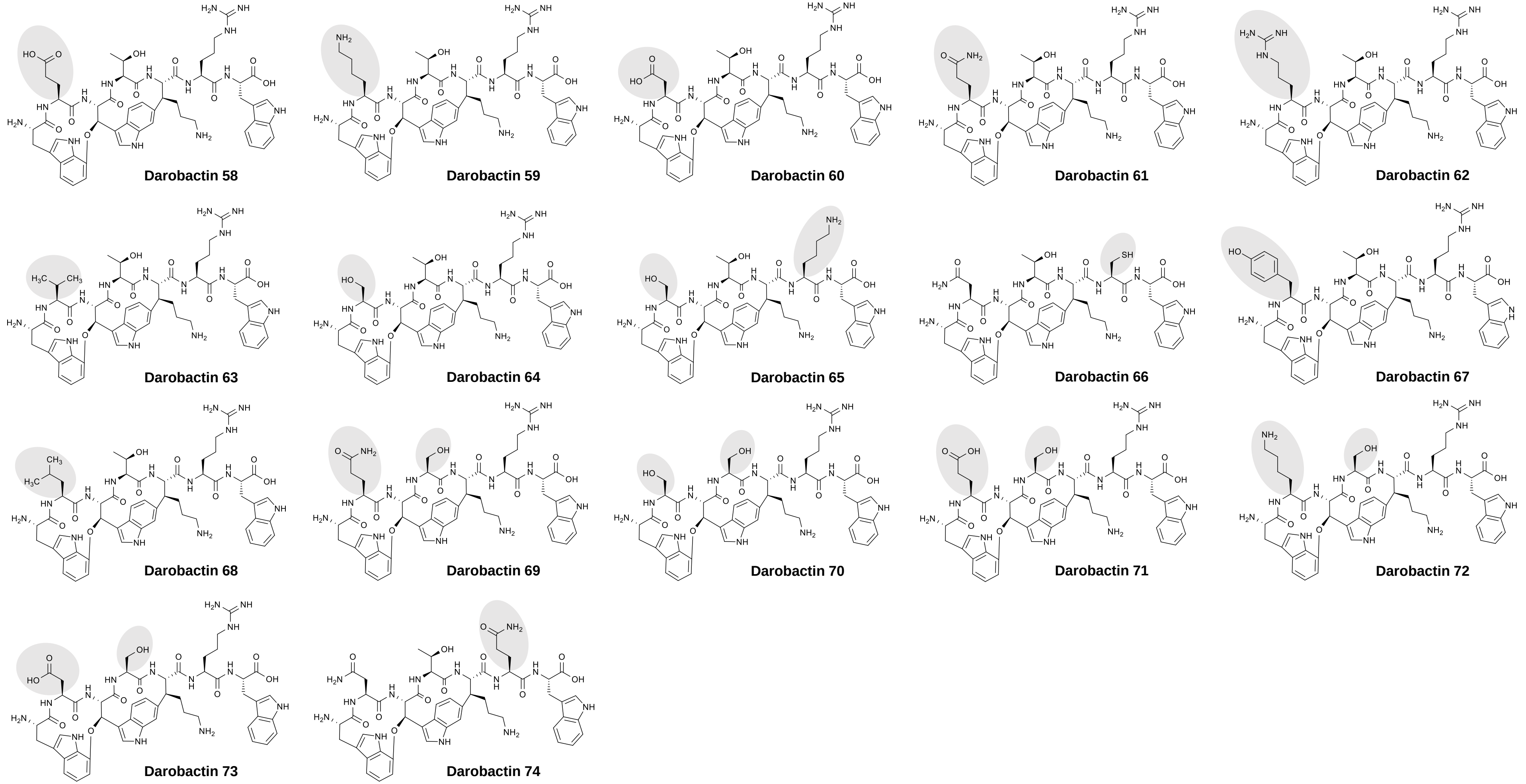


**Figure S 38: Overview of all generated novel darobactin analogues**. **D58** to **D74** are displayed and positions of heptapeptide with amino acid exchange compared to **D22** amino acid sequence were highlighted in grey. Structures were predicted using $MS^2$ analysis. **D69** was verified via NMR spectrometry (Table S 7, Figure S 39 – S44).

4. Antibiogram of clinical *P. aeruginosa* isolates

**Table S 6: Antibiogram of clinical *P. aeruginosa* isolates**. Antibacterial activity of **D22** and **D69** were evaluated in parallel with meropenem and ciprofloxacin (CIP) as controls (Table 4). The minimal inhibitory concentration (MIC) of **D22** and **D69** is comparable to the antibiotics Mereopenem and CIP. Broncheoalveolar lavage (BAL); permanent catheter urine (PK-urine); resistant (R); multi-resistant pathogen (MRP); multi-resistant Gram-negative pathogens with resistance against four of four groups of antibiotics (4MNGR).

| | | resistance profile, MIC [μg mL$^{-1}$] | | |
|---|---|---|---|---|
| | material | MRP | Meropenem | CIP |
| *P. aeroginosa* 83979 | BAL | 4MRGN | > 16 (R) | > 4 (R) |
| *P. aeroginosa* 84389 | PK-urine | 4MRGN | 16 (R) | 4 (R) |

5. Quantification of production

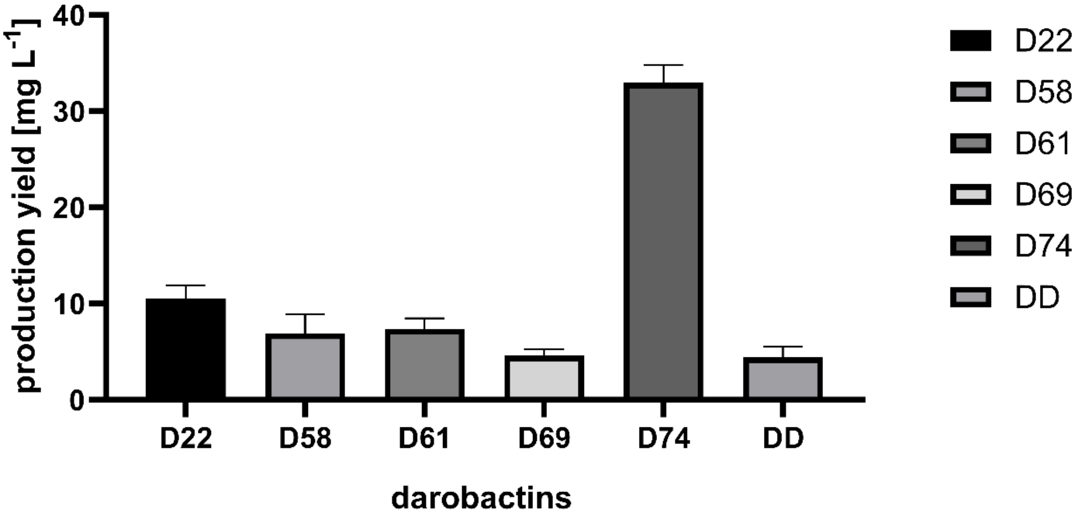


**Figure S 39: Production yield of darobactin D22, D69 and D74**. HPLC UV quantification of production yield was calculated by integration across the chromatographic UV peak area of each derivative. Eight (**D22**, **D69**), ten (**D74**) and twelve (**D58**, **D61**, **DD**) replicates were compared. The production yields of **D22** achieved 10.5 ± 0.9 mg per liter, of **D58** 6.9 ± 2.0 mg per liter, of **D61** 7.4 ± 1.1 mg per liter, of **D69** 4.6 ± 0.6 mg per liter**,** of **D74** 33 ± 1.7 mg per liter and of **DD** 4.4 ± 1.1 mg per liter, respectively.

6. NMR spectroscopic data

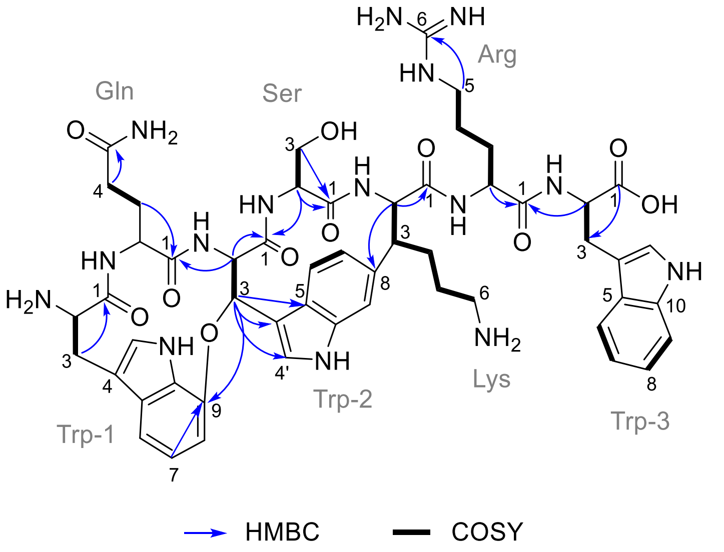


**Table S 7: NMR Spectroscopic Data for D69[a].**

| | Pos. | $\delta_C$[b], type | $\delta_H$[c], (*J* in Hz) | COSY[d] | HMBC[e] |
|---|---|---|---|---|---|
| Trp-3 | 1 | 174.9, C | - | - | - |
| | 2 | 53.7, CH | 4.99, t (5.5) | 3 | 1, 3, 4, Arg-1 |
| | 3 | 26.6, $CH_2$ | 3.63, m | 2 | 1, 2, 4, 4’, 5 |
| | 4’ | 124.0, CH | 7.56, s | - | 3, 4, 5, 9, 10 |
| | 4 | 109.0, C | - | - | - |
| | 5 | 127.0, C | - | - | - |
| | 6 | 118.1, CH | 7.95, br d (7.5) | 7 | 4, 5, 8, 10 |
| | 7 | 118.9, CH | 7.45, m | 6, 8 | 5, 6, 8, 9 |
| | 8 | 121.4, CH | 7.53, m | 7, 9 | 6, 10 |
| | 9 | 111.4, CH | 7.81, br d (7.9) | 8 | 5, 7 |
| | 10 | 135.9, C | - | - | - |
| Arg | 1 | 171.9, C | - | - | - |
| | 2 | 52.9, CH | 4.60, br t (6.3) | 3ab | 1, 3 |
| | 3a | 27.8, $CH_2$ | 1.88, m | 2, 3b, 4 | 1, 2, 4, 5 |
| | 3b | | 2.02, m | 2, 3a, 4 | 1, 2, 4, 5 |
| | 4 | 23.9, $CH_2$ | 1.74, m | 3, 5 | 2, 5 |
| | 5 | 40.0, $CH_2$ | 3.34, m | 4 | 3, 4, 6 |
| | 6 | 156.3, C | - | - | - |
| Lys | 1 | 170.8, C | - | - | - |
| | 2 | 59.7, CH | 4.39, br d (10.3) | 3 | 1, 3, 4, Ser-1, Trp2-8 |
| | 3 | 47.7, CH | 3.21, m | 2, 4b | 2, 4 |
| | 4a | 25.2, $CH_2$ | 1.82, m | 4b, 6 | 6 |
| | 4b | | 2.12, m | 4a, 3 | - |
| | 5 | 25.1, $CH_2$ | 2.04, m | 4ab, 6 | - |
| | 6 | 38.9, $CH_2$ | 3.08, m | 4a, 5 | 4 |
| Ser | 1 | 167.5, C | - | - | - |
| | 2 | 53.7, CH | 4.24, m | 3ab | 1, Trp2-1 |
| | 3a | 61.6, $CH_2$ | 3.40, m | 2 | 1 |
| | 3b | | 3.46, m | 2 | 1 |
| Trp-2 | 1 | 167.6, C | - | - | - |
| | 2 | 62.9, CH | 4.92, d (8.9) | 3 | 1, 3, 4, Gln-1 |
| | 3 | 76.2, CH | 6.39, br d (8.9) | 2 | 2, 4, 4', 5, 9 |
| | 4’ | 123.8, CH | 8.09, s | - | 3, 4, 5, 10 |
| | 4 | 111.6, C | - | - | - |
| | 5 | 124.6, C | - | - | - |
| | 6 | 116.7, CH | 7.69, d (7.3) | 7 | 8, 10 |
| | 7 | 124.7, CH | 7.18, d (7.3) | 6 | 5, 9, Lys-3 |
| | 8 | 132.3, C | - | - | - |
| | 9 | 110.1, CH | 7.67, s | - | 5, 8, 10, Lys-3 |
| | 10 | 136.6, C | - | - | - |
| Gln | 1 | 169.0, C | - | - | - |
| | 2 | 52.3, CH | 3.33, m | 3 | 1 |

| | | | | | |
|---|---|---|---|---|---|
| | 3 | 28.7, $CH_2$ | 1.78, m | 4 | - |
| | 4 | 30.1, $CH_2$ | 2.20, m | 3 | 2, 3, 5 |
| | 5 | 177.0, C | - | - | - |
| Trp-1 | 1 | 167.7, C | - | - | - |
| | 2 | 54.3, CH | 4.27, m | 3ab | 1, 3 |
| | 3a | 25.9, $CH_2$ | 3.80, dd (13.3, 6.8) | 2, 3b | 1, 2, 4, 4’, 5 |
| | 3b | | 3.54, m | 2, 3a | 2, 4, 4’, 5 |
| | 4’ | 124.0, CH | 7.59, s | - | 4, 5, 9, 10 |
| | 4 | 107.8, C | - | - | - |
| | 5 | 128.7, C | - | - | - |
| | 6 | 119.6, CH | 7.40, m | 7 | 9, 10 |
| | 7 | 113.0, CH | 7.44, m | 6 | 5, 8, 9 |
| | 8 | 108.0, CH | 7.44, m | - | 7, 9, 10 |
| | 9 | 145.0, C | - | - | - |
| | 10 | 128.6, C | - | - | - |

[a]Recorded in $D_2O$/acetonitrile-$d_3$ (2:1) + 1% formic acid-$d_4$ at 318 K. [b]Acquired at 175 MHz, adjusted to the solvent signal of acetonitrile-$d_3$ ($\delta_C$ 118.69 ppm). [c]Acquired at 700 MHz, adjusted to the solvent signal of $D_2O$ ($\delta_H$ 4.75 ppm). [d]Proton showing COSY correlation to indicated protons. [e]Proton showing HMBC correlation to indicated carbon.

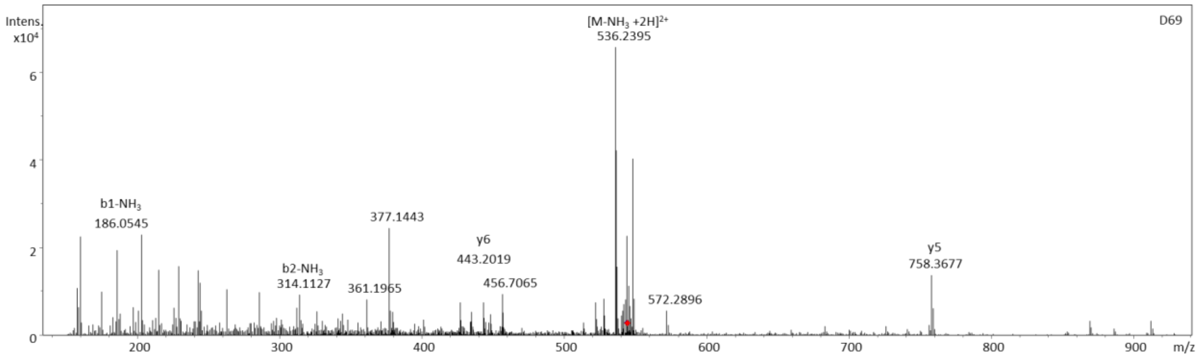


**Figure S 40: MS$^2$ spectrum of D69**. A red rhombus highlights the precursor ion with *m/z* 544.75490, which was picked for fragmentation. Furthermore, the $[M\text{-}NH_3\text{+}2H]^{2+}$ fragment and the characteristic b2-$NH_3$ and y5 fragment can be observed.

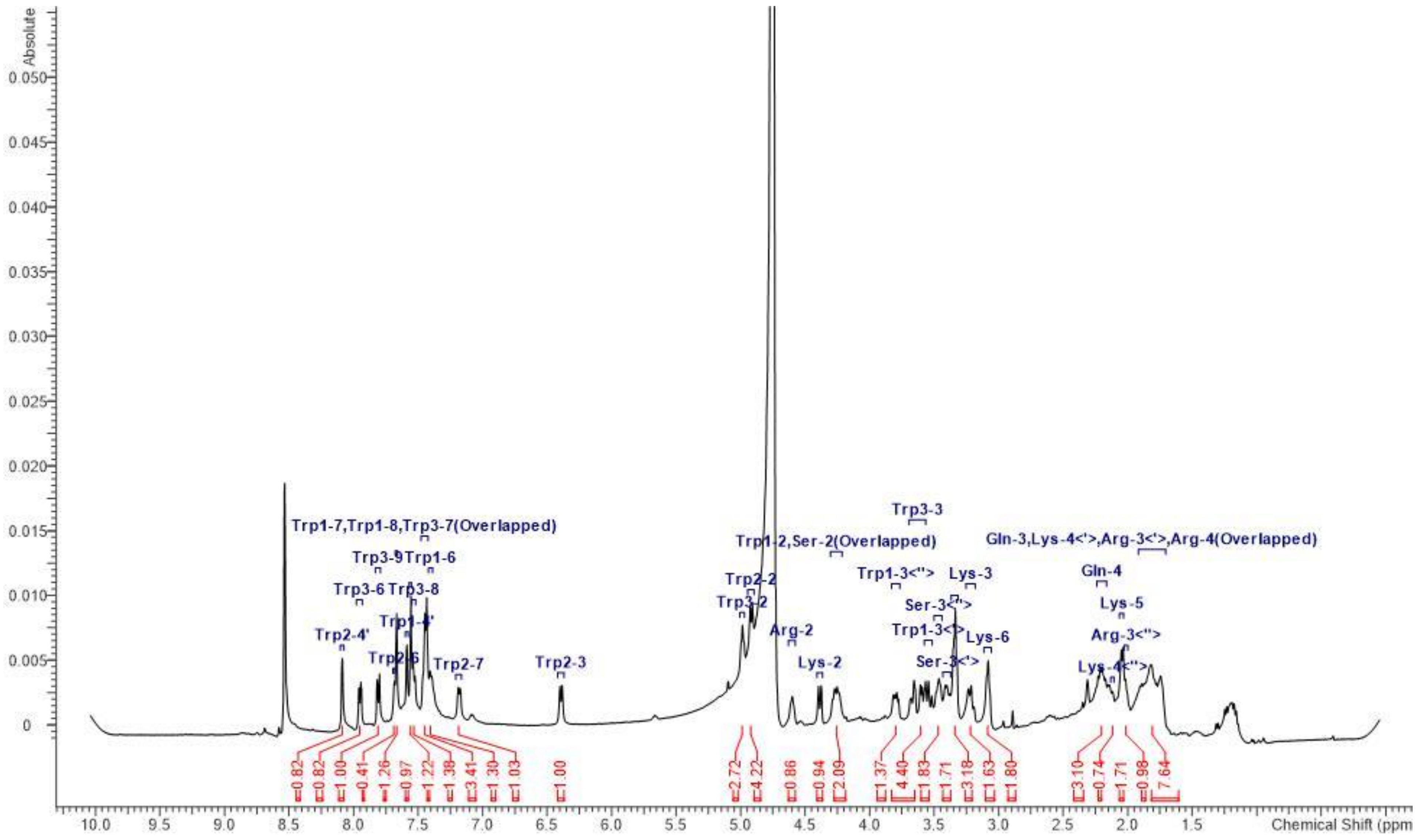


**Figure S 41: $^{1}$H NMR of D69 recorded in $D_2O$/acetonitrile-$d_3$ (2:1) + 1% formic acid-$d_4$ at 318 K.**

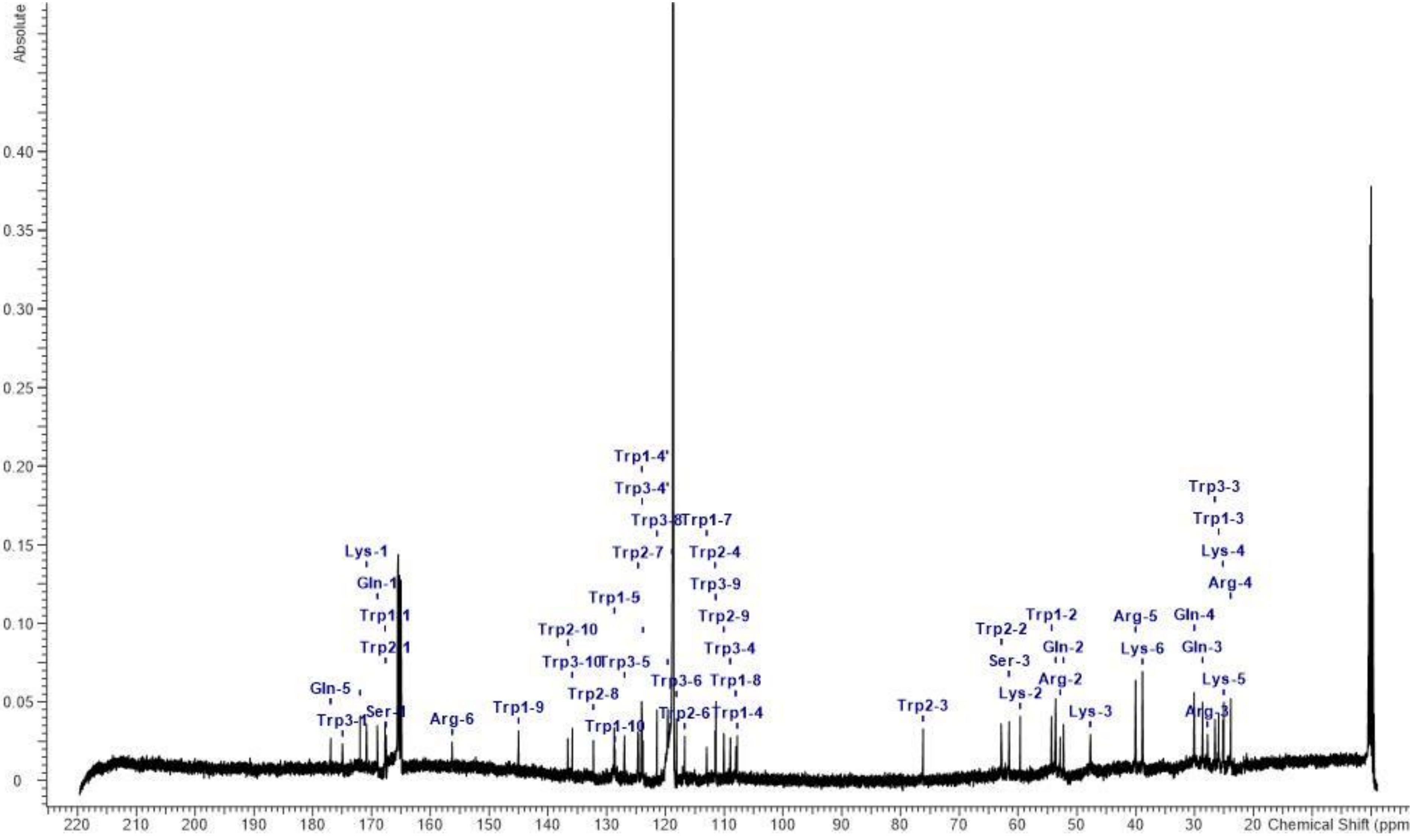


**Figure S 42: $^{13}$C NMR of D69 recorded in $D_2O$/acetonitrile-$d_3$ (2:1) + 1% formic acid-$d_4$ at 318 K.**

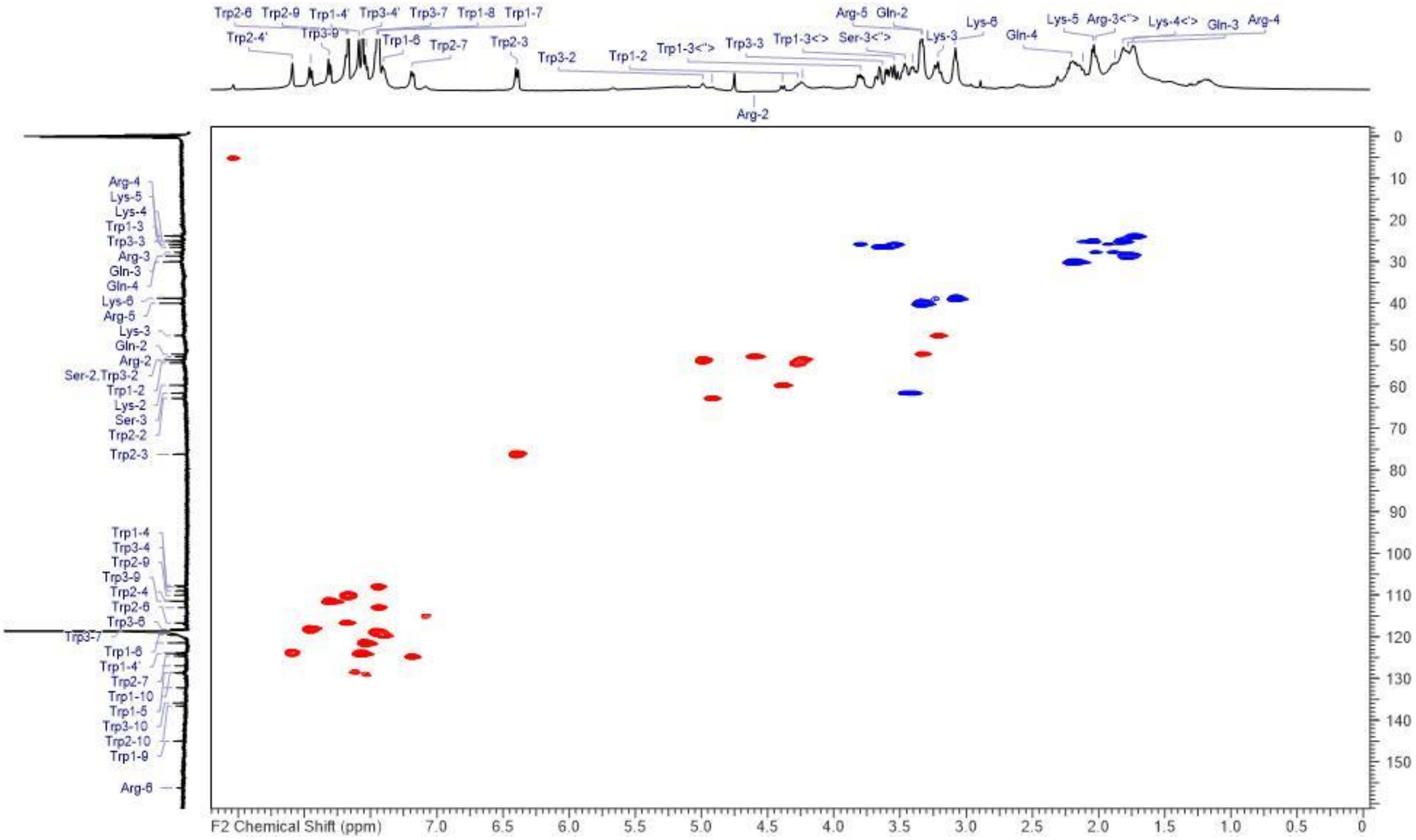


**Figure S 43: HSQC NMR of D69 recorded in $D_2O$/acetonitrile-$d_3$ (2:1) + 1% formic acid-$d_4$ at 318 K.**

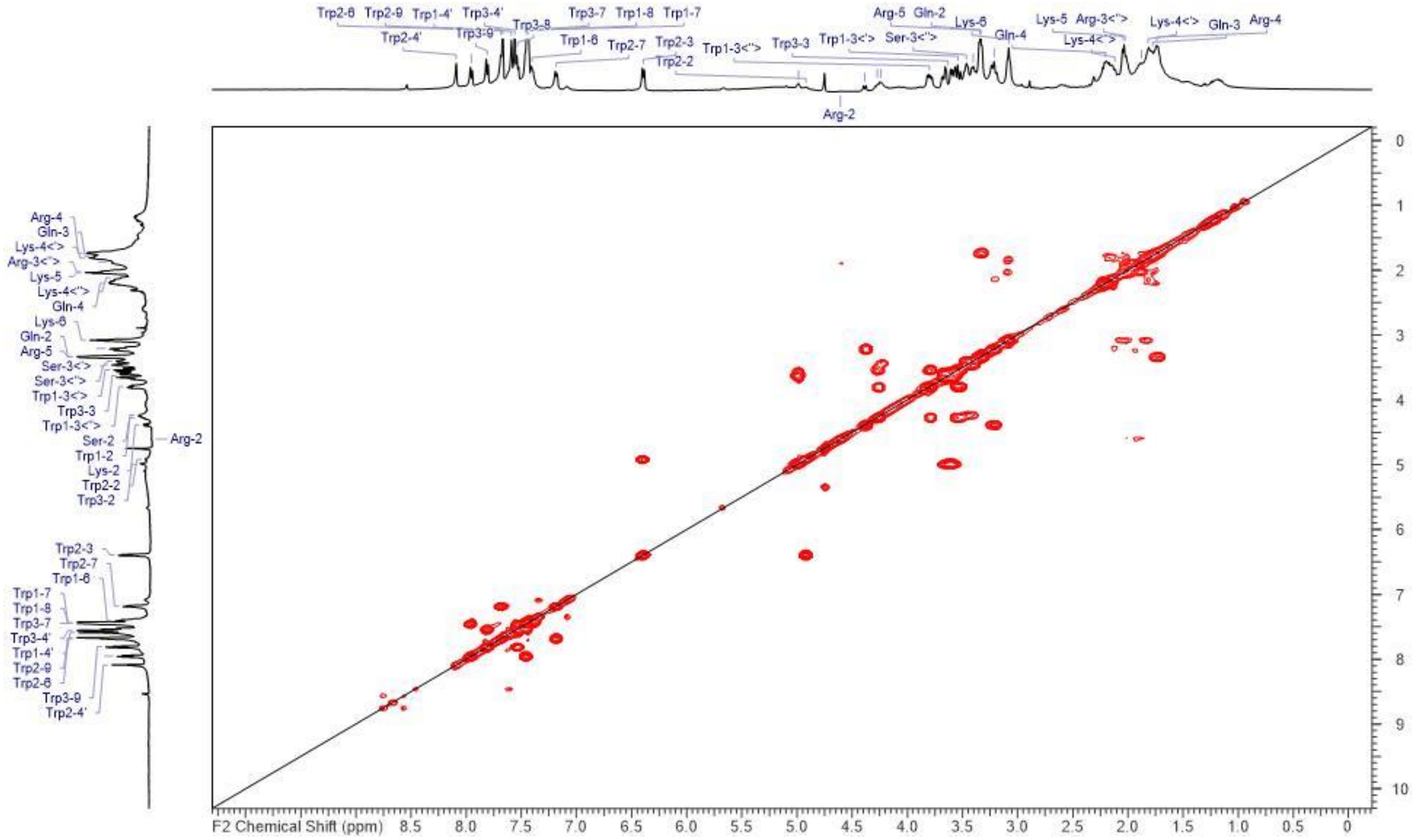


**Figure S 44: COSY NMR of D69 recorded in $D_2O$/acetonitrile-$d_3$ (2:1) + 1% formic acid-$d_4$ at 318 K.**

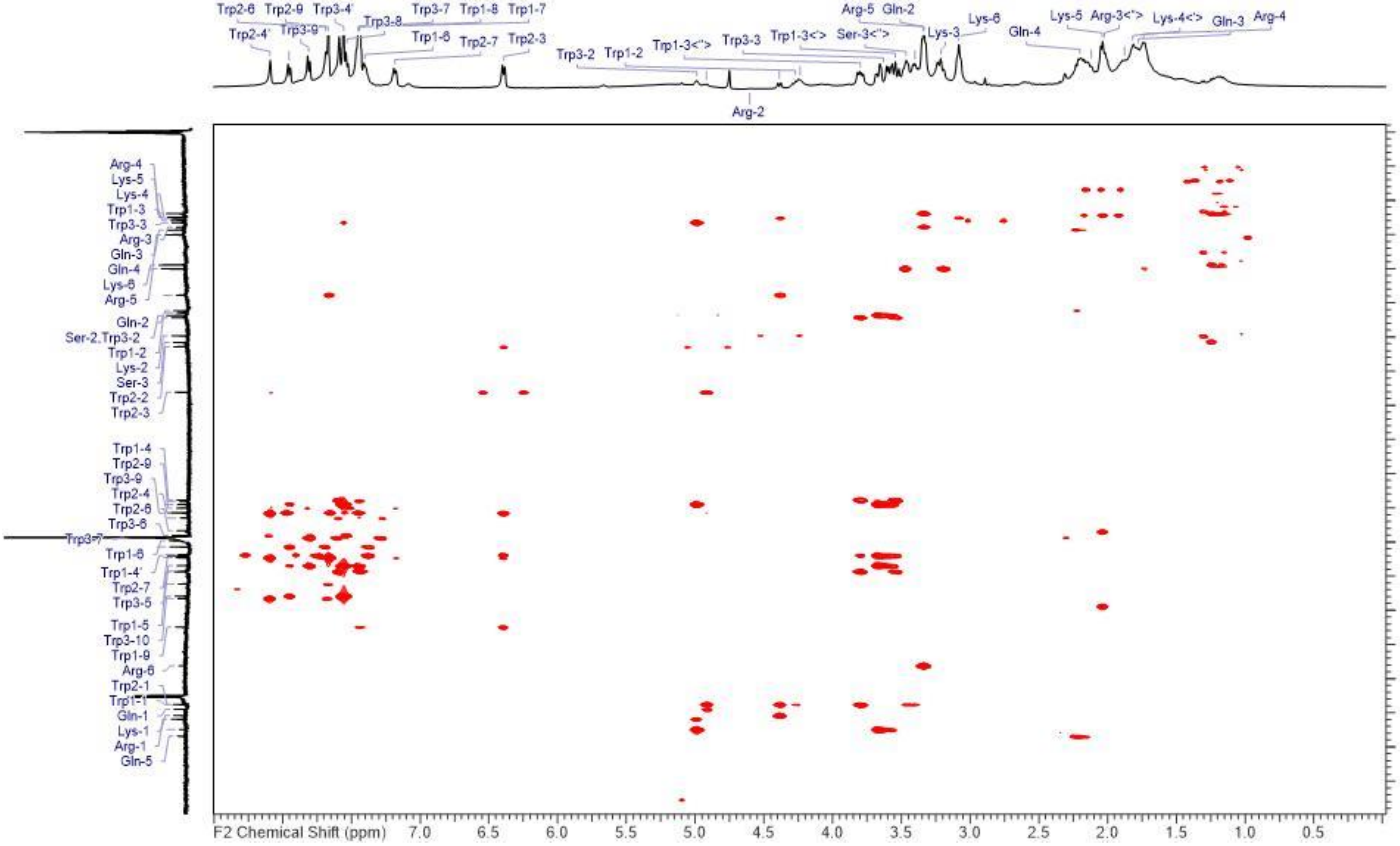


**Figure S 45: HMBC NMR of D69 recorded in $D_2O$/acetonitrile-$d_3$ (2:1) + 1% formic acid-$d_4$ at 318 K.**

7. HPLC traces of pure compounds

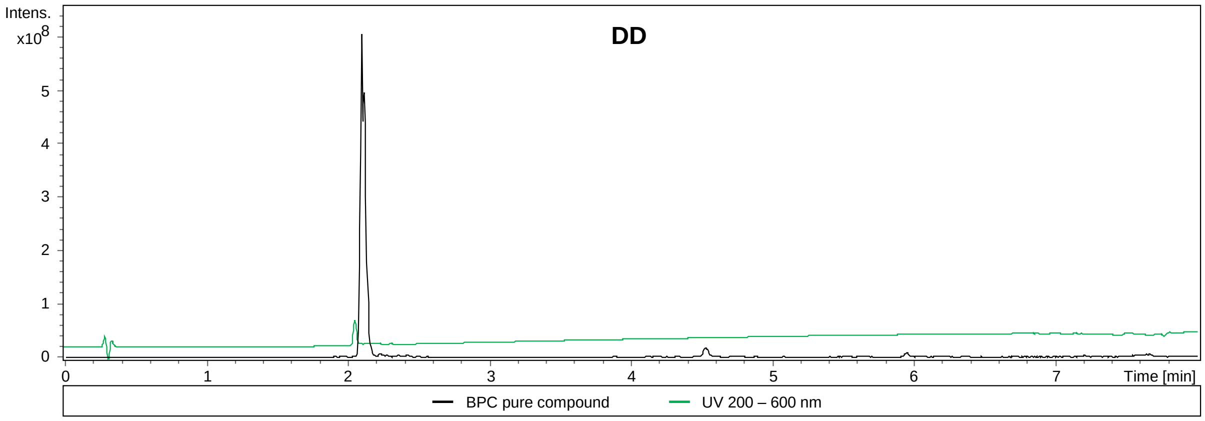


**Figure S 46: Chromatogram of purified DD.** The green trace shows the UV at 200–600 nm and the black trace shows the BPC. Acquired using amaZon speed.

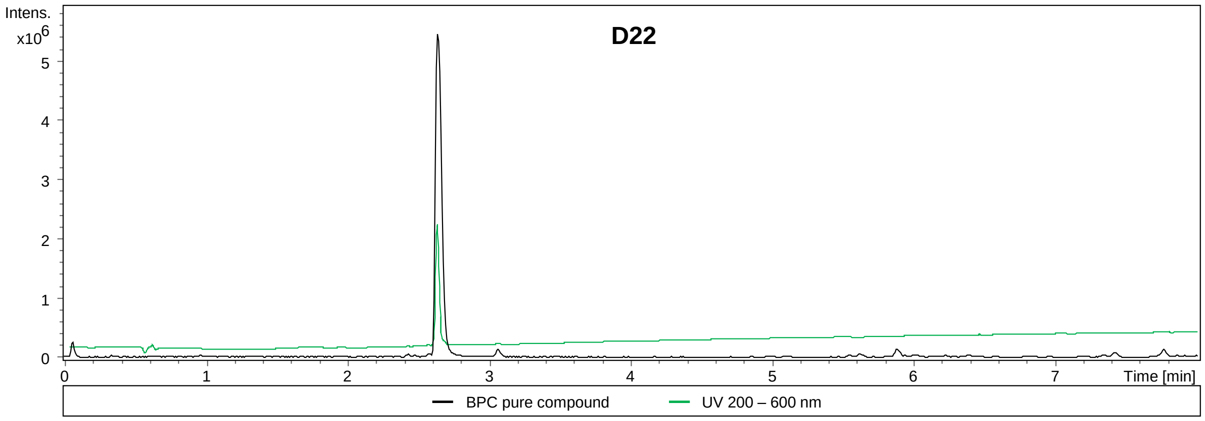


**Figure S 47: Chromatogram of purified D22.** The green trace shows the UV at 200–600 nm and the black trace shows the BPC. Acquired using maXis 4G.

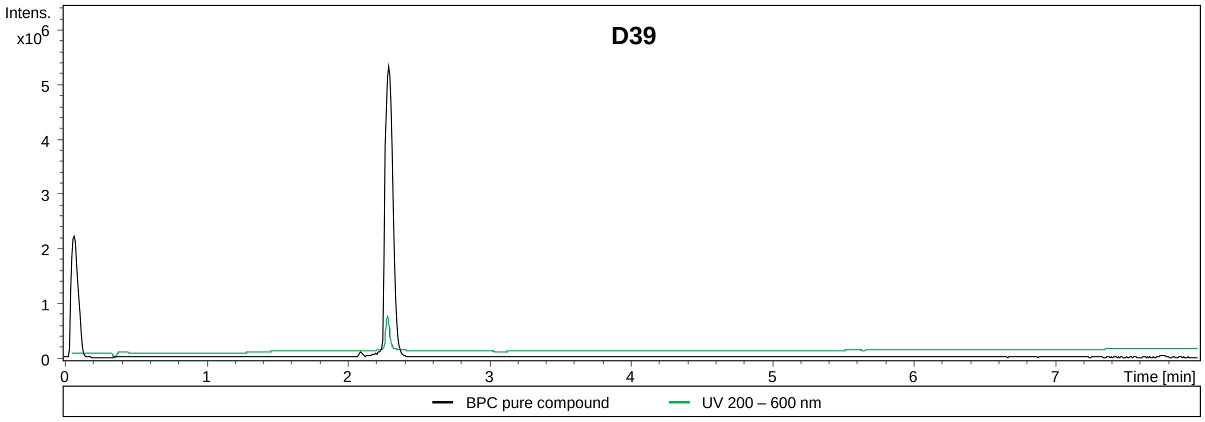


**Figure S 48: Chromatogram of purified D39.** The green trace shows the UV at 200–600 nm and the black trace shows the BPC. Acquired using maXis 4G.

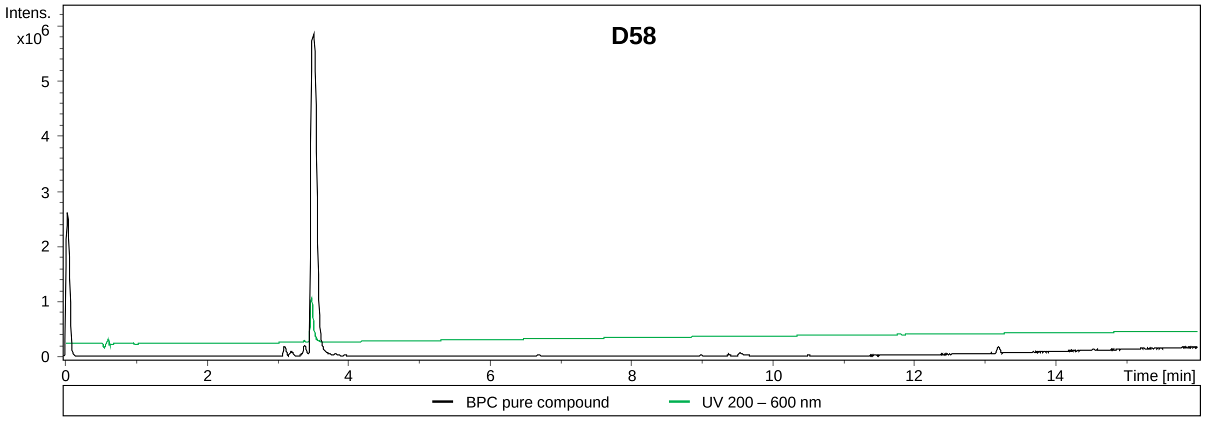


**Figure S 49: Chromatogram of purified D58.** The green trace shows the UV at 200–600 nm and the black trace shows the BPC. Acquired using maXis 4G.

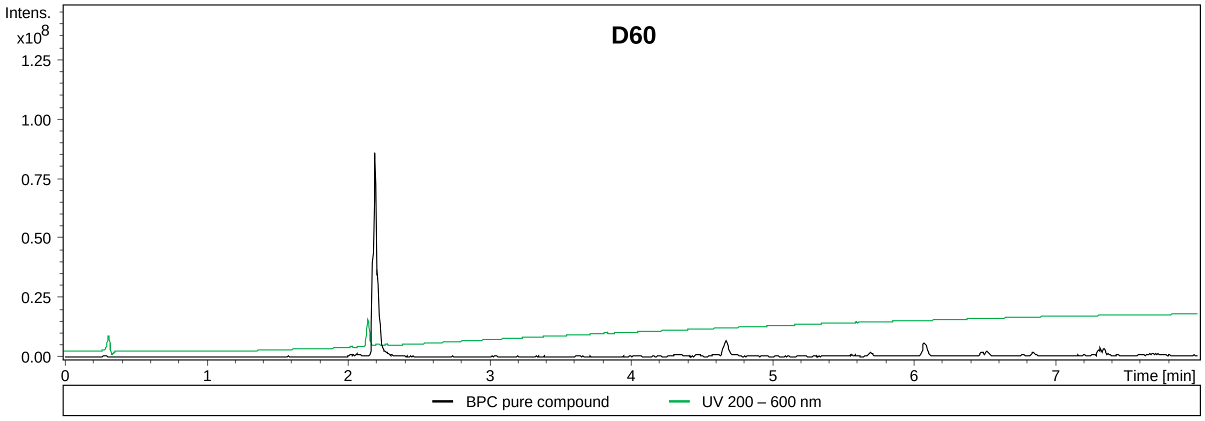


**Figure S 50: Chromatogram of purified D60.** The green trace shows the UV at 200–600 nm and the black trace shows the BPC. Acquired using amaZon speed.

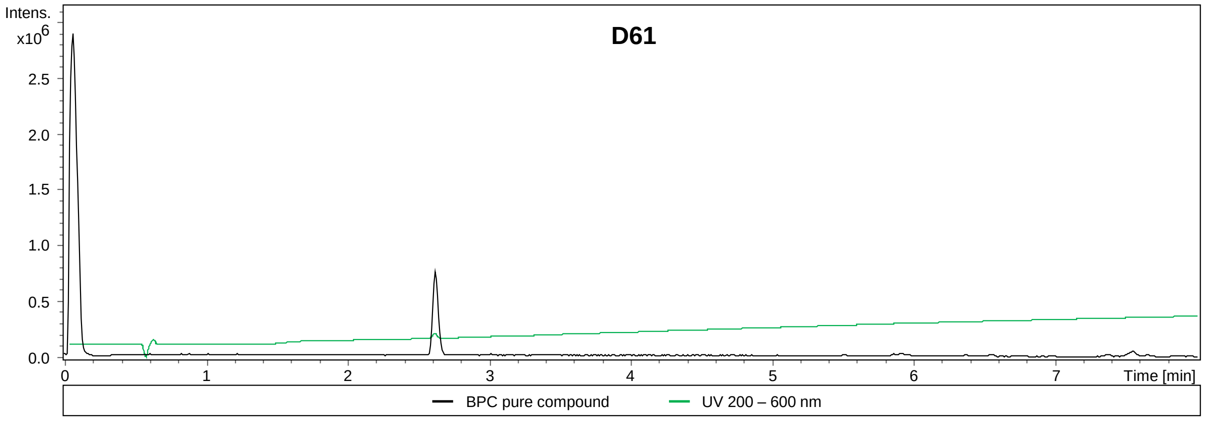


**Figure S 51: Chromatogram of purified D61.** The green trace shows the UV at 200–600 nm and the black trace shows the BPC. Acquired using maXis 4G.

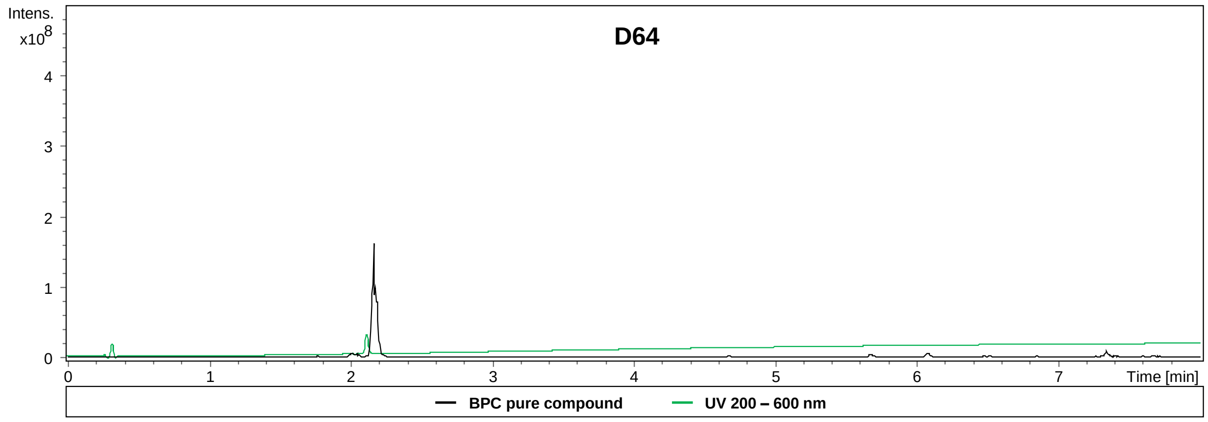


**Figure S 52: Chromatogram of purified D64.** The green trace shows the UV at 200–600 nm and the black trace shows the BPC. Acquired using amaZon speed.

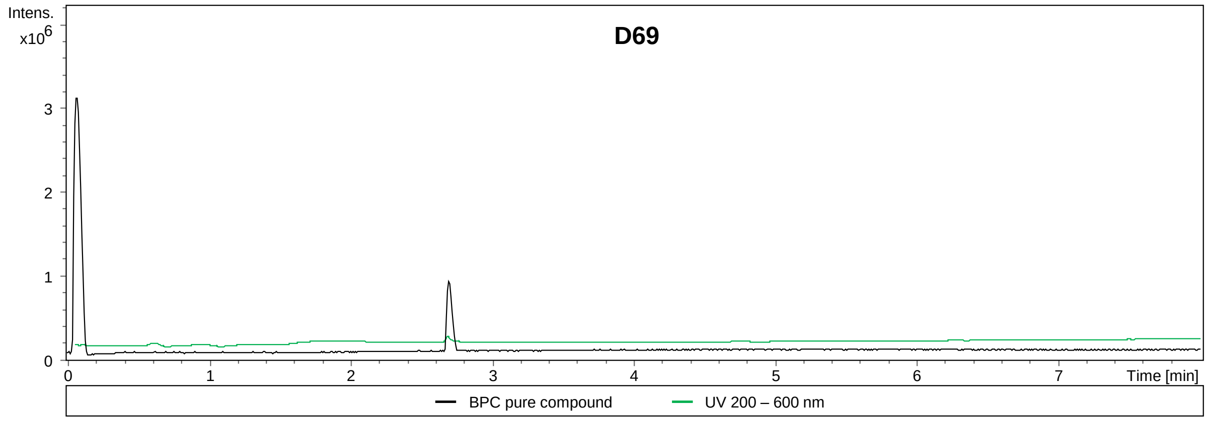


**Figure S 53: Chromatogram of purified D69.** The green trace shows the UV at 200–600 nm and the black trace shows the BPC. Acquired using maXis 4G.

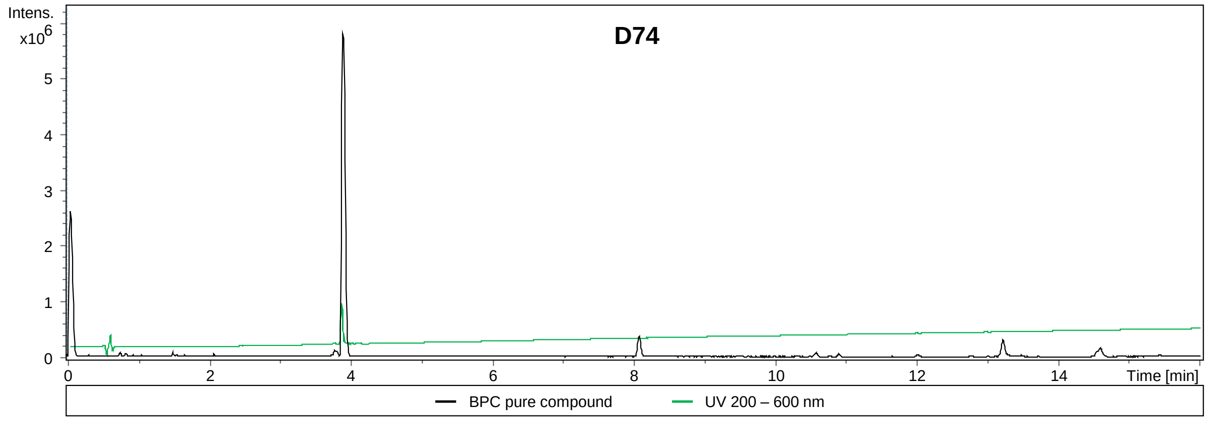


**Figure S 54: Chromatogram of purified D74.** The green trace shows the UV at 200–600 nm and the black trace shows the BPC. Acquired using maXis 4G.

References

(1) Goddard, T. D.; Huang, C. C.; Meng, E. C.; Pettersen, E. F.; Couch, G. S.; Morris, J. H.; Ferrin, T. E. UCSF ChimeraX: Meeting modern challenges in visualization and analysis. *Protein Science* **2018**, *27*, 14–25.

(2) Pettersen, E. F.; Goddard, T. D.; Huang, C. C.; Meng, E. C.; Couch, G. S.; Croll, T. I.; Morris, J. H.; Ferrin, T. E. UCSF ChimeraX: Structure visualization for researchers, educators, and developers. *Protein Sci.* **2021**, *30*, 70–82.

(3) Schrödinger. *The PyMOL Molecular Graphics System, Version 2.5.2*; Schrödinger, LLC, 2022.

(4) Pettersen, E. F.; Goddard, T. D.; Huang, C. C.; Couch, G. S.; Greenblatt, D. M.; Meng, E. C.; Ferrin, T. E. UCSF Chimera--a visualization system for exploratory research and analysis. *J. Comput. Chem.* **2004**, *25*, 1605–1612.

(5) Seyfert, C. E.; Porten, C.; Yuan, B.; Deckarm, S.; Panter, F.; Bader, C.; Coetzee, J.; Deschner, F.; Tehrani, K.; Higgins, P. G.*; et al.* Darobactins Exhibiting Superior Antibiotic Activity by Cryo-EM Structure Guided Biosynthetic Engineering. *Angew. Chem. Int. Ed.* **2022**, *62*, e202214094.

(6) Groß, S.; Panter, F.; Pogorevc, D.; Seyfert, C. E.; Deckarm, S.; Bader, C. D.; Herrmann, J.; Müller, R. Improved broad-spectrum antibiotics against Gram-negative pathogens via darobactin biosynthetic pathway engineering. *Chem. Sci.* **2021**, *12*, 11882–11893.
